# Modelling fishing‐induced evolution in pikeperch ( *Sander lucioperca* ) and vendace ( *Coregonus albula* ), Lake Oulujärvi, as template for ecosystem model

**DOI:** 10.1111/jfb.70028

**Published:** 2025-03-24

**Authors:** Eevi Kokkonen, Tommi Perälä, Laura S. Härkönen, Pekka Hyvärinen, Anna Kuparinen

**Affiliations:** ^1^ Department of Environmental and Biological Sciences, University of Eastern Finland Kuopio Finland; ^2^ Department of Biological and Environmental Science University of Jyväskylä Jyväskylä Finland; ^3^ Natural Resources Institute Finland (Luke) Migratory Fish and Regulated Rivers Paltamo Finland; ^4^ Natural Resources Institute Finland (Luke) Migratory Fish and Regulated Rivers Oulu Finland

**Keywords:** Allometric Trophic Network, fishing‐induced evolution, food web, planktivorous fish, top predator

## Abstract

Fishing‐induced evolution can impact fish trait distributions, with previous studies highlighting declines in size and age at maturation. However, the effects on fish growth remain less understood, and different fishing methods may exert distinct selection pressures on populations. This study explores the impact of gillnetting on pikeperch (*Sander lucioperca*, Percidae) and trawling on vendace (*Coregonus albula*, Coregonidae) modelled using data‐based normally distributed selection curves and the Allometric Trophic Network with Evolution model (Perälä & Kuparinen). Simulating evolutionary changes caused by fishing, we specifically examined the parameters of the von Bertalanffy model, including asymptotic length $\left(L^{\infty }\right)$ and Brody's growth coefficient $\left(k\right)$. In the model we simulated a negative correlation (−0.7) between asymptotic length and Brody's growth coefficient. We investigated the effects of parameters related to genotypic and phenotypic variance and the amount of correlation between asymptotic length and Brody's growth coefficient in the model using sensitivity tests. Trawling induces evolution in *C. albula* towards smaller asymptotic length, whereas Brody's growth coefficient stays at intermediate levels. The results for *C. albula* were consistent across different levels of correlation. Gillnetting of *S*. *lucioperca* results in evolution in asymptotic length and Brody's growth coefficient in variable directions. Frequently, *S. lucioperca* evolved towards larger size and growth, but depending on parametrization, selection can also be disruptive, or *S. lucioperca* may evolve towards smaller size and growth. The amount of genotypic and phenotypic variance also influences these outcomes, whereas instantaneous fishing mortality impacts biomasses across the food web. This study underscores the significance of considering fishing‐induced evolution, its impact on fish growth and food web–level effects, in addition to the densities of targeted species. Such insights are crucial for a comprehensive understanding of the ecological consequences of fishing practices.

## 1 INTRODUCTION

In today's world, humans have a profound impact on the environment, while nature continues to evolve at its own pace. Among these dynamics, fishing plays a significant role in shaping the lives of fish populations. As we cast our nets and lines into the water, we inadvertently trigger evolutionary changes in these aquatic communities. Fishing can also drive ecological changes within food webs, not only impacting targeted species but also indirectly affecting other levels of the ecosystem (e.g., Frank et al., 2005; Heithaus et al., 2008). In a trophic cascade, effects at higher levels of the food web propagate down to the lower levels through food web relationships (Carpenter et al., 1985). For example, abundant piscivore population controls planktivorous fishes, which reduces predation on zooplankton, leading to an increase in herbivory on phytoplankton (Carpenter et al., 1985). Correspondingly, fishing of piscivores can lower their abundance, and the effects can cascade down in the food web (Heithaus et al., 2008).

Fishing, as a selective force, can induce significant changes in natural populations (Fenberg & Roy, 2008; Heino et al., 2015). The selection of fishing gears, the timing of fishing and the locations where fishing occurs all contribute to its impact (e.g., Enberg et al., 2012). Measuring the exact selectivity of a fishing method can be challenging, as it is influenced not only by the method itself but also by the availability of fish of different traits within the population. Additionally, fisheries management further influences the selection process through regulations such as mesh‐size limitations, size limits, catch quotas and fishing enclosures (Baskett et al., 2005; Fenberg & Roy, 2008). Although fishing can exert selection pressures on multiple traits (Biro & Post, 2008; Hollins et al., 2018; Thambithurai et al., 2018), the bulk of research into fishing‐induced evolution has predominantly concentrated on its impact on size and the age of maturation (Heino et al., 2015). Following size‐selective fishing or high fishing mortality, fish populations often tend to shift towards smaller sizes and younger ages of maturation (Heino et al., 2015). The earlier maturation may be attributed to genetic factors or density‐dependent effects (Trippel, 1995). A smaller population size can reduce the amount of intraspecific competition, resulting in faster growth and earlier maturation (Trippel, 1995). Genetic changes may occur because late‐maturing fish would have fewer possibilities to reproduce compared to early‐maturing fish (Trippel, 1995).

Our study focuses on the genetic changes caused by fishery. The question of how fishing‐induced selection influences growth is a complex one, and it remains somewhat uncertain whether it favours faster or slower growth (Enberg et al., 2012). Growth is influenced by various factors related to resource acquisition and allocation (Enberg et al., 2012). Consequently, a multitude of underlying traits are subject to selection, such as fish behaviour or metabolism, with varying directions of response to selection (Hollins et al., 2018; Uusi‐Heikkilä et al., 2008). However, selection acts on fish phenotype (combination of traits influenced by multiple genes) (Biro & Post, 2008). An experiment by Conover and Munch (2002) showed that when fast‐growing or slow‐growing fish were selected, their offspring also displayed similar growth patterns. However, it is important to note that their experiment might represent an instance of particularly intense selection, whereas in natural environments, the selectivity of fisheries may be less stringent (Andersen & Brander, 2009). In another experiment, where selection was based on large or small size, or was random, fish selected for large size exhibited greater variation in their size especially when they were fed ad libitum (Uusi‐Heikkilä et al., 2016).

The von Bertalanffy growth model (VBGM; Bertalanffy, 1938), which originated from the earlier works of Pütter (1920) (for a detailed history of the model, see Kearney, 2021), is widely utilized in the study of fish growth. By fitting the model to data on fish lengths at different ages, fishery scientists obtain estimates of the asymptotic length ($L^{\infty }$) and the Brody's growth coefficient (k) within a population. However, the use of this model in the context of fish populations that are targeted by fisheries faced criticism due to the challenges in obtaining unbiased estimates from a harvested population (Ogle, 2016). Nonetheless, VBGM remains widely employed, with its parameters even integrated into the global fish species database FishBase (https://www.fishbase.se/search.php). Researchers also utilize VBGM in modelling studies to simulate individual growth (e.g., Kuparinen et al., 2014; Perälä & Kuparinen, 2020). VBGM can be applied at the species, population or individual level. In numerous studies, a negative correlation has been observed between $L^{\infty }$ and k (Charnov, 1993; Jensen, 1997; Thorson et al., 2017). Commonly, it is theorized that faster growth should result in a smaller asymptotic size, as rapidly growing individuals tend to mature earlier and allocate resources to reproduction. Likewise, slow‐growing individuals also reach maturity at some point, after which they have fewer resources available for further growth. Jensen (1997) offered a theoretical explanation for the expected negative correlation, suggesting that larger asymptotic length requires more energy for maintenance and movement, potentially leading to slower growth. Additionally, it is plausible that a smaller asymptotic size is reached more quickly. In this study, we specifically focus on the evolution of $L^{\infty }$ and k.

**FIGURE 1:**
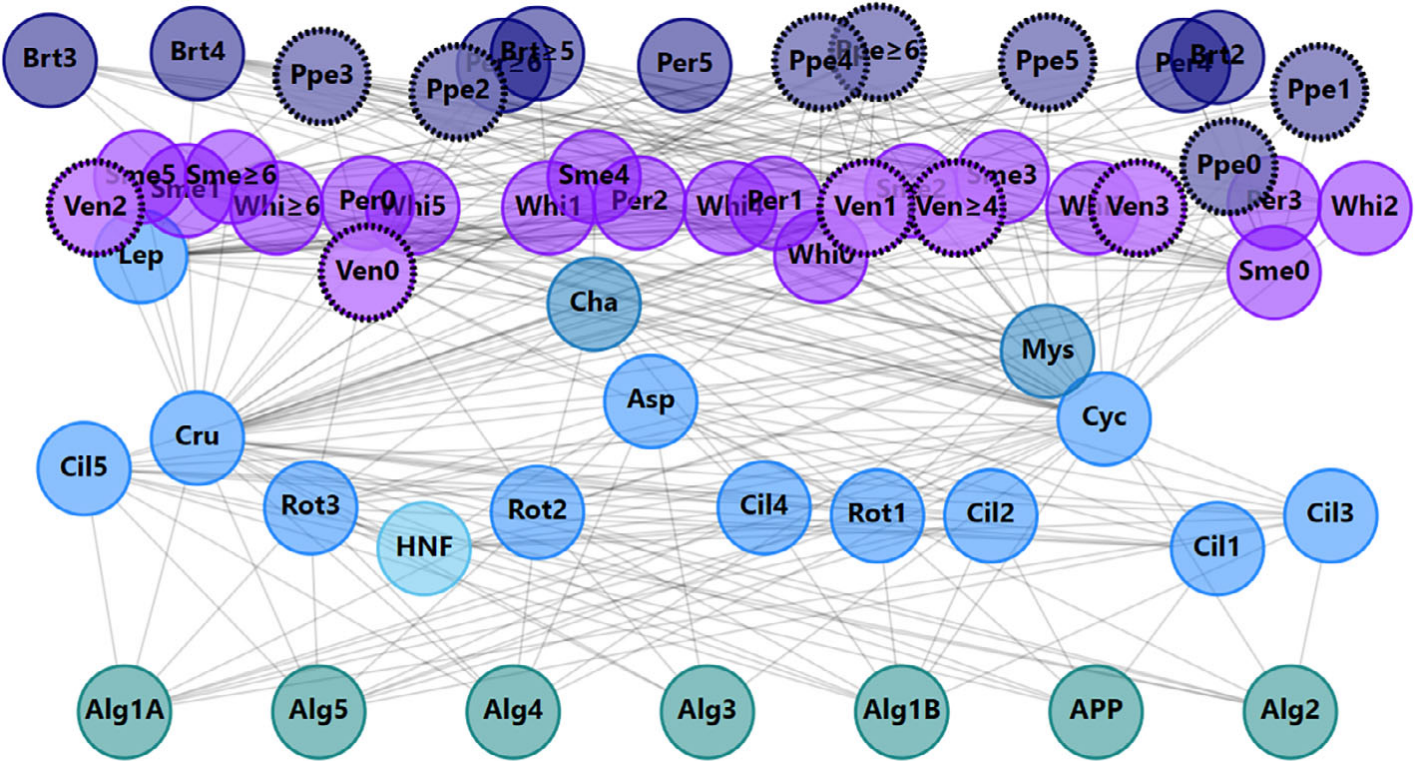
Lake Oulujärvi theoretical pelagic food web, including piscivorous fish (dark purple): *Sander lucioperca* (Ppe), *Salmo trutta* (Brt) and *Perca fluviatilis* (Per, age 4–age ≥6); planktivorous fish (purple): *Coregonus albula* (Ven), *Osmerus eperlanus* (Sme), *Perca fluviatilis* (Per, age 0–age 3) and *Coregonus lavaretus* (Whi) divided into age guilds; pelagic invertebrates (blue): *Mysis relicta* and *Chaoborus flavicans*, zooplankton (lighter blue): Ciliate guilds 1–5, rotifer guilds 1–3, *Asplancha priodonta*, Crustacean zooplankton, Cyclopoida zooplankton, *Leptodora* and other predatorous zooplankton guild, heterotrophic nanoflagellates (light blue) and algae guilds (green): 1a,1b, 2–5 and autotrophic picoplankton. The two fish species in the focus of our study, namely, *S. lucioperca* and *C. albula*, are highlighted with thick dashed black borders.

In a previous study, a theoretical Allometric Trophic Network (ATN) model of the pelagic food web in Lake Oulujärvi, the fifth largest lake in Finland, was constructed (Kokkonen, 2022; Kokkonen et al., 2024). This model serves as the foundation for the present study. Here, we employ the Allometric Trophic Network with Evolution (ATNE, Perälä & Kuparinen, 2020) model to investigate fishing‐induced evolutionary changes in the growth of two key species: pikeperch *Sander lucioperca* L. 1758, Percidae, and vendace *Coregonus albula* L. 1758, Coregonidae. We explore evolutionary changes occurring simultaneously in two species: the top predator fish, *S*. *lucioperca*, and a shoaling prey fish, *C. albula*. Both, *S*. *lucioperca* and *C. albula* hold significant commercial and recreational importance in the Finnish fishery, and Lake Oulujärvi plays a pivotal role in the fishery of these species. The *S*. *lucioperca* population in Lake Oulujärvi is estimated to be in relative good condition in relation to maximum sustainable yield based on biomass levels and mean lengths (Jakubavičiūtė et al., 2024). Commercial fishing of *C. albula* has declined since the 1990s, following a decrease in its size, whereas natural mortality caused by *S. lucioperca* has increased over the same period (Härkönen et al., 2023). Here we use the Lake Oulujärvi as a template lake ecosystem for our theoretical modelling study, due to the long time series of data available on fishing mortality and body size. Although the ATNE model has previously been employed to simulate evolution due to the selection of small or large European perch *Perca fluviatilis* L. 1758 in Lake Constance (Perälä & Kuparinen, 2020), this study focuses on the specific selectivity curves of different fishing methods, namely gillnets (with a mesh‐size of 50 mm) for *S*. *lucioperca* and trawl for *C. albula*. Additionally, we examine the effects of the selectivity of these fishing methods on the composition of the food web (Figure 1). Other fish species within the food web include smelt *Osmerus eperlanus* L. 1758, Osmeridae, whitefish *Coregonus lavaretus*, Valenciennes 1848, Coregonidae, *P*. *fluviatilis*, and brown trout *Salmo trutta* L. 1758, Salmonidae. For these other species, growth is modelled without evolutionary considerations and fishing, simplifying the model.

We introduced three different instantaneous fishing mortality values ($E=0.5\;y^{-1}$, $E=1.0\;y^{-1}$ and $E=2.0\;y^{-1}$, where *y* = year) to investigate how the severity of fishing mortality affected the growth parameters. We assumed high negative correlation (−0.7) between k and $L^{\infty }$ and additionally tested two levels of correlation: no correlation (0) and low negative correlation (−0.35). We hypothesized the following: (1) The simulation of fishing has a negative impact on the asymptotic length of *S*. *lucioperca* and *C. albula*. (2) The simulation of fishing has a negative or positive impact on the growth rate of *S*. *lucioperca* and *C. albula*. The nature of these effects 1 and 2 would be intricately linked to the shape of the selection curves and the strength of fishing mortality. (3) The effects of varying fishing mortality intensities also manifest in alterations to the composition of the food web.

## 2 METHODS

### 2.1 Ethics statement

This work is a modelling study for which we did not collect data. Data used for modelling were obtained from existing datasets collected for purposes of stock assessment and other research. Datasets have been collected according to Finnish legislation. More information on data collection practices can be found in Vehanen et al. (2020) and Härkönen et al. (2023).

### 2.2 ATNE model

In the ATNE model, the food web's complex dynamics are represented by a network of trophic interactions. These interactions encompass consumer‐resource relationships between functionally distinct guilds, including primary producers, consumers and various fish age guilds. The ATNE model is employed to simulate biomass dynamics within the food web while concurrently tracking the evolving life‐history traits of two fish species: *S. lucioperca* and *C. albula*. We chose this complex model to better capture the intricate relationships present in nature. Although it may be more difficult to isolate individual effects with such models, they provide a more accurate representation of the complexity faced in nature by reflecting the simultaneous influence of multiple factors.

To construct the ATNE model (Perälä & Kuparinen, 2020), we utilized the parametrization and food web relationships developed in our previous study (Kokkonen et al., 2024), where we had parameterized the ATN model for Lake Oulujärvi using data from the time period spanning from 1999 to 2018. This timeframe was selected due to the notable presence of a thriving *S*. *lucioperca* population. *S*. *lucioperca* had faced a severe decline in Lake Oulujärvi since the 1960s, attributed to factors such as overharvesting, reduction of reproduction areas and cooling climate (Colby & Lehtonen, 1994). In response to this decline, *S*. *lucioperca* stocking was initiated in 1985 (Salminen et al., 2012). Since 1999, *S*. *lucioperca* has been considered successfully re‐established in Lake Oulujärvi (Kokkonen et al., 2024).

Similar to its predecessor, the ATN model, ATNE model also considers consumption and maintenance as functions of the mass‐specific metabolic rates of consumers and fish. Metabolic rates, along with the intrinsic growth rates of producers, are allometrically scaled based on their carbon body masses. For fish, the carbon body mass is determined by the length of the fish, considering the length‐weight relationship and conversion factors between fresh weight, dry weight and carbon weight (Boit et al., 2012; Kokkonen et al., 2024; Kuparinen et al., 2016). In contrast to the ATN model, fish lengths in our model change dynamically based on their growth models, such that the fish grow along their von Bertalanffy curves during the growth season. Furthermore, to support the evolutionary dynamics of life‐history traits in selected fish species within the model, we employ dynamically evolving distributions to model traits associated with their body growth. The life‐history trait evolution is then induced by the application of size‐selective fishing, altering the trait distributions. The alterations in the trait distribution are then passed on to the offspring through a reproduction model implementing random mating (See also Perälä & Kuparinen, 2020 for a detailed description of the ATNE model.)

**FIGURE 2:**
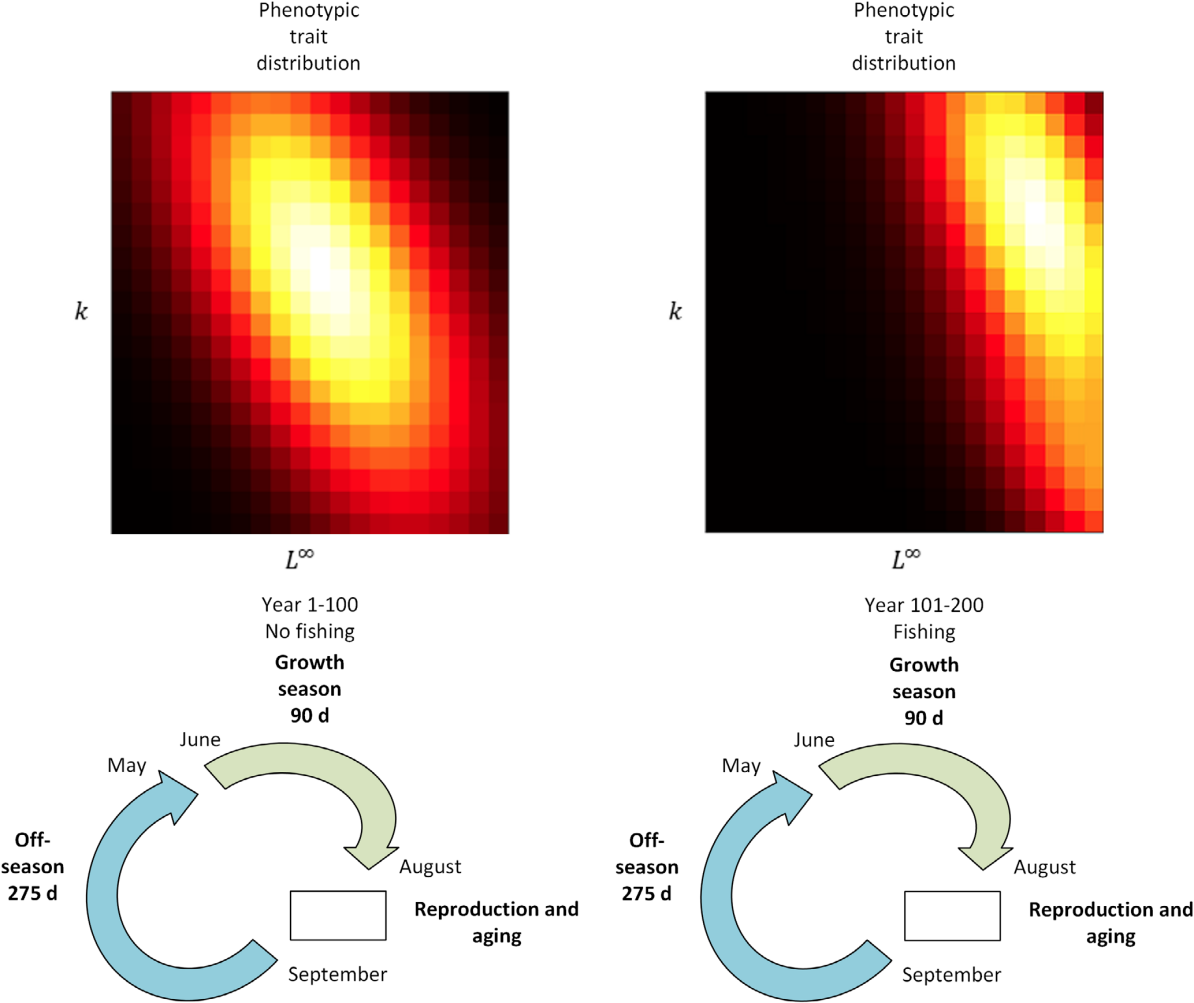
The model consists of three phases: Growth season, reproduction and ageing, and off‐season. There was the first 100‐year period without fishing for the system to attain the dynamic equilibrium and then the 100‐year period with fishing. In the absence of fishing, the trait distribution of k (Brody's growth coefficient) and $L^{\infty }$ (asymptotic length) stabilizes around the mean values. However, in the presence of fishing, the trait distribution shifts away from the mean due to selective pressures. Food web module operates during the growth season affecting biomasses through both gains and losses. The timing of reproduction and ageing is arbitrarily set in the autumn. Its primary purpose is at bookkeeping the biomasses and making the model ready for the next growth season.

The model's dynamics are divided into three distinct phases (Figure 2). First, during the ‘growth season’, a system of ordinary differential equations (ODEs) is solved. These ODEs capture the rates of biomass change for each guild, accounting for factors such as intrinsic growth, consumption, maintenance, reproductive investment and the removal of biomass through fishing activities. Following the growth season, the second phase, termed ‘reproduction and ageing’, involves the transfer of biomass within fish age guilds and the generation of new larvae based on the resources allocated for reproduction during the growth season. Finally, in the third phase, known as ‘off‐season dynamics', fish enter a resting state, during which they experience a reduced rate of biomass loss for the maintenance of bodily functions. This phase is followed by the commencement of a new year with a fresh growth season, thus repeating the annual cycle. ATNE model was run in Matlab R2023a (The Mathworks Inc., 2023).

### 2.3 The growth season dynamics

The biomass dynamics of guilds during the growth season of length $t^{\mathrm{end}}=90\;\left(\text{days}\right)$ are characterized by rates of biomass changes called gains (G) and losses (L) (refer to Tables 1 and 2 for a summary, and the Appendix for detailed descriptions).

**TABLE 1:** Gain terms for each type of guild in the food web.

		Gains	
Type		Intrinsic growth	Consumption
Equation		(S6)	(S7)
Producers		X	
Consumers			X
Fish	Larvae		X
	Juveniles		X
	Adults		X

**TABLE 2:** Loss terms for each type of guild in the food web.

		Losses			
Type		Maintenance	Consumption	Reproduction	Fishing
Equation		(S10, S11)	(S12, S13)	(S17)	(S15)
Producers			X		
Consumers		X	X		
Fish	Larvae	X	X		X^a^
	Juveniles	X	X		X^a^
	Adults	X	X	X	X^a^

^a^ In the model, the fishery exclusively targeted *Sander lucioperca* and *Coregonus albula*, with the catch contingent upon the size of the fish.

#### 2.3.1 Primary producer guild dynamics

The ODE describing the dynamics of the primary producer guild i includes gains from intrinsic growth (Equation S6) and losses due to consumption by its consumers (Equation S12):

(1) $${\dot{B}}_{i}\left(t\right)=G_{i}^{\text{growth}}\left(t\right)-\sum_{j\in I_{i}^{\text{consumers}}}L_{i,j}^{\text{consumption}}\left(t\right),$$

Here $\dot{B_{i}}\left(t\right)$ is the derivative of the biomass of guild i with respect to time, and $I_{i}^{\text{consumers}}$ denotes the set of indices for the consumer guilds of the producer guild i.

#### 2.3.2 Consumer guild dynamics

The ODE describing the dynamics of consumer guild i consists of gains from the consumption of its resources, $I_{i}^{\text{resources}}$ (Equation S7), losses due to maintenance of bodily functions (Equation S10), and losses from being consumed by its predators, $I_{i}^{\text{predators}}$ (Equation S12):

(2) $${\dot{B}}_{i}\left(t\right)=\begin{array}{l} \left(\sum_{j\in I_{i}^{\text{resources}}}G_{i,j}^{\text{consumption}}\left(t\right)\right)-L_{i}^{\text{maintenance}}\left(t\right)\\ -\;\left(\sum_{k\in I_{i}^{\text{predators}}}L_{i,k}^{\text{consumption}}\left(t\right)\right). \end{array}$$

#### 2.3.3 Fish age guild dynamics

For fish, age guilds are composed of age classes or age groups containing several age classes; hereafter they are called age guilds, as they are functional guilds in the food web. In addition to the processes governing consumer guild dynamics, the ODE for fish age guild i accounts for losses due to fishing (Equation S15) and, as the model separately keeps track of the biomass invested into reproduction, losses due to biomass allocated to reproduction (Equation S17). Moreover, for the purposes of the fish life‐history evolution in the model, two of the fish species have been structured to consist of different ‘genotype groups’, each, and thus we present the fish dynamics with an additional subscript g denoting the genotype group of guild i $\left({\mathrm{GG}}_{i,g}\right)$

(3) $${\dot{B}}_{i,g}\left(t\right)=\begin{array}{l} \left(\sum_{j\in I_{i}^{\text{prey}}}G_{i,g,j}^{\text{consumption}}\left(t\right)\right)-L_{i,g}^{\text{maintenance}}\left(t\right)\\ -\;\left(\sum_{k\in I_{i}^{\text{predators}}}L_{i,g,k}^{\text{consumption}}\left(t\right)\right)-L_{i,g}^{\text{fishing}}\left(t\right)-L_{i,g}^{\text{reproduction}}\left(t\right), \end{array}$$

where $I_{i}^{\text{prey}}$ denotes the indices of the prey guilds of fish age guild i.

#### 2.3.4 Reproductive output dynamics

Finally, as stated above, the biomass allocated to reproduction is tracked separately in the model, and the ODE for the contribution of ${\mathrm{GG}}_{i,g}$ to the reproductive output is as follows:

(4) $${\dot{B}}_{i,g}^{+}\left(t\right)=L_{i,g}^{\text{reproduction}}\left(t\right)-\frac{B_{i,g}^{+}\left(t\right)}{B_{i,g}\left(t\right)}L_{i,g}^{\text{fishing}}\left(t\right),$$

where $B_{i,g}\left(t\right)$ is the biomass of ${\mathrm{GG}}_{i,g}$.

### 2.4 Reproduction and ageing

In the model, reproduction and ageing of the fish are set to occur during the off‐season, with all reproduction events consolidated in autumn. Although this timing is not biologically accurate for all species, it is a technical simplification in the model. Because the model is only concerned with the amount of larvae present at the beginning of the growth season, this approach suffices for capturing the relevant dynamics (Figure 2). After the growth season of year Y, new fish larvae are generated. The total reproductive output used for the generation of the biomass of fish larvae guild i, $B_{Y,i}^{+}$, is calculated by summing up over all age guilds belonging to the same species and each genotype group,

(5) $$B_{Y,i}^{+}=\sum_{a=0}^{a_{\max,i}}\sum_{g=1}^{G}B_{Y,i+a,g}^{+}\left(t^{\mathrm{end}}\right),$$

where $a_{\max,i}$ is the maximum age guild of the species to which the larvae guild i belongs, and G=10,000 is the total number of genotype groups in the model for *S*. *lucioperca* and *C. albula*, and G=1 for other fish species. The total fish larvae guild i biomass becomes ${\hat{B}}_{Y,i}=uB_{Y,i}^{+}$, where u=0.8 is the efficiency at which the biomass allocated to reproduction is converted into larvae biomass. However, as *S. trutta* is, in general, not able to reproduce naturally in the system, 2‐year‐old individuals are stocked to maintain the population, and we set ${\hat{B}}_{Y,i}=137.3$ for stocking the *S. trutta* age 2 for all years. In reality, there are still a few reproduction areas left, but efficient fishing removes the *S. trutta* already before they reach their maturation age (Härkönen et al, 2023).

The distribution of the larvae biomass among the different genotype groups is determined based on the distribution of the biomass allocated to reproduction by different genotype groups within the adult age guilds of that species. The total reproductive output of each ‘genotype lineage’ $\left(i_{*},g\right)$, that is, genotype groups g from all age guilds of a given species, $B_{Y,i_{*},g}^{+}$, is first obtained by summing up the reproductive outputs of all age guilds,

(6) $$B_{Y,i_{*},g}^{+}=\sum_{a=0}^{a_{\max,i}}B_{Y,i+a,g}^{+}\left(t^{\mathrm{end}}\right),$$

Next, a discrete probability distribution of the parent trait vector, $T^{P}$, based on the genotype groups in the reproductive output is obtained by dividing the genotype lineage outputs by the total output, $P\left(T^{P}\right)=B_{Y,i_{*},g}^{+}/B_{Y,i}^{+}$. The conditional probability distribution of the larvae trait (traits: k and $L^{\infty }$) vector $T^{L}$ conditioned on a pair of parent trait vectors $\left(T^{P_{1}},T^{P_{2}}\right)$ is modelled as a multivariate normal distribution,

(7) $$p\left(T^{L}|T^{P_{1}},T^{P_{2}}\right)=\mathrm{MVN}\left(T^{L};\mu ,\sum \right),$$

where the mean, $\mu =E\left(T^{P_{1}},T^{P_{2}}\right)$, is the mean of the parent trait vectors, and the covariance matrix $\sum =\Omega^{\frac{1}{2}}\Lambda \Omega^{\frac{1}{2}}$, where the uncorrelated variance matrix, $\Omega =\Omega_{G}+\Omega_{A}$, consists of a genotypic component $\Omega_{G}=c^{V}\mathrm{diag}\left(V\left(T^{P_{1}},T^{P_{2}}\right)\right)$, which is a diagonal matrix with the variances of the parent trait values multiplied by a free parameter $c^{V}$, and a phenotypic component, a free parameter diagonal matrix $\Omega_{A}=\mathrm{diag}\left({\tilde{\sigma }}_{L^{\infty }}^{2},{\tilde{\sigma }}_{k}^{2}\right)$. The amount of genotypic variation is controlled by $c^{V}$, whereas $\sum^{A}$ is used to control the amount of phenotypic variation. The correlation matrix $\Lambda =\left[\begin{array}{cc} 1 & \rho \\ \rho & 1 \end{array}\right]$ includes a free parameter, the correlation coefficient ρ between $L^{\infty }$ and k. Using the law of total probability and assuming independence between the trait values of the parents, the probability distribution of the larvae trait values can be written as

(8) $$p\left(T^{L}\right)=\sum_{T^{P_{1}}}\sum_{T^{P_{2}}}p\left(T^{L}|T^{P_{1}},T^{P_{2}}\right)P\left(T^{P_{1}}\right)P\left(T^{P_{2}}\right)$$

We are using discrete probability distributions for the parent trait values, represented by a grid in the trait space, and thus the larvae trait distribution becomes a mixture of multivariate normal distributions that are truncated to the grid. Finally, we discretize the larvae trait distribution using the grid cells by calculating how much probability mass of the mixture distribution falls within each cell (i.e., genotype group) using the cumulative distribution function of the multivariate normal distribution; thus the total reproductive output is distributed across the trait grid cells based on the respective probability mass. The grid boundaries represent the upper and lower limits for the trait values in the simulation and are fixed beforehand.

The juvenile and adult fish guilds' biomasses move up one age guild and retain their genotype group assignments.

(9) $${\hat{B}}_{Y,i,g}=B_{Y,i-1,g}\left(t^{\mathrm{end}}\right)$$

The age guild $a_{\max}$ consists of fish of age $a_{\max}$ years and older, and its biomass after accounting for ageing is the sum of the biomasses of the previous age guild and the biomass of the maximum age guild at the end of the growth season of year Y.

(10) $${\hat{B}}_{Y,i,g}=B_{Y,i,g}\left(t^{\mathrm{end}}\right)+B_{Y,i-1,g}\left(t^{\mathrm{end}}\right)$$

### 2.5 Off‐season dynamics

For the non‐fish guilds, the initial biomass for the year Y+1 is their biomass at the end of the growth season of year Y. For the fish guilds, we apply the off‐season maintenance losses to arrive at the initial biomass for the next growth season.

(11) $$B_{Y+1,i,g}\left(0\right)=e^{-f_{m}^{*}x_{i,g}\left(365-t^{\mathrm{end}}\right)}{\hat{B}}_{Y,i,g},$$

where $f_{m}^{*}=\frac{1}{2}f_{m}$ is the off‐season maintenance respiration coefficient.

### 2.6 Parameterizing the fish growth models

#### 2.6.1 Fitting Bayesian VBGM to empirical data

A considerable amount of empirical datasets collected from the Lake Oulujärvi fish have been prepared by researchers at Kainuu Fisheries Research Station (Härkönen & Hyvärinen, 2024a, 2024b, 2024c, 2024d, 2024e, 2024f, 2024g, 2024h), including *S*. *lucioperca*: (Vainikka & Hyvärinen, 2012) and *C. albula* (Huusko & Hyvärinen, 2005). For a comprehensive overview of data collection practices, see Vehanen et al. (2020) and Härkönen et al. (2023).

We initiated the process of parameterizing our fish growth models by fitting a Bayesian von Bertalanffy growth model (BVBGM) to the length‐at‐age data. The lengths of fish had been measured and ages determined. We estimated the ‘fractional age’, that is, the age in completed years of growth and the fraction of the current year's growth for fish by assuming 1st June as their date of birth and by estimating how many years and days the age was in the day of capture. The posterior distributions of model parameters obtained from fitting the BVBGM models were then used for subsequent simulations within our ATNE model.

The BVBGM describes the growth of fish in terms of their age, asymptotic maximum length ($L^{\infty }$), Brody's growth coefficient $\left(k\right)$ and length at the time of birth $\left(L^{0}\right)$. The length $L_{t}$ of the fish given their fractional age $a_{t}$ and the unknown parameters of VBGM: $\left(L^{\infty },k,L^{0}\right)$ was modelled using a Lognormal distribution:

(12) $$L_{t}|a_{t},L^{\infty },k,L^{0}\sim \text{Lognormal}\left(\mu_{t},\sigma \right),$$

where the location parameter of the lognormal distribution was expressed in terms of the expected value of the distribution

(13) $$\mu_{t}=E\left(L_{t}|a_{t},L^{\infty },k,L^{0}\right)-\frac{1}{2}\sigma^{2},$$

which was modelled using the VBGM:

(14) $$E\left(L_{t}|a_{t},L^{\infty },k,L^{0}\right)=L^{\infty }-\left(L^{\infty }-L^{0}\right)e^{-ka_{t}}.$$

We used uniform priors for the parameters of the BVBGM:

(15) $$L^{\infty }\sim \text{Uniform}\left(L_{\mathrm{low}}^{\infty },L_{\mathrm{up}}^{\infty }\right),$$

(16) $$k\sim \text{Uniform}\left(k_{\mathrm{low}},k_{\mathrm{up}}\right),$$

(17) $$L^{0}\sim \text{Uniform}\left(L_{\mathrm{low}}^{0},L_{\mathrm{up}}^{0}\right),$$

for which the lower and upper limits are described in the Appendix. For the shape parameter of the lognormal distribution, we used a uniform prior:

(18) $$\sigma \sim \text{Uniform}\left(\sigma_{\mathrm{low}},\sigma_{\mathrm{up}}\;\right).$$

The model was implemented in Stan (Stan Development Team, 2022) and the inference carried out in R 4.2.1 (R Core Team, 2022) using the RStan interface (Stan Development Team, 2023). We generated 4 MCMC chains using a warmup period of 1000 and recording the next 4000 samples for each chain.

In our ATNE model, we simulated the evolution of fish life‐history traits for *S*. *lucioperca* and *C. albula*, specifically focusing on changes in the $L^{\infty }$ and k parameters. The upper and lower limits for the trait values in the simulation, represented as grid boundaries, were defined after fitting BVBGM to understand the range of parameters within the population. These limits were chosen to encompass the observed range and provide space for potential evolutionary changes in these parameters. For the remaining other fish species in the model, we characterized them as ‘average individuals’ with their deterministic VBGMs parameterized using posterior mean estimates obtained from fitting their BVBGMs. In some cases, the Bayesian modelling resulted in very narrow posterior distributions for $L^{\infty }$, perhaps because we had such large data sets (*C. albula*, *C. lavaretus*, *O. eperlanus*). Additionally, some models produced exceptionally large $L^{0}$ values, prompting us to approximate these values with insights obtained from the existing literature. The specific estimates chosen for integration into the ATNE model are shown in Table 3. See also the Appendix for further information.

**TABLE 3:** Parameters of the von Bertalanffy model (Equation S5) for the fish species *Coregonus lavaretus*, *Perca fluviatilis*, *Osmerus eperlanus*, *Coregonus albula*, *Sander lucioperca* and *Salmo trutta* in the model based on the posterior means of the Bayesian von Bertalanffy model fit to the population‐level data assumed to represent an average individual of the species.

Species	Years	N	$L^{\infty }\;\left(\mathrm{cm}\right)$	$k\;\left({\text{year}}^{-1}\right)$	$L^{0}\;\left(\mathrm{cm}\right)$
*Coregonus lavaretus*	1972–2017	49,682	33	0.277	1.1^a^
*Perca fluviatilis*	2007, 2012	350	71	0.057	1.7
*Osmerus eperlanus*	1989, 1994–2017	9240	17	0.171	1.4^b^
*C. albula*	1973–1984, 1986–2017	20,026	16 [10 20]	0.555 [0.45 0.65]	0.9^c^
*S. lucioperca*	1973–1975, 1988–1990, 1992–2018	11,554	87 [70 90]	0.124 [0.1 0.2]	1.7
*Salmo trutta*	1995–1997, 2001–2002	608	83	0.198	1.7

*Note*: For *S*. *lucioperca* and *C. albula* the limits of the evolving trait values are also shown. Also shown are the years of the observations and the number of individuals recorded. $L^{0}$ for *C. lavaretus*, *C. albula* and *O. eperlanus* were adjusted based on literature. *P. fluviatilis* $L^{0}$ was adjusted to similar value as *S*. *lucioperca*. $L^{0}$ estimated from ^a^Sutela and Huusko (2000), ^b^Sutela and Hyvärinen (2002), ^c^Sutela and Huusko (1997), and Sutela and Huusko (2000).

### 2.7 Modelling fishing gears and their selectivity

Empirical datasets from Lake Oulujärvi were utilized for modelling fishing gear selectivity, encompassing information on *S*. *lucioperca* (Vainikka & Hyvärinen, 2012) and *C. albula* (Suuronen et al., 1995). For a broader perspective on data collection practices, consult Vehanen et al. (2020) and Härkönen et al. (2023). To explore the impact of fishing on the eco‐evolutionary dynamics in our ATNE model, we implemented size‐selective fishing gears to simulate the removal of fish biomass. Specifically, we employed gillnets for *S*. *lucioperca* fishing and trawling for *C. albula*. The selectivity of both gears was modelled using a bell‐shaped curve,

(19) $$\hat{S}\left(L;\mu ,\sigma ,\delta \right)=\frac{\delta }{\sqrt{2\pi }\sigma }e^{-\frac{{\left(L-\mu \right)}^{2}}{2\sigma^{2}}},$$

which was fitted to the length class–fished biomass data, achieved by minimizing the sum of squared residuals through the fminsearch function in Matlab R2021 (The Mathworks Inc., 2021). In the equation δ is a scaling parameter. This method allowed us to capture the selectivity patterns associated with each fishing gear and their consequential effects on the studied fish populations.

**FIGURE 3:**
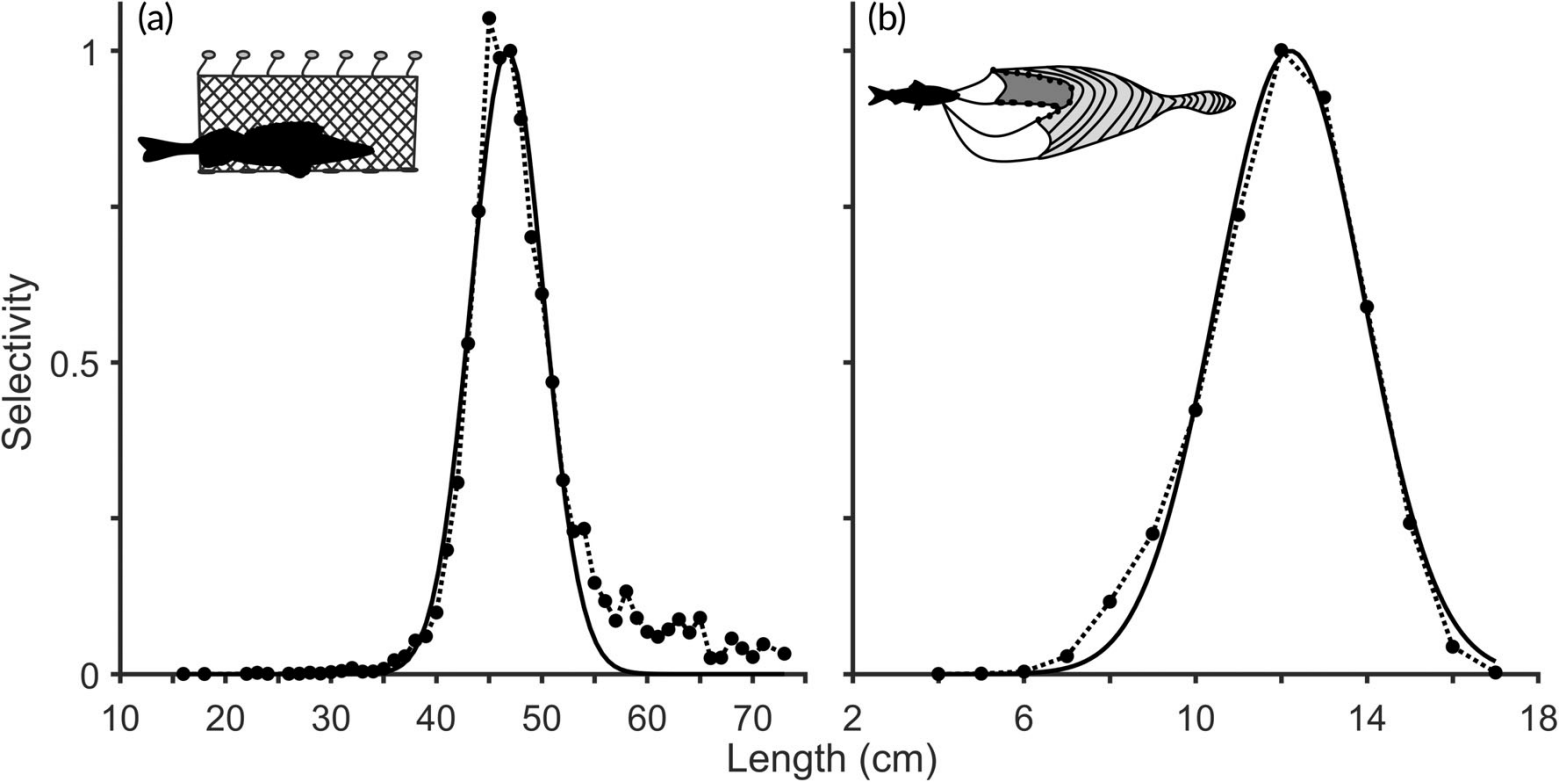
Selectivity curves for 50 mm gillnets targeting *Sander lucioperca* (a) and *Coregonus albula* trawls (b). The horizontal axis represents length in centimetres. Black dots connected by dotted lines depict aggregated fished biomass for various length classes. The black solid lines represent the modelled selectivity.

We selected gillnets as the method for modelling *S*. *lucioperca* fishing, recognizing their selective nature (Hamley, 1975) and the common practice of mesh‐size management for real‐world fishing impact control. Our modelling focused on data from mesh‐size 50 mm (knot length), which had the largest‐caught biomass among different knot sizes in the available dataset, spanning years 1990, 1992, 1994–2010, 2012–2016 and 2018, and comprising 2427 individuals. *S. lucioperca* was categorized into length classes (e.g., 20 cm length class covering lengths from 20 to 20.9 cm), with a minimum length class of 16 cm and a maximum of 73 cm. The fished biomass (in grams) of *S*. *lucioperca* using this mesh‐size for each length class was aggregated. The estimates for *S*. *lucioperca* selectivity curve (μ=46.72cm and σ=3.44cm) were utilized to construct the selectivity curve for the gillnet fishery (Figure 3).

For *C. albula*, trawling was employed as the fishing method. We utilized all available trawl data, consolidating diverse trawling gears (square/diamond; mesh‐sizes 10–15 mm, with square and mesh‐size 12 mm being the most prevalent) to enhance data coverage and gain a comprehensive understanding of trawling effects. The dataset covered the years 2001–2004, involving 14,330 individuals with recorded mass and length. The length classes considered ranged from 4 to 17 cm. Parameter estimates for the selectivity curve were μ=12.21cm and σ=1.71cm (Figure 3).

### 2.8 Variance and heritability of the traits in the populations

To evaluate the impact of various parameters of the reproduction model – specifically, the additive phenotypic variance components ${\tilde{\sigma }}_{L^{\infty }}^{2}$ and ${\tilde{\sigma }}_{k}^{2}$, and the genotypic variance control parameter $c^{V}$ – on model dynamics, we examined the variance and heritability of the evolving traits. We computed the larval trait distribution with (phenotype) and without (genotype) the additive phenotypic variance component. This allowed us to derive marginal variances for the genotype, namely, $V_{G}\left(L^{\infty }\right)$ and $V_{G}\left(k\right)$, and for the phenotype, namely $V_{P}\left(L^{\infty }\right)$ and $V_{P}\left(k\right)$. The heritability of these traits was defined as the genotypic variance divided by the phenotypic variance, that is, $H=\frac{V_{G}}{V_{P}}$. With this calculation, one can obtain heritability values over one.

### 2.9 Simulation set‐up

During the simulations, we initiated a 100‐year burn‐in period without fishing to allow the system to attain dynamic equilibrium concerning biomasses and the evolving life‐history traits of *S. lucioperca* and *C. albula* (Figure 2). In the equilibrium without fishing, biomasses stayed around mean values as exemplified in Figure 2. Subsequently, we simulated an additional 100 years in the presence of a fishery targeting both *S*. *lucioperca* and *C. albula* (Figure 2). Other fish species were not targeted by the fishery.

In our simulations, we examined two aspects. The ‘baseline’ scenario aimed to evaluate the eco‐evolutionary impacts of size‐selective fishing at the food web level by comparing equilibrium biomasses of organismal groups and guilds under unfished conditions with those under various fishing intensities. Additionally, we investigated the sensitivity of model dynamics to the parameterization of the reproduction model. Because the true values of these parameters are unknown, we conducted a sensitivity analysis, defining ‘low’, ‘medium’, and ‘high’ values (Table 4). We systematically varied the parameters, exploring all possible combinations for *S*. *lucioperca* or *C. albula*, while maintaining fixed values (medium) for the other species: *S*. *lucioperca* or *C. albula*. The sensitivity analysis encompassed 81 distinct parameter combinations for both species. In the baseline scenario, we employed ‘medium’ parameter values for the reproduction model. Sensitivity analyses were conducted thrice to examine the effects of different levels of correlation; high negative correlation (ρ=−0.7), low negative correlation (ρ=−0.35) or no correlation (ρ=0). Although negative correlation was the most justified assumption based on literature (Charnov, 1993; Jensen, 1997; Thorson et al., 2017), we also wanted to see the effect of no correlation option as there seem to be not many studies on the relationship between Brody's growth coefficient and asymptotic length at the individual level. We tested three different instantaneous fishing mortality values ($E=0.5\;y^{-1}$, $E=1.0\;y^{-1}$ and $E=2.0\;y^{-1}$, where *y* = year). Instantaneous fishing mortality values were chosen arbitrarily, but they represent possible values observed in Lake Oulujärvi time series depending on year and age class concerned. Yearly average values of instantaneous fishing mortality for harvestable size *S. lucioperca* have varied between 0.09 and 1.22 (Härkönen & Hyvärinen, 2024a). Yearly average values of instantaneous fishing mortality for most abundant age classes available for harvest of *C. albula* have varied between 0.06 and 2.2 (Härkönen & Hyvärinen, 2024c).

**TABLE 4:** Parameter values during the sensitivity analysis.

Parameter	Low	Medium	High
E	0.5	1.0	2.0
$c^{V}$	0.125	0.25	0.5
${\tilde{\sigma }}_{L^{\infty },\mathrm{Ppe}}$	1.5	3.0	6.0
${\tilde{\sigma }}_{k,\mathrm{Ppe}}$	0.015	0.030	0.060
${\tilde{\sigma }}_{L^{\infty },\mathrm{Ven}}$	0.5	1.0	2.0
${\tilde{\sigma }}_{k,\mathrm{Ven}}$	0.01	0.02	0.04

*Note*: E= Maximum instantaneous fishing mortality, $c^{V}=$ Genotypic variance control parameter, ${\tilde{\sigma }}_{L^{\infty }}=$ Additive phenotypic variance parameter for asymptotic length, ${\tilde{\sigma }}_{k,\mathrm{Ppe}}=$ Additive phenotypic variance parameter for Brody's growth coefficient. ‘Ppe’ refers to *Sander lucioperca*, and ‘Ven’ refers to *Coregonus albula*.

## 3 RESULTS

### 3.1 Evolution of growth parameters

The sensitivity of the trait distribution to variations in phenotypic and genotypic variance parameters (${\tilde{\sigma }}_{L^{\infty }}$ and ${\tilde{\sigma }}_{k}$, and $c^{V}$, respectively), along with the impact of fishing mortality severity controlled by instantaneous fishing mortality (E), was examined for the oldest fish age guilds (*S*. *lucioperca* age ≥6 and *C. albula* age ≥4). Larger values of the variance parameters resulted in increased variability in the trait distribution. The correlation coefficient ρ between k and $L^{\infty }$ influenced the shape of the trait distribution, and, in *S. lucioperca*, also affected the location of the traits.

**FIGURE 4:**
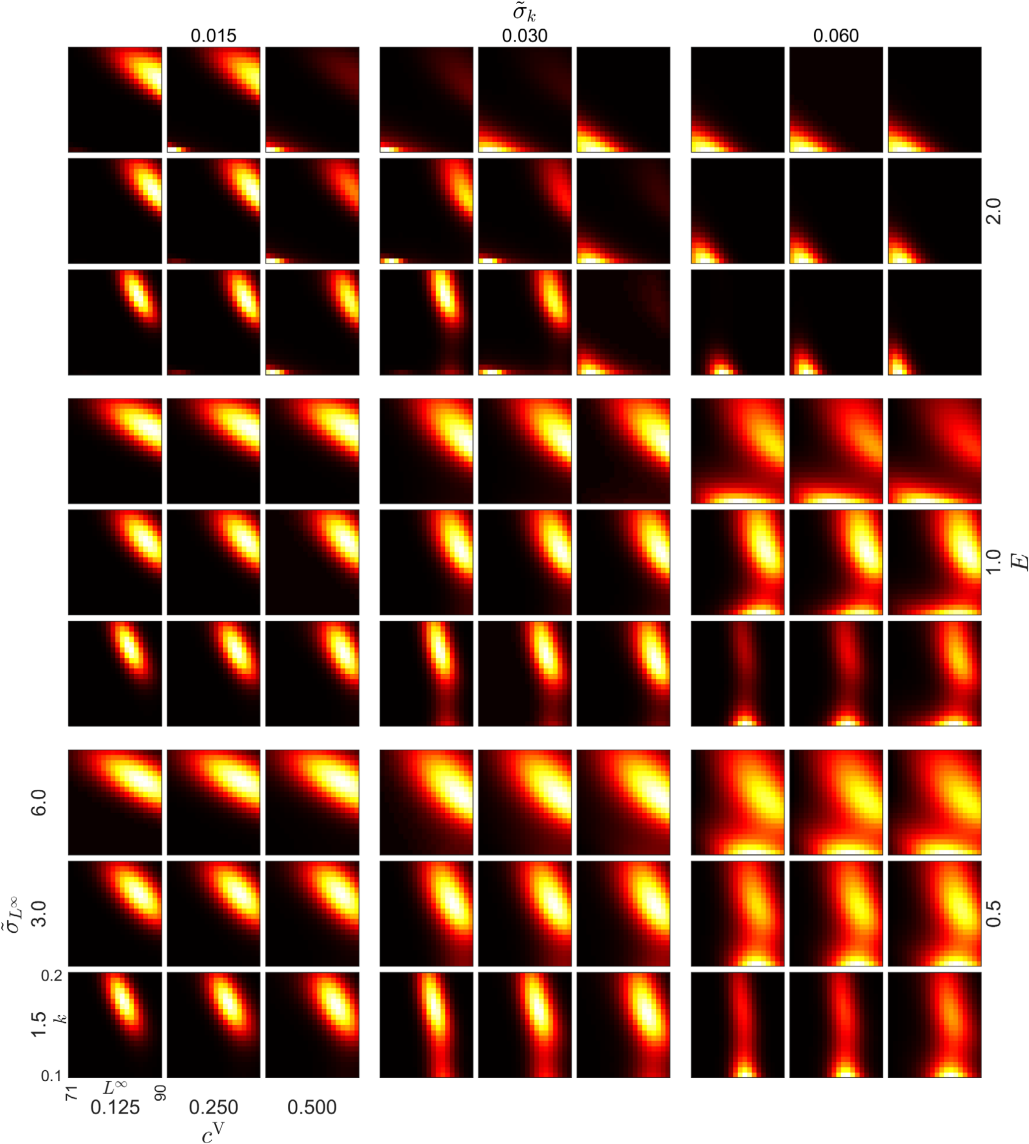
*Sander lucioperca* age 6+ trait distribution after 100 years of size‐selective fishing in the simulation, with a high (ρ = −0.7) level of correlation between k (Brody's growth coefficient) and $L^{\infty }$ (asymptotic length). The figure illustrates the influence of varying instantaneous fishing mortalities $\left(E\right)$, and genotypic $\left(c^{V}\right)$ and phenotypic $\left({\tilde{\sigma }}_{L^{\infty }},{\tilde{\sigma }}_{k}\right)$ variance parameters on the eco‐evolutionary impacts of size‐selective fishing. The horizontal axis ($L^{\infty }$) represents the asymptotic length (cm), and the vertical axis (k) represents Brody's growth coefficient ($y^{-1}$). Brighter colours indicate higher trait density. Mean of the starting distribution is in the middle of the grid.

For *S*. *lucioperca*, the direction of the evolution of growth was influenced by the severity of fishing mortality, the parameters governing the amount of evolvable variation and the strength of the correlation between k and $L^{\infty }$ (Figures 4 and S19, S20). Depending on parametrization the selection could favour fast growth and large size, be disruptive or favour small size and slow growth under high fishing mortality ($E=2.0\;y^{-1}$) (Figure 4). At lower instantaneous fishing mortalities ($E=1.0\;y^{-1}$ and $E=0.5\;y^{-1}$), evolution in growth also exhibited variability. Generally, evolution trended towards larger k and $L^{\infty }$ values with lower instantaneous fishing mortality (Figure 4). Disruptive selection was observed towards both smaller and larger trait values at lower instantaneous fishing mortality especially when ${\tilde{\sigma }}_{k}$ was 0.060 (Figure 4).

**FIGURE 5:**
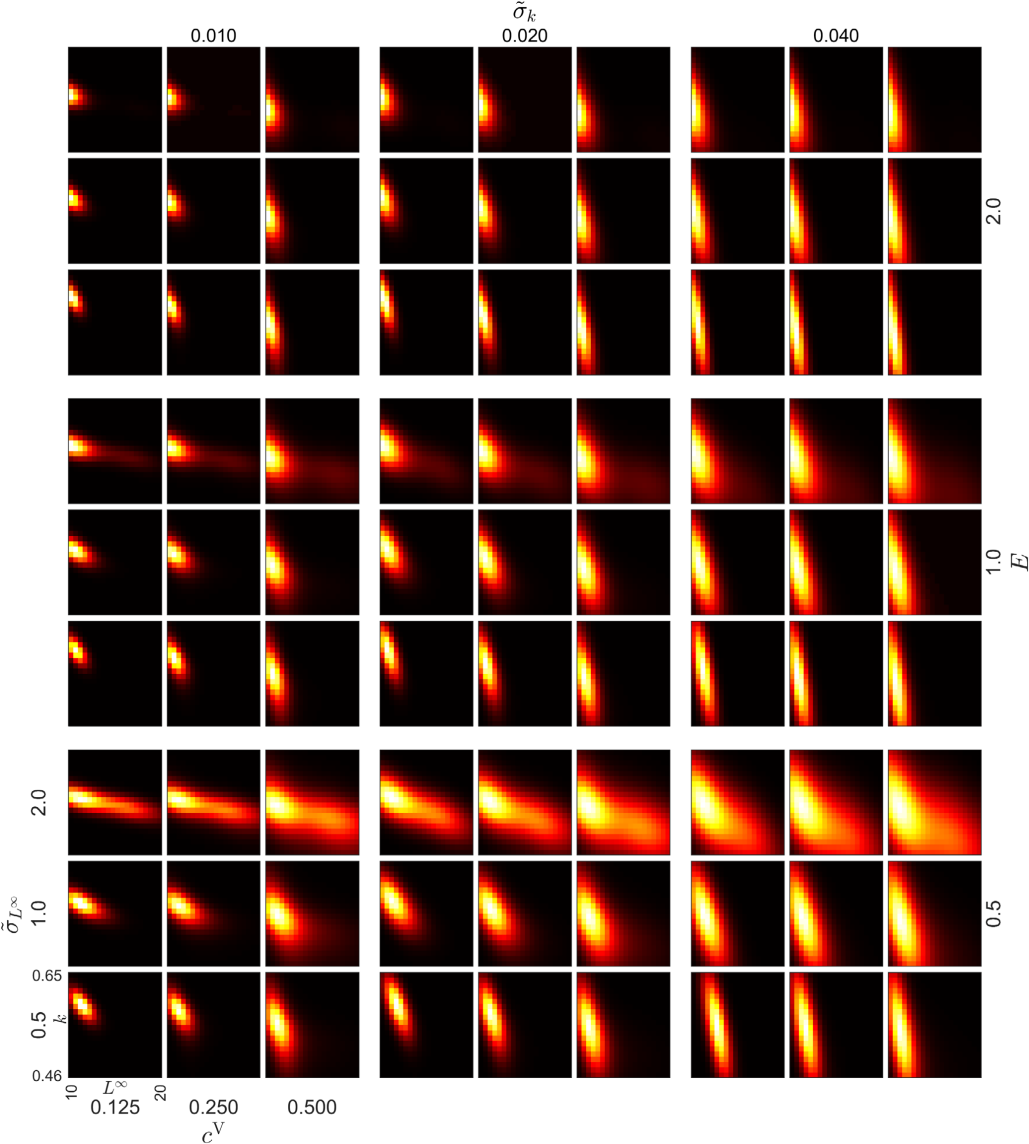
*Coregonus albula* age 4+ trait distribution after 100 years of size‐selective fishing in the simulation, with a high (ρ = −0.7) level of correlation between k (Brody's growth coefficient) and $L^{\infty }$ (asymptotic length). The figure illustrates the influence of varying instantaneous fishing mortalities $\left(E\right)$, and genotypic $\left(c^{V}\right)$ and phenotypic $\left({\tilde{\sigma }}_{L^{\infty }},{\tilde{\sigma }}_{k}\right)$ variance parameters on the eco‐evolutionary impacts of size‐selective fishing. The horizontal axis ($L^{\infty }$) represents the asymptotic length (cm), and the vertical axis (k) represents Brody's growth coefficient ($y^{-1}$). Brighter colours indicate higher trait density. Mean of the starting distribution is in the middle of the grid.

For *C. albula*, evolution was consistently directional towards small $L^{\infty }$ values in all cases (Figures 5 and S21, S22). When ${\tilde{\sigma }}_{L^{\infty }}$ was 2.0 cm, and instantaneous fishing mortality $E=0.5\;y^{-1}$, disruptive selection in $L^{\infty }$ was observed (Figure 5). Evolution in k trended towards intermediate values in all cases (Figure 5).

### 3.2 Variance and heritability of the traits in the populations

**FIGURE 6:**
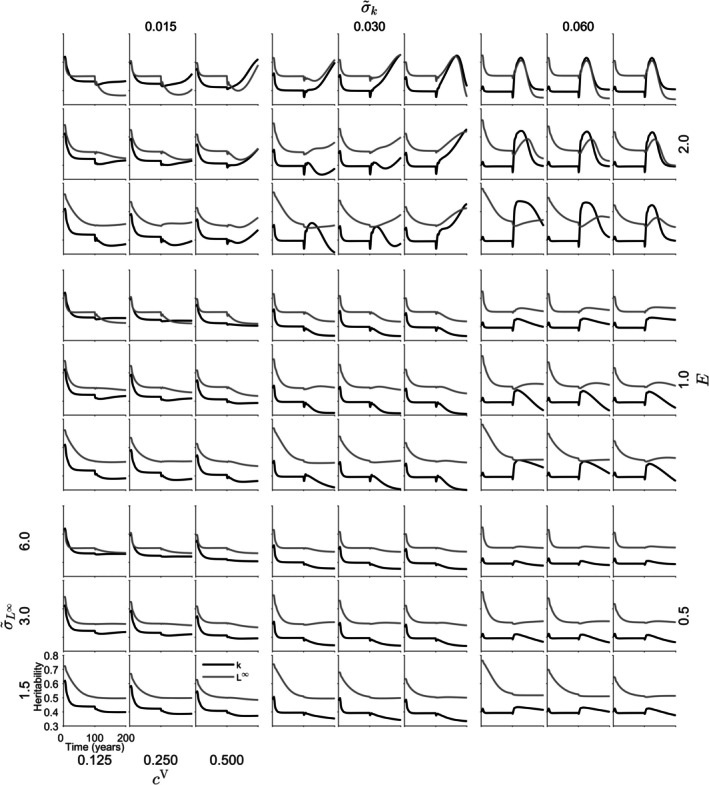
Heritability of *Sander lucioperca* traits; asymptotic length ($L^{\infty }$; grey) and Brody's growth coefficient (k; black) over a 200‐year simulation, with a high (ρ = −0.7) level of correlation between k and $L^{\infty }$. The figure illustrates the influence of varying instantaneous fishing mortalities $\left(E\right)$, and genotypic $\left(c^{V}\right)$ and phenotypic $\left({\tilde{\sigma }}_{L^{\infty }},{\tilde{\sigma }}_{k}\right)$ variance parameters on the heritability. Fishing activities commence at year 101. The horizontal axis represents time, whereas the vertical axis depicts heritability values. The trends in heritability reflect the dynamic evolution of the depicted traits during the simulation.

The heritability of $L^{\infty }$ in *S. lucioperca* ranged from 0.3377 to 0.7623. The heritability of k ranged from 0.3007 to 0.6739. $L^{\infty }$ exhibited higher heritability than k. Fishing onset induced changes in heritability. Under the highest instantaneous fishing mortality ($E=2.0\;y^{-1}$), when ${\tilde{\sigma }}_{k}$ was 0.03 $y^{-1}$ or 0.06 $y^{-1}$, fishing initially increased heritability, often followed by a subsequent decrease (Figure 6). In some cases, when ${\tilde{\sigma }}_{k}$ was 0.03 $y^{-1}$, the increase continued steadily following the onset of fishing (Figure 6). In scenarios where ${\tilde{\sigma }}_{k}$ was 0.015 $y^{-1}$ and instantaneous fishing mortality was high, heritability generally increased; however, in some cases, it subsequently decreased (Figure 6). Smaller instantaneous fishing mortalities also led to changes in heritability towards higher or lower levels (Figure 6). Both variance and heritability exhibited variability across different parameter combinations (Figures 6 and S23, S24, S27–S29). Lower fishing mortalities were associated with more stable variances. In scenarios involving high instantaneous fishing mortality, a pattern of initial variance increase at the onset of fishing followed by a subsequent decrease was frequently observed, particularly in the variance of $L^{\infty }$. Under high fishing mortality, both increasing and decreasing variances were observed after the onset of fishing (Figure S27). Decreasing variances were also noted with lower instantaneous fishing mortality, especially when ${\tilde{\sigma }}_{k}$ was 0.015 $y^{-1}$ or 0.03 $y^{-1}$ (Figure S27). Variance of k tended to be more stable than that of $L^{\infty }.$

**FIGURE 7:**
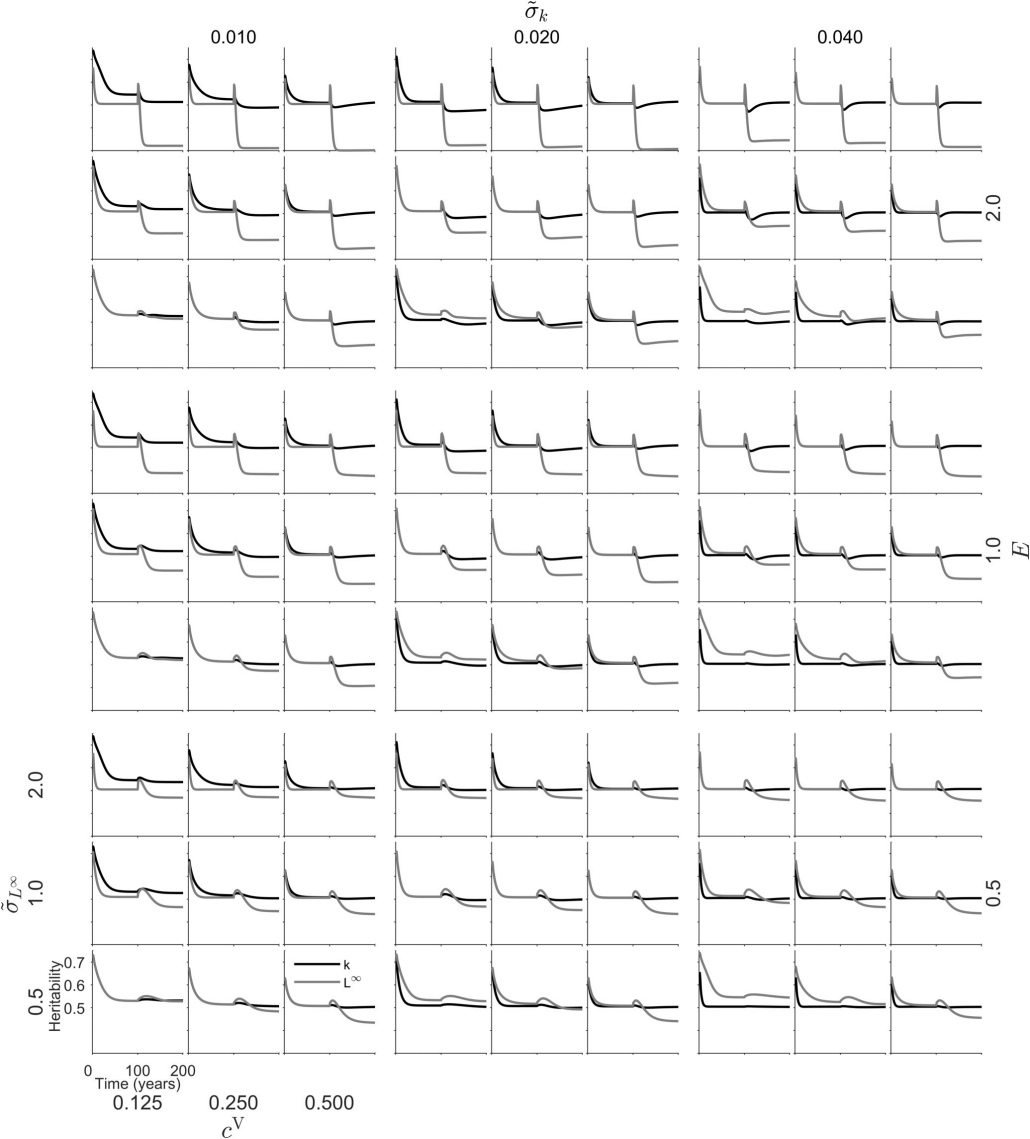
Heritability of *Coregonus albula* traits; asymptotic length ($L^{\infty }$; grey) and Brody's growth coefficient (k; black) over a 200‐year simulation, with a high (ρ = −0.7) level of correlation between k and $L^{\infty }$. The figure illustrates the influence of varying instantaneous fishing mortalities $\left(E\right)$, and genotypic $\left(c^{V}\right)$ and phenotypic $\left({\tilde{\sigma }}_{L^{\infty }},{\tilde{\sigma }}_{k}\right)$ variance parameters on the heritability. Fishing activities commence at year 101. The horizontal axis represents time, whereas the vertical axis depicts heritability values. The trends in heritability reflect the dynamic evolution of the depicted traits during the simulation.

The heritability of $L^{\infty }$ in *C. albula* ranged from 0.3009 to 0.7394. The heritability of k ranged from 0.4711 to 0.7378. Fishing did not cause clear change in the heritability of k, whereas the heritability of $L^{\infty }$ decreased in the presence of fishing (Figure 7). For *C. albula*, variance in k remained relatively stable, while fishing decreased the variance in $L^{\infty }$ (Figure S30). Small decreases in variances of k were also observed at the onset of fishing (Figure S30).

### 3.3 Effects of instantaneous fishing mortality on biomasses in the food web

**FIGURE 8:**
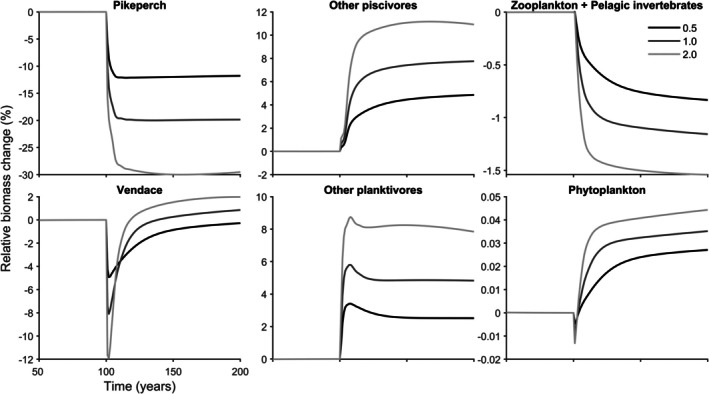
Relative biomass change (%) in various ecological components over a range of instantaneous fishing mortalities ($E=0.5\;y^{-1},1.0\;y^{-1},\text{and}\;2.0\;y^{-1}$), with a high (ρ = −0.7) level of correlation between k (Brody's growth coefficient) and $L^{\infty }$ (asymptotic length) for *Sander lucioperca* and *Coregonus albula*. The components include *S. lucioperca*, other piscivores (*Salmo trutta* and *Perca fluviatilis* age ≥4), *C. albula*, other planktivores (*Perca fluviatilis* age <4, *Coregonus lavaretus* and *Osmerus eperlanus*), zooplankton and pelagic invertebrates, and phytoplankton. The comparison is made between the unfished equilibrium situation and the situation with fishing.

In the model, *S*. *lucioperca* and *C. albula* shared the same instantaneous fishing mortalities ($E=0.5\;y^{-1},$ $E=1.0\;y^{-1}$ or $E=2.0\;y^{-1}$), leading to combined effects on the biomasses of the species in the food web. Biomasses were assessed relative to the unfished equilibrium. The most prominent impacts were negative, directly affecting *S*. *lucioperca* (Figure 8). *S. lucioperca* exhibited the largest biomass at $=0.5\;y^{-1}$, followed by $E=1.0\;y^{-1}$ and the smallest at $E=2.0\;y^{-1}$ (Figure 8). *C. albula* reached its largest biomass at $E=2.0\;y^{-1}$, followed by $E=1.0\;y^{-1}$ and smallest at $E=0.5\;y^{-1}$ (Figure 8). The fishing of *S*. *lucioperca* and *C. albula* had positive repercussions on other planktivores and piscivores. Like *C. albula*, other planktivores and piscivores demonstrated the largest biomass at $E=2.0\;y^{-1}$, followed by $E=1.0\;y^{-1}$ and smallest at $E=0.5\;y^{-1}$ (Figure 8). Minor effects of fishing were observed in the lower trophic levels of the food web, generally manifesting as negative impacts on zooplankton and pelagic invertebrates and positive effects on phytoplankton (Figure 8). Zooplankton and pelagic invertebrates had the largest biomass at $E=0.5\;y^{-1}$, followed by $E=1.0\;y^{-1}$ and the smallest at $E=2.0\;y^{-1}$ (Figure 8). Phytoplankton reached its highest biomass at $E=2.0\;y^{-1}$, followed by $E=1.0\;y^{-1}$, and the lowest at $E=0.5\;y^{-1}$ (Figure 8). Detailed, guild‐specific results can be found in the Appendix (Figure S35–S58).

### 3.4 Sensitivity of the model to the level of correlation

The model was, in general, sensitive to the level of correlation. For example, trait distribution of *S. lucioperca* was affected by the level of correlation (Figures 4 and S19, S20). Under high instantaneous fishing mortality ($E=2.0\;y^{-1}$) and low (ρ = −0.35) or absent (ρ = 0) correlation, the selection trend favoured smaller k and $L^{\infty }$ values (Figures S19 and S20). Instead, *C*. *albula* trait distributions were similar as in the case of high negative correlation (Figures 5 and S21, S22). There were both similarities and differences in the trajectories of the heritability (Figures 6 and S23, S24) and variance (Figures S27–S29) in *S. lucioperca* as well as in the trajectories of the heritability (Figures 7 and S25, S26) and variance (Figures S30–S32) in *C. albula*. In both species, heritability was generally higher in the absence of correlation (ρ = 0). Level of correlation also influenced the biomasses in the food web (Figures 8 and S33–S58).

## 4 DISCUSSION

The potential trajectories of fishing‐induced evolution are intricate and contingent upon the intensity of fishing, level of correlation between asymptotic length and Brody's growth coefficient, and the parameters influencing the levels of genotypic and phenotypic variation. To avoid being caught by normally distributed selection curve, there are two options: either stay below the selection curve or grow quickly above the selection window. In general, fishing induced evolution in *C. albula* was towards small asymptotic length and intermediate Brody's growth coefficient. In contrast, fishing‐induced evolution in *S. lucioperca* was mainly towards larger asymptotic length and Brody's growth coefficient. Disruptive selection was also frequently observed in *S*. *lucioperca* evolution. Additionally, there were cases of small asymptotic length and slow growth especially when correlation was low or absent and fishing mortality was high. These distinctions likely arise from the nuanced selection pressures imposed by gillnetting on *S*. *lucioperca* and trawling on *C. albula*. Additionally, in this complex model, food web interactions and model parameters, such as metabolic rate, can influence the results. Food web–level effects were small in the level of phytoplankton and zooplankton as in the previous study by Kokkonen et al. (2024). In the previous study no cascading effects on the level of phytoplankton were observed (Kokkonen et al., 2024). In contrast, this study observed a trophic cascade (Carpenter et al., 1985), where the largest abundance of *S. lucioperca* resulted in a high abundance of zooplankton and pelagic invertebrates and a corresponding decrease in phytoplankton abundance. Larger effects were seen in the fish species level, and more so in the age guild compositions of fish species.

Intriguingly, many scenarios for *S*. *lucioperca* hinted at evolution favouring larger asymptotic length and faster growth, potentially involving disruptive selection that sustains diversity in the *S*. *lucioperca* trait distribution. Matsumura and others (Matsumura et al., 2011) modelled the impact of having both minimum and maximum size limits, that is, harvest slots in recreational fishing of pike *Esox lucius* L. 1758, Esocidae. This also resulted in positive selection on growth capacity and larger size at age of 4 years. Using harvest slots that target intermediate‐sized fish in fishery management can help preserve the natural age structure and reproductive potential of older fish, while also increasing the number of fish caught (Gwinn et al., 2015). Although fish biomass caught is smaller compared to using minimum size limits, this approach can protect the mature fish (Gwinn et al., 2015). In another study, harvest slots were optimal regulation options when balancing between multiple fishery objectives (Ahrens et al., 2020). In Windermere, fishing for *E. lucius* using gillnets led to disruptive selection, which increased variability in size at age and growth rate (Edeline et al., 2009). There, disruptive selection favoured both slow‐ and fast‐growing *E. lucius* (Edeline et al., 2009). Sattar and others (Sattar et al., 2008) found dimorphism in the allocation of resources either to growth or maturation in a hermaphrodite species *Epinephelus fuscoguttatus* Forsskål 1775. Disruptive selection might therefore be the result of part of the population maturing early and allocating part of the resources to reproduction and other part of the population still allocating all resources to growth before later maturation. In our model, we did not account for evolution in the maturation size and age independently of the selected traits, although these traits could also evolve (e.g., Heino et al., 2015). In a modelling study, Landi et al. (2015) also identified the potential for disruptive selection under high harvest pressure. Fishing policies that could lead to disruptive selection included a lack of regulation, fishing only large fish, targeting either small or large fish and fishing only mature fish (Landi et al., 2015). The increased variability caused by disruptive selection could facilitate the population's adaptation to changes in environmental conditions (Landi et al., 2015). Fishing could also be managed in a way that promotes disruptive selection, such as through mesh‐size regulations that protect the largest individuals, combined with moderate fishing mortality (Edeline et al., 2009). The observed variability in our study aligns with findings from other studies, demonstrating the diverse outcomes of evolution under different fishing pressures. For instance, smaller minimum length limits, capturing also immature fish, favoured faster growth, whereas larger limits were associated with slower growth in the eco‐genetic model of Dunlop and others (Dunlop et al., 2009). Similarly, a previous ATNE modelling study revealed that *P. fluviatilis* subjected to a minimum length limit of 15.9 cm evolved towards lower asymptotic length, whereas the evolution trend shifted towards larger asymptotic lengths when 15.9 cm was set as the maximum limit (Perälä & Kuparinen, 2020). Evolution towards smaller size has also been frequently observed in many exploited fish species (Audzijonyte et al., 2013; Devine et al., 2012), which was also observed in this study in *C. albula*.

In our modelling, we assumed negative correlation between asymptotic length and Brody's growth coefficient (ρ = −0.7) as most probable based on literature. In a study of 33,000 fish species the correlation between the natural logarithm of asymptotic length and the natural logarithm of Brody's growth coefficient was −0.585 (Thorson et al., 2017). The study concluded that, on average, there was a 1.24% decrease in the Brody's growth coefficient when asymptotic length increased 1% (Thorson et al., 2017). In another study, Brody's growth coefficient generally exhibited a decreasing curvilinear trend as asymptotic length increased, which was described by either an exponential or power function (Nadon & Ault, 2016). Jensen (1997) derived relationship between asymptotic length and Brody's growth coefficient from the bioenergetic growth equation as $L^{\infty }=Ck^{-h}$ where the value of h was 0.33. We lacked information on the correlation at the level of individual growth in nature, and further studies are needed to explore this.

Heritability of 0.3 is commonly regarded as typical in life‐history traits (Mousseau & Roff, 1987). In a review of Salmonid fish, median heritability for morphological traits was reported as 0.29, for growth and development it was 0.22 and for length‐at‐age, it stood at 0.29 (Carlson & Seamons, 2008). In brook charr, *Salvelinus fontinalis*, heritability of size exhibited variation between 0.44 and 0.50 (Thériault et al., 2007). Although heritabilities in our modelling were generally high, it is crucial to note that these deviated from traditional methods. Our model lacks the ability to track genotypes and phenotypes separately; instead, it utilizes a hybrid approach that combines elements of both. This distinctive feature in our approach to modelling should be considered when interpreting the heritability estimates. Moreover, heritabilities are typically population‐specific and contingent on environmental conditions (Carlson & Seamons, 2008). Consequently, the initiation of fishing altered the environmental conditions in our simulations, inducing changes in heritability and the extent of variance as well. The potential for rapid evolution in growth‐related traits is supported by both modelling and laboratory experiments (Conover & Munch, 2002; Williams & Shertzer, 2015). Higher heritabilities contribute to a swifter evolutionary response to selection pressures (Williams & Shertzer, 2015).

The effects of fishing on *S*. *lucioperca* and *C. albula* biomasses were notably complex. In general, fishing affected the biomass of *S*. *lucioperca* more radically than that of *C. albula*, but the effects depended on the level of instantaneous fishing mortality. Logically, *S*. *lucioperca* biomass was higher at lower levels of instantaneous fishing mortality. In contrast, in the same simulation, *C. albula* biomass was higher, the higher the instantaneous fishing mortality was. This likely reflects the predatory effects of *S. lucioperca*. The predatory effects of *S. lucioperca* on other planktivores and competitive effects on other piscivores were probably also mirrored in the biomasses of both planktivores and piscivores. Compensatory effects, particularly in the younger age guilds, as well as broader food web–level dynamics may also influence the outcomes. For example, the younger age guilds of *C. albula* exhibited their largest biomass at instantaneous fishing mortality $E=2.0\;y^{-1}$, and from age 2 onwards, the highest biomass was observed at instantaneous fishing effort $E=0.5\;y^{-1}$. Consequently, the highest total biomass of *C. albula* at instantaneous fishing mortality $E=2.0\;y^{-1}$ might be attributed to a compensatory effect involving larger biomasses in younger age guilds. Additionally, the reduced predation pressure from *S*. *lucioperca*, which itself had the smallest total biomass when *C. albula* biomass was highest, could further contribute to these complex interactions. Similarly, in the previous study in Lake Oulujärvi, *S*. *lucioperca* negatively affected especially the youngest age guilds of its planktivorous prey species, whereas fishing primarily targeted the older age guilds (Kokkonen et al., 2024). Additionally, the presence of *S. lucioperca* reduced the proportion of other fish species in the fish biomass (Kokkonen et al., 2024).

A prior investigation conducted in the Archipelago Sea concerning *S*. *lucioperca* reported advanced maturation schedules, indicated by a negative trend in the probabilistic maturation reaction norms (Kokkonen et al., 2015). This study (Kokkonen et al., 2015) revealed that fisheries could potentially influence the age and size at which *S*. *lucioperca* reach maturation. Further exploration of *S*. *lucioperca* growth within the Archipelago Sea delved into the intricate interplay of environmental factors. It unveiled a positive correlation with water temperature but a negative association with population density (Saulamo et al., 2020). Additionally, the phenomenon known as the Rosa‐Lee effect, signifying that fisheries tend to capture the faster‐growing individuals first (Lee, 1912), was observed by examining the back‐calculated lengths (Saulamo et al., 2020). The biomass of the *C. albula* population is also susceptible to a multitude of influences, including fishing pressure (Helminen & Sarvala, 2021; Huusko & Hyvärinen, 2005; Sarvala et al., 2020), environmental conditions like water temperature (Helminen & Sarvala, 2021) and trophic state (Helminen & Sarvala, 2021), as well as ecological relationships in the food web, such as competition with other planktivorous fish species (Helminen & Sarvala, 2021) and the abundance of predators (Sarvala et al., 2020). On the individual level, the growth of *C. albula* can be influenced by *C. albula* population density, the availability of zooplankton prey, and an array of environmental, fishing and food web–related factors that impact *C. albula* population density (Sarvala & Helminen, 2021).

Navigating the multifaceted impacts of fishing on the evolution of size‐related traits, the biomasses of targeted fish species and the broader food web poses a considerable challenge for fisheries management. This challenge becomes particularly pressing as efforts are made to transition towards ecosystem‐based fisheries management (Pikitch et al., 2004). The consideration of fisheries‐induced evolution is integral, and this must be undertaken within the broader environmental context in which such evolution occurs (Thambithurai & Kuparinen, 2023). Mitigating severe evolutionary effects in *S*. *lucioperca* may be achievable with smaller fishing mortalities, which not only preserve the largest biomass of *S*. *lucioperca* in the lake but also contribute to the highest abundance of pelagic invertebrates and zooplankton, while minimizing the biomass of phytoplankton. It is essential to recognize that the optimal instantaneous fishing mortalities for *C. albula*, other planktivorous fish and other piscivorous fish may differ, requiring careful prioritization. In the case of *C. albula* trawling, avoiding evolutionary effects on *C. albula* size proves challenging, particularly with tested instantaneous fishing mortalities. In contrast, the biomass of *C. albula* was not seriously affected by the higher instantaneous fishing mortalities and the lower biomasses of *S. lucioperca* allowed for larger biomasses of *C. albula*. In *C. albula* trawling, approximately 50% of *C. albula* that goes through the trawl may die afterwards (Suuronen et al., 1995). This was not considered in our modelling.

It is crucial to acknowledge that our study is theoretical and conducted in a modelling context. The real‐world scenario involves diverse fishing methods, varying fishing mortalities across different species, operating simultaneously. Additionally, we lack knowledge about the specific values of phenotypic and genotypic variance‐describing parameters that accurately represent the real‐world conditions in the reproduction model. Trait distributions in the modelling are also limited by the choice of the grid. It is possible that the traits favour the borders of the grid, when modelling evolution. All results are not in accordance with the expectation of negative correlation between asymptotic length and Brody's growth coefficient, as there were many cases of positive correlation, that is, fast growth and high asymptotic length or slow growth and small asymptotic length. Simulations could also produce also these kinds of results. In nature, slow growth and small asymptotic length could be caused by low availability of resources, and fast growth and large asymptotic length by high availability of resources. For example, in *S. trutta* individuals from weakly recruited cohorts were fast growing and attained larger size (Lobón‐Cerviá, 2022). We did not consider the possibility of *S. lucioperca* and *C. albula* inducing evolution in the food web, that could be a subject for further modelling. Density‐dependent growth could also influence growth patterns; however, it was not included in our model, as we focused on the evolution of growth and effects within the food web. Gillnetting of *S. lucioperca* could lead to many directions of evolution in Brody's growth coefficient and asymptotic length, highlighting the importance of adopting adaptive management strategies (Walters, 1986), capable of responding to observed changes, which also stands out as an optimal approach for ecosystem‐based fisheries management. Gillnetting of *S. lucioperca* also resulted in disruptive selection in many simulations, and that might be a good strategy to maintain diversity in Brody's growth coefficient and asymptotic length. In the model, trawling of *C. albula* led to a decrease in asymptotic length, which reflects well to the current situation of *C. albula* in Lake Oulujärvi, as there has been a decrease in *C. albula* sizes (Härkönen et al., 2023). Fishing‐induced changes towards smaller size in fish have been observed globally (Audzijonyte et al., 2013; Devine et al., 2012), but environmental changes, like increasing water temperatures, can also lead to declines in fish size (Cheung et al., 2013). In Lake Oulujärvi there are environmental changes like warming water temperature, declining phosphorus content and changes in the food web level, which could also affect changes in the fish sizes. In the model, fishing also affected the biomass composition of Lake Oulujärvi food web, and therefore it is important to also follow the ecological consequences of fishing.

## AUTHOR CONTRIBUTIONS

Eevi Kokkonen: Conceptualization, data curation, formal analysis, investigation, validation, visualization, writing – original draft, writing – review and editing, funding acquisition. Tommi Perälä: Conceptualization, formal analysis, investigation, supervision, methodology, software, validation, visualization, writing original draft, writing – review and editing. Laura S. Härkönen: Data curation, formal analysis, supervision, writing – review and editing, funding acquisition. Pekka Hyvärinen: Data curation, formal analysis, supervision, writing – review and editing, funding acquisition. Anna Kuparinen: Conceptualization, investigation, supervision, writing – review and editing, funding acquisition.

## FUNDING INFORMATION

This project received funding from the European Research Council (ERC) under the European Union's Horizon 2020 research and innovation programme (grant agreement COMPLEX‐FISH No 770884 to Anna Kuparinen). The project also received funding from the Academy of Finland (project grant 317495 to Anna Kuparinen). and Natural Resources Institute Finland (project DEFIP to Pekka Hyvärinen and Laura S. Härkönen). In addition, the work received financial support from the UEF Water research program, which is jointly funded by the Saastamoinen Foundation, the Jenny and Antti Wihuri Foundation and the Olvi Foundation (Eevi Kokkonen).

## CONFLICT OF INTEREST STATEMENT

The authors declare that they have no conflicts of interest.

## Supporting information


**Data S1.** Supporting information.
