# Supplementary material for: Modelling fishing‐induced evolution in pikeperch ( Sander lucioperca ) and vendace ( Coregonus albula ), Lake Oulujärvi, as template for ecosystem model
Source: J Fish Biol. 2025 Mar 24;107(1):229–47. doi: 10.1111/jfb.70028 (PMC12327190; doi:10.1111/jfb.70028)
Supplement: Supplementary file 1 — Data S1. Supporting information. [file JFB-107-229-s001.docx]

**Appendix**

**ALLOMETRICALLY SCALED GROWTH RATE OF PRODUCERS**

The intrinsic growth rate of the producers in their logistic growth model was calculated based on the body masses of the producers using the following allometric scaling relationship (Boit et al., 2012; Brose et al., 2006; De Castro & Gaedke, 2008):

$$r_{i}=\left( \frac{M_{\mathrm{ref}}}{M_{i}} \right)^{0.15}, (S1)$$

where $M_{i}$ is the body mass of the producer guild $i$ in micrograms of carbon per individual (Table S1), and $M_{\mathrm{ref}}$ is the body mass of the reference producer guild, “Alg1A”.

**ALLOMETRICALLY SCALED METABOLIC RATE OF CONSUMERS**

The mass-specific metabolic rate of the consumer guild $i$ was calculated based on the body masses of the consumers using the following allometric scaling relationship (Boit et al., 2012; Brose et al., 2006; De Castro & Gaedke, 2008):

$$x_{i}=0.314\cdot\left( \frac{M_{\mathrm{ref}}}{M_{i}} \right)^{0.15}, (S2)$$

where $M_{i}$ is the body mass of the consumer guild $i$ in micrograms of carbon per individual (Table S1).

**ALLOMETRICALLY SCALED DYNAMIC METABOLIC RATE OF FISH**

The time dependent mass-specific metabolic rate $x_{i,g}\left( t \right)$ of $g$th genotype group of fish guild $i$ ($\text{GG}_{i,g}$) of age $a_{i}$ was calculated based on the following allometric scaling relationship (Brose et al., 2006; Killen et al., 2007, 2010):

$$x_{i,g}\left( t \right)=0.88\cdot\left( \frac{M_{\mathrm{ref}}}{M_{i,g}\left( t \right)} \right)^{0.11}, (S3)$$

where $M_{i,g}\left( t \right)$ is the body mass of the fish $\text{GG}_{i,g}$ in micrograms of carbon, which is calculated based on the fish lengths:

$$M_{i,g}\left( t \right)=0.2\cdot0.53\cdot a_{m_{i}}{L_{i,g}\left( t \right)}^{b_{m_{i}}}, (S4)$$

where $L_{i,g}\left( t \right)$ is the time-dependent length of the fish in the genotype group $g$ of fish guild $i$, $a_{m_{i}}$ and $b_{m_{i}}$ are the species-specific parameters of the length-weight relationship (Table S2), and it is assumed that the dry weight of the fish is 0.2 times its fresh weight, and that carbon constitutes 53% of the dry weight (Kuparinen et al., 2016).

The time-dependent length is modelled using the von Bertalanffy equation:

$$L_{i,g}\left( t \right)=L_{i,g}^{\infty}-\left( L_{i,g}^{\infty}-L_{i}^{0} \right)e^{-k_{i,g}\left( a_{i}+\frac{t}{t_{\mathrm{end}}} \right)}, (S5)$$

where $L_{i,g}^{\infty}$ and $k_{i,g}$ are the genotype group specific asymptotic maximum length and growth coefficient of fish guild $i$, respectively, $a_{i}$ is the age in full years of fish guild $i$, $L_{i}^{0}$ is the length of age 0 fish of the fish species to which guild $i$ belongs at $t=0$, $t_{\mathrm{end}}$ is the length of the growth season.

**GAINS**

**Intrinsic growth of producers.** The gain from the *intrinsic growth* of producer guild $i\in\mathcal{I}_{P}$ is modelled using a logistic growth model, i.e.,

$$\mathcal{G}_{i}^{\text{growth}}\left( t \right)=\left( 1-s_{i} \right)r_{i}B_{i}\left( t \right)\left( 1-\frac{1}{K}\sum_{j\in\mathcal{I}_{P}} c_{i,j}B_{j}\left( t \right) \right), (S6)$$

where $s_{i}=0.2$ is the fraction of exudation, $r_{i}$ is the allometrically scaled mass-specific intrinsic growth rate (eqn S1), $c_{i,j}=1$ and $c_{i,i}=2$ are the interspecific and intraspecific producer competition coefficients, respectively, $K=540 000 \mu gCm^{-3}$ is the parameter controlling (but not equal to) the
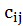
carrying capacity shared by all producer guilds, and $\mathcal{I}_{P}$ denotes the set of indices for the producer guilds.

**Consumption.** The gain for guild $i$ from the *consumption* of its prey guild $j\in\mathcal{I}_{i}^{\text{prey}}$ is

$$\mathcal{G}_{i,j}^{\text{consumption}}\left( t \right)=f_{a}x_{i}B_{i}\left( t \right)y_{i,j}F_{i,j}\left( \boldsymbol{B}\left( t \right) \right), (S7)$$

where $f_{a}=0.4$ ($f_{a}=0.2$ for “Bac”) is the fraction of assimilated biomass which gets converted to the consumers own biomass, $x_{i}$ is the allometrically scaled mass-specific metabolic rate (eqn S2), and $y_{i,j}$ the maximum feeding rate scaling factors of guild $i$ feeding on guild $j$ (for parameters values see Table S4) . We use a hybrid DeAngelis-Beddington – Holling type II.5 functional response:

$$F_{i,j}\left( \boldsymbol{B}\left( t \right) \right)=\frac{\omega_{i,j}{B_{j}\left( t \right)}^{q}}{{B0}_{i,j}^{q}+d_{i,j}{B0}_{i,j}^{q}B_{i}\left( t \right)+\sum_{k\in\mathcal{I}_{i}^{\text{prey}}} \omega_{i,k}{B_{k}\left( t \right)}^{q}}, (S8)$$

where $\omega_{i,j}=1/N_{i}$ is the consumer’s relative resource preference and $N_{i}$ is the number of its resource guilds, $q=1.2$ is the form factor of the functional response shown to stabilize food web dynamics using the Holling type functional response (Williams & Martinez, 2004), ${B0}_{i,j}$ is the half saturation constant describing the biomass of the prey at which the predator achieves half of its maximum feeding rate when consuming only prey $j$ and in the absence of feeding interference, and $d_{i,j}$ is the coefficient of intraspecific feeding interference. The parameter values for ${B0}_{i,j}$ and $d_{i,j}$ are shown in Table S4.

The fish guilds have time-varying mass-specific metabolic rates, and the genotype group structure (for fish species other than pikeperch *Sander lucioperca* L. 1758 and vendace *Coregonus albula* L. 1758, there is only one genotype group, i.e., $g=1$). The consumption gains are calculated for each $\text{GG}_{i,g}$ separately as

$$\mathcal{G}_{i,g,j}^{\text{consumption}}\left( t \right)=f_{a}x_{i,g}\left( t \right)B_{i,g}\left( t \right)y_{i,j}F_{i,j}\left( \boldsymbol{B}\left( t \right) \right), (S9)$$

where $x_{i,g}\left( t \right)$ is the mass-specific dynamic metabolic rate (eqn S3).

**LOSSES**

**Maintenance of bodily functions.** The rate of biomass loss due to *maintenance* of bodily functions is

$$\mathcal{L}_{i}^{\text{maintenance}}\left( t \right)=f_{m}x_{i}B_{i}\left( t \right), (S10)$$

where $f_{m}=0.1$ is the maintenance respiration coefficient. This parameter is increased by 100% for the mature individuals to create the trade-off between current and future reproduction, i.e., the survival cost of reproduction (Bell, 1980). For $\text{GG}_{i,g}$,

$$\mathcal{L}_{i,g}^{\text{maintenance}}\left( t \right)=f_{m}x_{i,g}\left( t \right)B_{i,g}\left( t \right). (S11)$$

**Consumption.** The rate of biomass loss due to *consumption* of guild $i$ by its consumer guild $j$ is

$$\mathcal{L}_{i,j}^{\text{consumption}}\left( t \right)=\frac{1}{f_{a}e_{j,i}}\mathcal{G}_{j,i}^{\text{consumption}}\left( t \right), (S12)$$

where $e_{j,i}$ is the assimilation efficiency describing the fraction of ingested biomass not lost by egestion (for parameter values see Table S4). For $\text{GG}_{i,g}$ we distribute the total loss of guild $i$ based on the distribution of the guild biomass and induce greater losses for smaller individuals.

$$\mathcal{L}_{i,g,j}^{\text{consumption}}\left( t \right)=\pi_{i,g}\left( t \right)\mathcal{L}_{i,j}^{\text{consumption}}\left( t \right), (S13)$$

where the fraction of the total loss assigned to $\text{GG}_{i,g}$ is proportional to its biomass and suitably chosen function of its length

$$\pi_{i,g}\left( t \right)\propto B_{i,g}\left( t \right)\cdot{L_{i,g}\left( t \right)}^{-\frac{1}{b_{m_{i}}}}. (S14)$$

**Fishing**. The loss for $\text{GG}_{i,g}$ caused by fishing using a size selective fishing gear is modelled as

$$\mathcal{L}_{i,g}^{\text{fishing}}\left( t \right)=h_{i,g}\left( t \right)B_{i,g}\left( t \right), (S15)$$

where $h_{i,g}\left( t \right)=ES_{i,g}\left( t \right)$ is the instantaneous daily fishing mortality of genotype group $g$ of guild $i$ at time $t$ when the instantaneous daily fishing mortality is $E$ and it is obtained from the instantaneous yearly fishing mortality by dividing by the length of the growth season, and the selectivity of the fishing gear for genotype group $g$ of guild $i$ at time t is $S_{i,g}\left( t \right)$.

*S. lucioperca* was fished with a gillnet with a knot size 50 mm targeting age classes of 1 and older, whereas *C. albula* was fished with a trawl targeting all age classes. For both fishing gear the selectivity curve was bell-shaped and of the form

$$S_{i,g}\left( t \right)=e^{-\frac{\left( L_{i,g}\left( t \right)-\mu\right)^{2}}{2\sigma^{2}}} (S16)$$

For the gillnet $\mu=46.72 \mathrm{cm}$ and $\sigma=3.44 \mathrm{cm}$ and for the trawl $\mu=12.21 \mathrm{cm}$ and $\sigma=1.71 \mathrm{cm}$. The origins of these values are described in the subsection “Modelling fishing gears and their selectivity” of the Methods section.

**Fish reproduction.** The loss for $\text{GG}_{i,g}$ caused by allocating biomass for *reproduction* is

$$\mathcal{L}_{i,g}^{\text{reproduction}}\left( t \right)=\left\{ \begin{matrix} P_{i,g}\left( t \right)I_{i}\cdot\frac{{\mathcal{G}_{i,g}\left( t \right)}^{2}}{2\mathcal{L}_{i,g}\left( t \right)}, & \mathcal{G}_{i,g}\left( t \right)<\mathcal{L}_{i,g}\left( t \right) \\ P_{i,g}\left( t \right)I_{i}\cdot\left( \mathcal{G}_{i,g}\left( t \right)-\frac{1}{2}\mathcal{L}_{i,g}\left( t \right) \right), & \mathcal{G}_{i,g}\left( t \right)\geq\mathcal{L}_{i,g}\left( t \right) \end{matrix} \right. (S17)$$

where $P_{i,g}\left( t \right)$ denotes the proportion of mature biomass in $\text{GG}_{i,g}$ at time $t$ and is modelled using the following maturity ogive:

$$P_{i,g}\left( t \right)=\frac{1}{1+e^{\text{log}\left( \frac{1-p}{p} \right)\frac{L_{i,g}^{50\%}-L_{i,g}\left( t \right)}{L_{i,g}^{50\%}-L_{i,g}^{p}}}}, (S18)$$

where $L_{i,g}^{50\%}$ is the length at which half of the biomass in $\text{GG}_{i,g}$ is mature, $L_{i,g}^{p}$ is the length at which the fraction of mature biomass is $0<p<1$ (Perälä & Kuparinen, 2020). In our model, we set $p=0.05$ and link these two parameters to the asymptotic maximum length $L_{i,g}^{\infty}$ as $L_{i,g}^{50\%}=\frac{2}{3}L_{i,g}^{\infty}$ and $L_{i,g}^{5\%}=\frac{1}{2}L_{i,g}^{\infty}$ (Perälä & Kuparinen, 2020). The strength of the reproductive investment $I_{i}$ depends on the fish species and the age class (Table S3). The piecewise defined model of reproduction (eqn S17) is used to enforce impaired reproduction when the maintenance losses are greater than the consumption gains. The reproduction losses depend on the total gain for fish $\text{GG}_{i,g}$ which is the sum of the consumption gains over all of its prey species

$$\mathcal{G}_{i,g}\left( t \right)=\sum_{j\in\mathcal{I}_{i}^{\text{prey}}} \mathcal{G}_{i,g,j}^{\text{consumption}}\left( t \right) (S19)$$

and on the total losses without the reproduction which is the sum of maintenance loss, fishing loss, and consumption losses to each predator

$$\mathcal{L}_{i,g}\left( t \right)=\mathcal{L}_{i,g}^{\text{maintenance}}\left( t \right)+\left( \sum_{k\in\mathcal{I}_{i}^{\text{predators}}} \mathcal{L}_{i,g,k}^{\text{consumption}}\left( t \right) \right)+\mathcal{L}_{i,g}^{\text{fishing}}\left( t \right). (S20)$$

**References**

Bell, G. (1980). The Costs of Reproduction and Their Consequences. *The American Naturalist*, *116*(1), 45–76. https://doi.org/10.1086/283611

Boit, A., Martinez, N. D., Williams, R. J., & Gaedke, U. (2012). Mechanistic theory and modelling of complex food-web dynamics in Lake Constance. *Ecology Letters*, *15*(6), 594–602. https://doi.org/10.1111/j.1461-0248.2012.01777.x

Brose, U., Williams, R. J., & Martinez, N. D. (2006). Allometric scaling enhances stability in complex food webs. *Ecology Letters*, *9*(11), 1228–1236. https://doi.org/10.1111/j.1461-0248.2006.00978.x

De Castro, F., & Gaedke, U. (2008). The metabolism of lake plankton does not support the metabolic theory of ecology. *Oikos*, *117*(8), 1218–1226. https://doi.org/10.1111/j.0030-1299.2008.16547.x

Killen, S. S., Atkinson, D., & Glazier, D. S. (2010). The intraspecific scaling of metabolic rate with body mass in fishes depends on lifestyle and temperature. *Ecology Letters*, *13*(2), 184–193. https://doi.org/10.1111/j.1461-0248.2009.01415.x

Killen, S. S., Costa, I., Brown, J. A., & Gamperl, A. K. (2007). Little left in the tank: Metabolic scaling in marine teleosts and its implications for aerobic scope. *Proceedings of the Royal Society B: Biological Sciences*, *274*(1608), 431–438. https://doi.org/10.1098/rspb.2006.3741

Kuparinen, A., Boit, A., Valdovinos, F. S., Lassaux, H., & Martinez, N. D. (2016). Fishing-induced life-history changes degrade and destabilize harvested ecosystems. *Scientific Reports*, *6*(1), 1–9. https://doi.org/10.1038/srep22245

Perälä, T., & Kuparinen, A. (2020). Eco-evolutionary dynamics driven by fishing: From single species models to dynamic evolution within complex food webs. *Evolutionary Applications*, *13*(10), 2507–2520. https://doi.org/10.1111/eva.13058

Williams, R. J., & Martinez, N. D. (2004). Stabilization of chaotic and non-permanent food-web dynamics. *The European Physical Journal B*, *38*(2), 297–303. https://doi.org/10.1140/epjb/e2004-00122-1

**Tables**

**Table S1** Body masses of producer and consumer guilds in the model from Kokkonen et al., 2024.

| Label | Bodymass $(\mu gC/\mathrm{indiv})$ |
| --- | --- |
| Alg1A | 0.000104 |
| Alg1B | 0.000227 |
| Alg2 | 0.00121 |
| Alg3 | 1.41e-05 |
| Alg4 | 0.000109 |
| Alg5 | 2.2e-05 |
| APP | 2.5e-07 |
| Bac | 1.56e-08 |
| HNF | 8e-06 |
| Cil1 | 0.000256 |
| Cil2 | 0.00205 |
| Cil3 | 0.0041 |
| Cil4 | 0.00819 |
| Cil5 | 0.0655 |
| Rot1 | 0.059 |
| Rot2 | 0.0638 |
| Rot3 | 0.0976 |
| Asp | 0.757 |
| Cru | 0.9504 |
| Cyc | 0.8471 |
| Lep | 8.9679 |
| Mys | 3500 |
| Cha | 18.92 |

**Table S2** Parameters of the length-weight relationship (eqn S2) for the fish species in the model from Kokkonen et al., 2024.

| Species | $a_{m}$ | $b_{m}$ |
| --- | --- | --- |
| *Coregonus lavaretus* | 0.002692 | 3.332 |
| *Perca fluviatilis* | 0.0054442 | 3.3 |
| *Osmerus eperlanus* | 0.006006 | 2.972 |
| *Coregonus albula* | 0.005743 | 3.076 |
| *Sander lucioperca* | 0.002736 | 3.328 |
| *Salmo trutta* | 0.0026768 | 3.377 |

**Table S3** The strength of reproductive investment for the different fish age classes in the model from Kokkonen et al., 2024. *Salmo trutta* was differentially formatted than in the previous article, i.e., lake age was not used this time.

| **Species \**  **Age class** | **0** | **1** | **2** | **3** | **4/4+** | **5/5+** | **6+** |
| --- | --- | --- | --- | --- | --- | --- | --- |
| *Coregonus lavaretus* | 0 | 0 | 0.1 | 0.15 | 0.2 | 0.2 | 0.2 |
| *Perca fluviatilis* | 0 | 0 | 0.1 | 0.15 | 0.2 | 0.2 | 0.2 |
| *Osmerus eperlanus* | 0 | 0 | 0.1 | 0.15 | 0.2 | 0.2 | 0.2 |
| *Coregonus albula* | 0 | 0.2 | 0.2 | 0.2 | 0.20 | - | - |
| *Sander lucioperca* | 0 | 0 | 0 | 0 | 0.15 | 0.15 | 0.2 |
| *Salmo trutta* | - | - | 0 | 0 | 0.15 | 0.15 | - |

**Table S4.** Model parameters, $B0$ = half saturation density µgC m^-3^, $y$ = maximum feeding rate scaling factor, $d$ = intraspecific feeding inference m^3^ ugC^-1^, $e$ = assimilation efficiency. On parameter values see Brose et al. (2006) and Bland et al. (2019).

| **Feeding** | $B0$ | $y$ | $d$ | $e$ |
| --- | --- | --- | --- | --- |
| Bacteria | 150,000 | 8 | 0.01 | 0.45 |
| HNF eating APP | 1,500 | 8 | 0.01 | 0.45 |
| HNF eating bacteria | 1,500 | 8 | 0.01 | 0.85 |
| Zooplankton eating phytoplankton | 1,500 | 8 | 0.01 | 0.45 |
| Zooplankton eating other than phytoplankton | 1,500 | 8 | 0.01 | 0.85 |
| Pelagic invertebrates eating phytoplankton | 1,500 | 8 | 0.01 | 0.45 |
| Pelagic invertebrates eating other than phytoplankton | 1,500 | 8 | 0.01 | 0.85 |
| Age 0 fish eating zooplankton | 1,500 | 8 | 0.01 | 0.85 |
| Age 0 fish eating pelagic invertebrates | 1,500 | 8 | 0.01 | 0.85 |
| Age 0 fish eating fish | 15,000 | 8 | 0.0003 | 0.85 |
| Age > 0 fish eating zooplankton | 50,000 | 4 | 0.0001 | 0.85 |
| Age > 0 fish eating pelagic invertebrates | 50,000 | 4 | 0.0001 | 0.85 |
| Age > 0 fish eating fish | 15,000 | 4 | 0.0003 | 0.85 |

**Bayesian models of Von Bertalanffy growth for the Lake Oulujärvi ATNE model fish species**

**Specific descriptions**

***Sander lucioperca***

There were 10887 *S. lucioperca* individuals captured, whose length and age were measured, from Lake Oulujärvi during years 1973–1975, 1988–1990, and 1992–2018. From 600 pond-reared larvae *S. lucioperca* (reared in Kainuu Fisheries Research Station), whose parents originated from Lake Oulujärvi, length had been measured on 17^th^ July 2019 and still from 27 larvae in October 2019. They were given estimated birth date of June 28 as the exact birth date was not known. These were added to growth data to include more age-0 fish. To include more older fish (ages 3–17, mean age 11.95) 40 fish caught with fyke nets from Lake Oulujärvi in year 2018 and kept in an artificial pond (date of captured from the pond approximately 12^th^ April 2022) were also added.

For *S. lucioperca* Bayesian von Bertalanffy model results were used to help determine the lower and upper limits for $L^{\infty}$ and $k$ parameters (**Figure S1**). Prior parameters used were $L_{\mathrm{low}}^{\infty}$ = 70 cm, $L_{\mathrm{up}}^{\infty}$ = 120 cm, $k_{\mathrm{low}}$ = 0.01 $y^{-1}$, $k_{\mathrm{up}}$ = 0.7 $y^{-1}$, $L_{\mathrm{low}}^{0}$= 0.01 cm, $L_{\mathrm{up}}^{0}$ = 10 cm, $\sigma_{\mathrm{low}}$ = 0.05 and $\sigma_{\mathrm{up}}$ = 0.3. The prediction of the model agreed with the data quite well (**Figure S2**).

For the ATNE model these parameter values were used $L_{\mathrm{low}}^{\infty}$ = 70 cm, $L_{\mathrm{up}}^{\infty}$ = 90 cm, $k_{\mathrm{low}}$ = 0.1 $y^{-1}$ and $k_{\mathrm{up}}$ = 0.2 $y^{-1}$. $L^{0}$ was taken from the mean $L^{0}$ to be 1.7 cm.


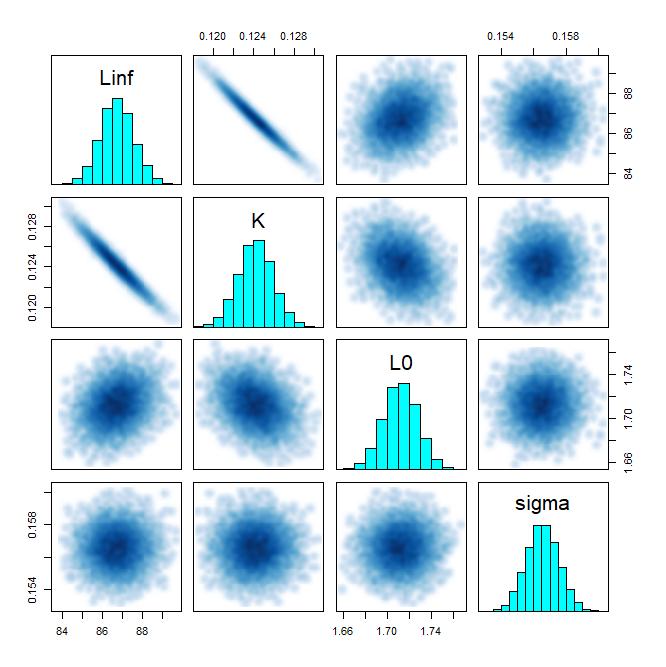


**Figure S1.** Bayesian model results for *Sander lucioperca* showing the histograms for each parameter, ($L^{\infty}$= length at infinity, $k$= Brody´s growth coefficient, $L^{0}$ = length at birth, sigma = variance) and their correlations with each other (with prior parameters: $L_{\mathrm{low}}^{\infty}$ = 70 cm, $L_{\mathrm{up}}^{\infty}$ = 120 cm, $k_{\mathrm{low}}$ = 0.01 $y^{-1}$, $k_{\mathrm{up}}$ = 0.7 $y^{-1}$, $L_{\mathrm{low}}^{0}$ = 0.01 cm, $L_{\mathrm{up}}^{0}$ = 10 cm, $\sigma_{\mathrm{low}}$ = 0.05 and $\sigma_{\mathrm{up}}$ = 0.3).


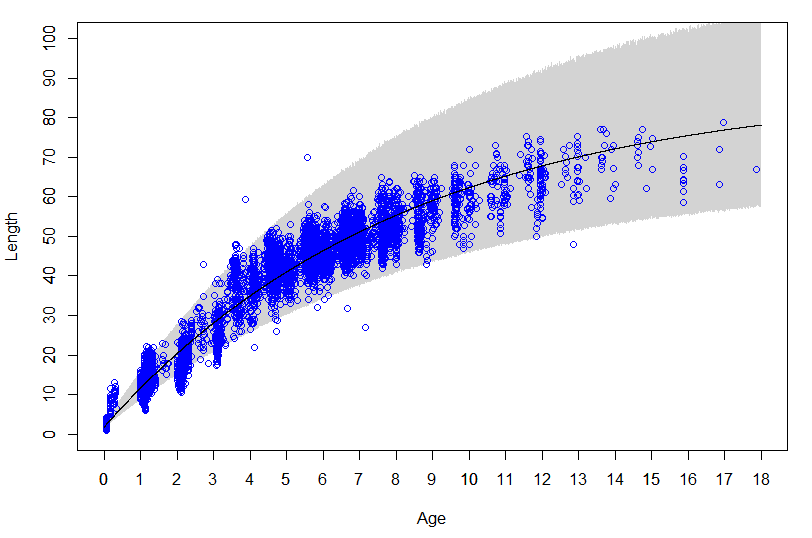


**Figure S2.** Length of *Sander lucioperca* in y-axis at different ages in x-axis, data points in blue, length-at-age predicted from the Bayesian von Bertalanffy prediction in black line, and credible intervals 0.025–0.975 in grey.

***Coregonus albula***

There were 20026 *C. albula* individuals captured, whose length and age were measured, from Lake Oulujärvi during years 1973–1984 and 1986–2017.

As prior parameters for Bayesian von Bertalanffy model we set a) $L_{\mathrm{low}}^{\infty}$ = 10 cm, $L_{\mathrm{up}}^{\infty}$ = 30 cm, $k_{\mathrm{low}}$ = 0.05 $y^{-1}$, $k_{\mathrm{up}}$ = 0.7 $y^{-1}$, $L_{\mathrm{low}}^{0}$ = 0.01 cm, $L_{\mathrm{up}}^{0}$ = 3 cm, $\sigma_{\mathrm{low}}$ = 0.05, $\sigma_{\mathrm{up}}$ = 0.3 and as less restricted version b) we had prior parameters chosen as $L_{\mathrm{low}}^{\infty}$ = 10 cm, $L_{\mathrm{up}}^{\infty}$ = 30 cm, $k_{\mathrm{low}}$ = 0.05 $y^{-1}$, $k_{\mathrm{up}}$ = 0.9 $y^{-1}$, $L_{\mathrm{low}}^{0}$ = 0.3 cm, $L_{\mathrm{up}}^{0}$ = 15 cm, $\sigma_{\mathrm{low}}$ = 0.05 and $\sigma_{\mathrm{up}}$ = 0.8. The prior option a) (**Figure S3**) restricted the histogram distributions of $L^{0}$ and $k$, while option b) did not (**Figure S4**). The predictions from the Bayesian models are shown in **figures** **S5** (with a) prior parameters) and **S6** (with b) prior parameters). Bayesian modelling produced some very narrow posterior distribution so we did not use these in the ATNE model directly as then there would not have been much room for the evolution to happen in the model. In addition, without restricting $L^{0}$ it got to a quite high level.

For the ATNE model $L_{\mathrm{low}}^{\infty}$ was set to 10 cm and $L_{\mathrm{up}}^{\infty}$ to 20 cm, $k_{\mathrm{low}}$ was 0.45 $y^{-1}$, and $k_{\mathrm{up}}$ was 0.65 $y^{-1}$. In the literature, *C. albula* length few weeks after hatching was $E\left( L_{\mathrm{ven}}^{0+} \right)$ = 1.1 cm with standard deviation $\mathrm{SD}\left( L_{\mathrm{ven}}^{0+} \right)$ = 0.21 cm (Sutela & Huusko, 2000) and in another study on May 21^st^ the length of *C. albula* larvae was $E\left( L_{\mathrm{ven}}^{0+} \right)$ = 1.01 cm with standard deviation $\mathrm{SD}\left( L_{\mathrm{ven}}^{0+} \right)$ = 0.06 cm (Sutela & Huusko, 1997). We used $L_{\mathrm{ven}}^{0}=E\left( L_{\mathrm{ven}}^{0+} \right)-\mathrm{SD}\left( L_{\mathrm{ven}}^{0+} \right)=$0.9 cm for ATNE model calculated from the literature information Sutela & Huusko 2000 that was smaller value than from Sutela & Huusko, 1997.


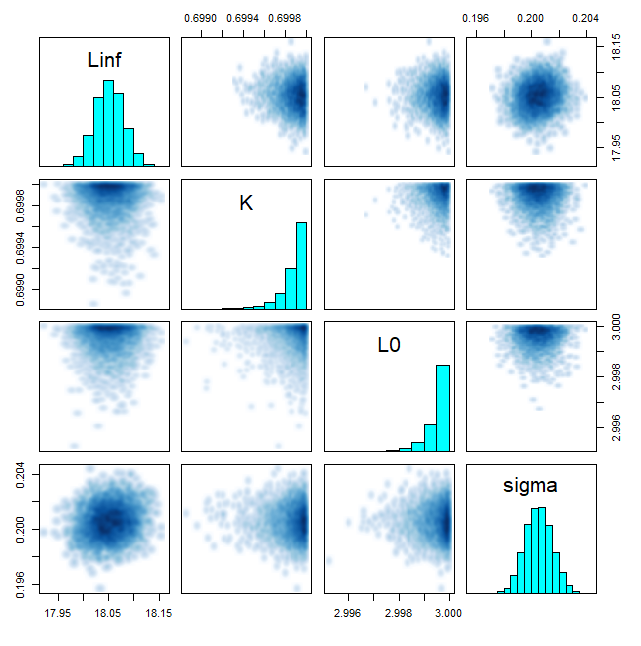


**Figure S3.** Bayesian model results for *Coregonus albula* showing the histograms for each parameter, ($L^{\infty}$ = length at infinity, $k$ = Brody´s growth coefficient, $L^{0}$ = length at birth, sigma = variance) and their correlations with each other (with prior parameters: $L_{\mathrm{low}}^{\infty}$ = 10 cm, $L_{\mathrm{up}}^{\infty}$ = 30 cm, $k_{\mathrm{low}}$ = 0.05 $y^{-1}$, $k_{\mathrm{up}}$ = 0.7 $y^{-1}$, $L_{\mathrm{low}}^{0}$ = 0.01 cm, $L_{\mathrm{up}}^{0}$ = 3 cm, $\sigma_{\mathrm{low}}$ = 0.05, and $\sigma_{\mathrm{up}}$ = 0.3).


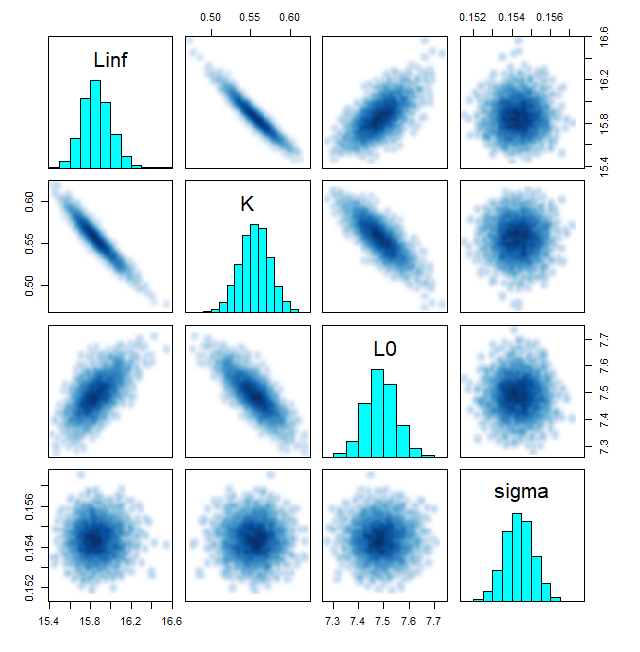


**Figure S4.** Bayesian model results for *Coregonus albula* showing the histograms for each parameter, ($L^{\infty}$ = length at infinity, $k$ = Brody´s growth coefficient, $L^{0}$ = length at birth, sigma = variance) and their correlations with each other (with prior parameters: $L_{\mathrm{low}}^{\infty}$ = 10 cm, $L_{\mathrm{up}}^{\infty}$ = 30 cm, $k_{\mathrm{low}}$ = 0.05 $y^{-1}$, $k_{\mathrm{up}}$ = 0.9 $y^{-1}$, $L_{\mathrm{low}}^{0}$ = 0.3 cm, $L_{\mathrm{up}}^{0}$ = 15 cm, $\sigma_{\mathrm{low}}$ = 0.05, and $\sigma_{\mathrm{up}}$ = 0.8).


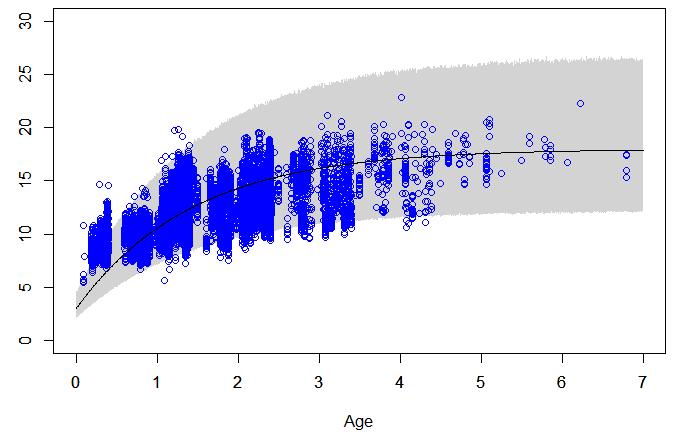


**Figure S5**. Length of *Coregonus albula* in y-axis at different ages in x-axis, data points in blue, length-at-age predicted from the Bayesian von Bertalanffy prediction in black line, and credible intervals 0.025–0.975 in grey. Prior parameters were $L_{\mathrm{low}}^{\infty}$ = 10 cm, $L_{\mathrm{up}}^{\infty}$ = 30 cm, $k_{\mathrm{low}}$ = 0.05 $y^{-1}$, $k_{\mathrm{up}}$ = 0.7 $y^{-1}$, $L_{\mathrm{low}}^{0}$ = 0.01 cm, $L_{\mathrm{up}}^{0}$ = 3 cm, $\sigma_{\mathrm{low}}$ = 0.05, and $\sigma_{\mathrm{up}}$ = 0.3.


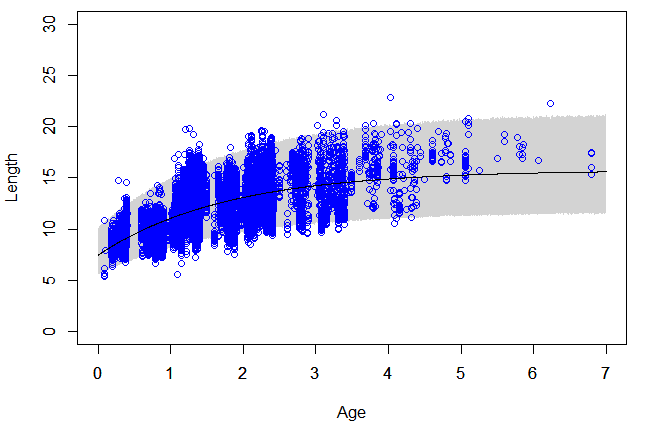


**Figure S6**. Length of *Coregonus albula* in y-axis at different ages in x-axis, data points in blue, length-at-age predicted from the Bayesian von Bertalanffy prediction in black line, and credible intervals 0.025–0.975 in grey. Prior parameters were $L_{\mathrm{low}}^{\infty}$ = 10 cm, $L_{\mathrm{up}}^{\infty}$ = 30 cm, $k_{\mathrm{low}}$ = 0.05 $y^{-1}$, $k_{\mathrm{up}}$ = 0.9 $y^{-1}$, $L_{\mathrm{low}}^{0}$ = 0.3 cm, $L_{\mathrm{up}}^{0}$= 15 cm, $\sigma_{\mathrm{low}}$ = 0.05, and $\sigma_{\mathrm{up}}$ = 0.8.

***Coregonus lavaretus***

There were 49683 whitefish *Coregonus* *lavaretus* Valenciennes 1848, individuals caught, whose length and age were measured, from Lake Oulujärvi during years 1972–2017. One larvae fish that had been caught already in March was removed, leaving 49682 individuals. *C. lavaretus* has three ecotypes in Lake Oulujärvi. In this study, all *C. lavaretus* ecotypes are still combined.

As prior parameters for Bayesian von Bertalanffy model we set a) $L_{\mathrm{low}}^{\infty}$ = 20 cm, $L_{\mathrm{up}}^{\infty}$ = 70 cm, $k_{\mathrm{low}}$ = 0.05 $y^{-1}$, $k_{\mathrm{up}}$ = 0.7 $y^{-1}$, $L_{\mathrm{low}}^{0}$ = 0.3 cm, $L_{\mathrm{up}}^{0}$= 3 cm, $\sigma_{\mathrm{low}}$ = 0.05, $\sigma_{\mathrm{up}}$ = 0.3 and as less restricted version b) we had prior parameters chosen as $L_{\mathrm{low}}^{\infty}$= 20 cm, $L_{\mathrm{up}}^{\infty}$ = 70 cm, $k_{\mathrm{low}}$ = 0.05 $y^{-1}$, $k_{\mathrm{up}}$ = 0.9 $y^{-1}$, $L_{\mathrm{low}}^{0}$ = 0.3 cm, $L_{\mathrm{up}}^{0}$ = 15 cm, $\sigma_{\mathrm{low}}$ = 0.05 and $\sigma_{\mathrm{up}}$ = 0.8. The prior option a) (**Figure S7**) restricted the histogram distribution of $L^{0}$, while option b) did not (**Figure S8**). The predictions from the Bayesian models are shown in **figures S9** (with a) prior parameters) and **S10** (with b) prior parameters).

We used rounded mean $L^{\infty}$ (33 cm) and $k$ (0.277 $y^{-1}$) values from the less strict Bayesian model in the ATNE model. In the literature *C. lavaretus* larvae length after first weeks of hatching was on average $E\left( L_{\mathrm{whi}}^{0+} \right)=$ 1.3 cm with standard deviation $\mathrm{SD}\left( L_{\mathrm{whi}}^{0+} \right)=$ 0.22 cm (Sutela & Huusko, 2000). We set $L_{\mathrm{whi}}^{0}=E\left( L_{\mathrm{whi}}^{0+} \right)-\mathrm{SD}\left( L_{\mathrm{whi}}^{0+} \right)=$ 1.1 cm in the ATNE model calculated from the literature information.


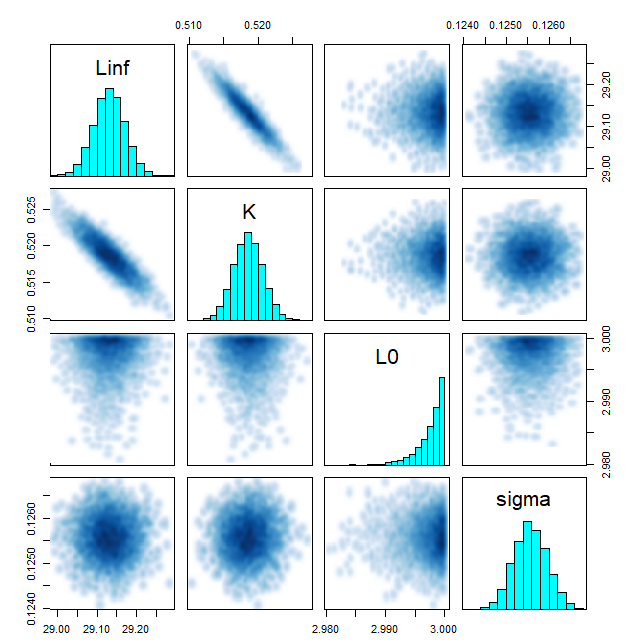


**Figure S7.** Bayesian model results for *Coregonus lavaretus* showing the histograms for each parameter, ($L^{\infty}$ = length at infinity, $k$ = Brody´s growth coefficient, $L^{0}$ = length at birth, sigma = variance) and their correlations with each other (with prior parameters: $L_{\mathrm{low}}^{\infty}$ = 20 cm, $L_{\mathrm{up}}^{\infty}$ = 70 cm, $k_{\mathrm{low}}$ = 0.05 $y^{-1}$, $k_{\mathrm{up}}$ = 0.7 $y^{-1}$, $L_{\mathrm{low}}^{0}$ = 0.3 cm, $L_{\mathrm{up}}^{0}$ = 3 cm, $\sigma_{\mathrm{low}}$ = 0.05, and $\sigma_{\mathrm{up}}$ = 0.3).


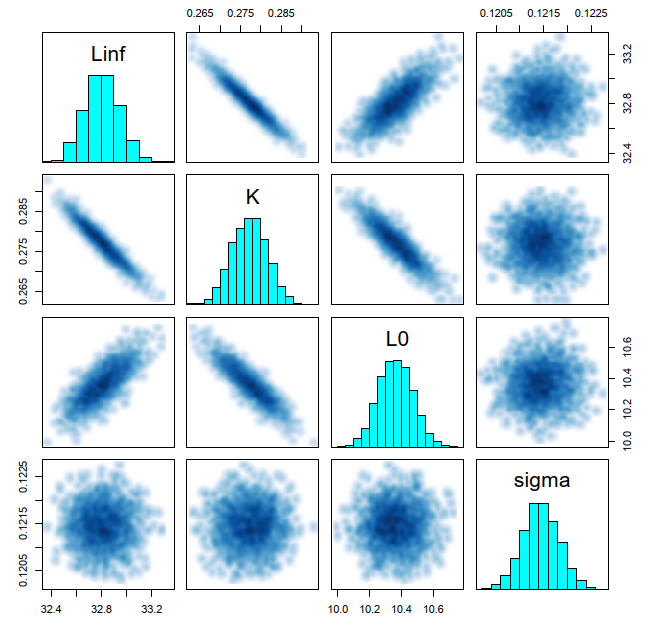


**Figure S8.** Bayesian model results for *Coregonus lavaretus* showing the histograms for each parameter, ($L^{\infty}$ = length at infinity, $k$ = Brody´s growth coefficient, $L^{0}$ = length at birth, sigma = variance) and their correlations with each other (with prior parameters: $L_{\mathrm{low}}^{\infty}$ = 20 cm, $L_{\mathrm{up}}^{\infty}$ = 70 cm, $k_{\mathrm{low}}$ = 0.05 $y^{-1}$, $k_{\mathrm{up}}$ = 0.9 $y^{-1}$, $L_{\mathrm{low}}^{0}$ = 0.3 cm, $L_{\mathrm{up}}^{0}$ = 15 cm, $\sigma_{\mathrm{low}}$ = 0.05, and $\sigma_{\mathrm{up}}$ = 0.8).


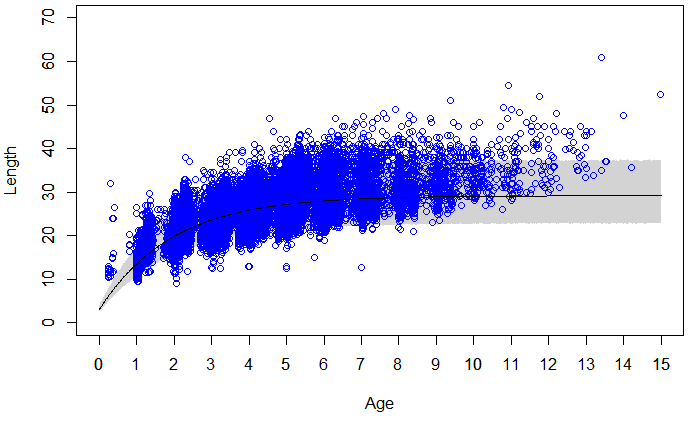


**Figure S9.** Length of *Coregonus lavaretus* in y-axis at different ages in x-axis, data points in blue, length-at-age predicted from the Bayesian von Bertalanffy prediction in black line, and credible intervals 0.025–0.975 in grey. Prior parameters were $L_{\mathrm{low}}^{\infty}$ = 20 cm, $L_{\mathrm{up}}^{\infty}$ = 70 cm, $k_{\mathrm{low}}$ = 0.05 $y^{-1}$, $k_{\mathrm{up}}$ = 0.7 $y^{-1}$, $L_{\mathrm{low}}^{0}$ = 0.3 cm, $L_{\mathrm{up}}^{0}$ = 3 cm, $\sigma_{\mathrm{low}}$ = 0.05, and $\sigma_{\mathrm{up}}$ = 0.3.


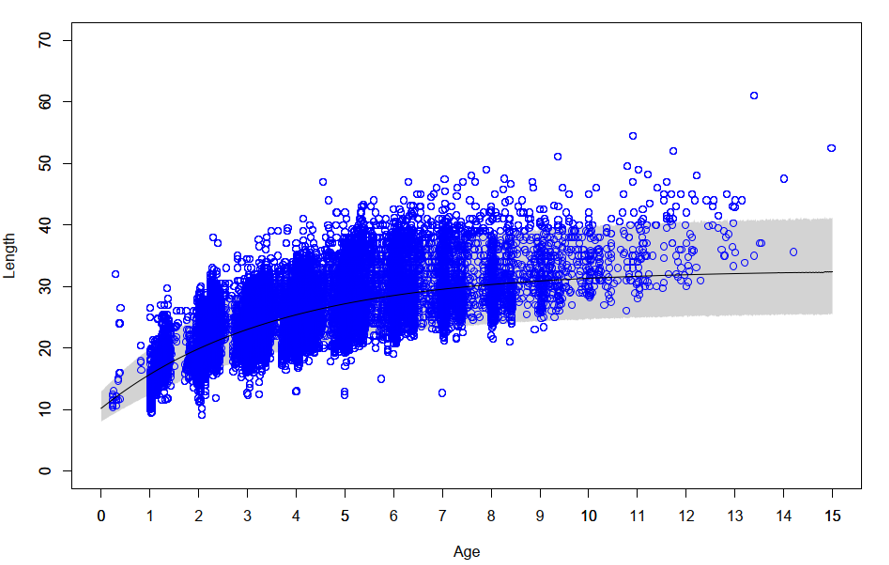


**Figure S10.** Length of *Coregonus lavaretus* in y-axis at different ages in x-axis, data points in blue, length-at-age predicted from the Bayesian von Bertalanffy prediction in black line, and credible intervals 0.025–0.975 in grey. Prior parameters were $L_{\mathrm{low}}^{\infty}$ = 20 cm, $L_{\mathrm{up}}^{\infty}$ = 70 cm, $k_{\mathrm{low}}$ = 0.05 $y^{-1}$, $k_{\mathrm{up}}$ = 0.9 $y^{-1}$, $L_{\mathrm{low}}^{0}$ = 0.3 cm, $L_{\mathrm{up}}^{0}$ = 15 cm, $\sigma_{\mathrm{low}}$ = 0.05, and $\sigma_{\mathrm{up}}$ = 0.8.

***Osmerus eperlanus***

There were 9240 smelt *Osmerus eperlanus* L. 1758, individuals caught, whose length and age were measured, from Lake Oulujärvi during years 1989 and 1994–2017.

As prior parameters for Bayesian von Bertalanffy model we set a) $L_{\mathrm{low}}^{\infty}$ = 10 cm, $L_{\mathrm{up}}^{\infty}$ = 30 cm, $k_{\mathrm{low}}$ = 0.05 $y^{-1}$, $k_{\mathrm{up}}$ = 0.7 $y^{-1}$, $L_{\mathrm{low}}^{0}$ = 0.3 cm, $L_{\mathrm{up}}^{0}$ = 3 cm, $\sigma_{\mathrm{low}}$ = 0.05, $\sigma_{\mathrm{up}}$ = 0.3, and as less restricted version b) we had prior parameters chosen as $L_{\mathrm{low}}^{\infty}$ = 10 cm, $L_{\mathrm{up}}^{\infty}$ = 30 cm, $k_{\mathrm{low}}$ = 0.05 $y^{-1}$, $k_{\mathrm{up}}$ = 0.9 $y^{-1}$, $L_{\mathrm{low}}^{0}$ = 0.3 cm, $L_{\mathrm{up}}^{0}$ = 15 cm, $\sigma_{\mathrm{low}}$ = 0.05 and $\sigma_{\mathrm{up}}$ = 0.8. The prior option a) (**Figure S11**) restricted the histogram distributions of $L^{0}$, while option b did not (**Figure S12**). The predictions from the Bayesian models are shown in **figures** **S13** (with a) prior parameters) and **S14** (with b) prior parameters).

$L^{\infty}$ (17 cm) and $k$ (0.171 $y^{-1}$) parameter mean values got with the less strict prior parameters were used in ATNE model. The $L^{0}$ parameter was again quite large for just hatched larvae. According to Sutela and Hyvärinen (2002) the mean length of 0+ *O. eperlanus* on tenth of August was $E\left( L_{\mathrm{sme}}^{0+} \right)=2.9 \mathrm{cm}$ with standard deviation, $\mathrm{SD}\left( L_{\mathrm{sme}}^{0+} \right)=1.5 \mathrm{cm}$. We set $L_{\mathrm{sme}}^{0}=E\left( L_{\mathrm{sme}}^{0+} \right)-\mathrm{SD}\left( L_{\mathrm{sme}}^{0+} \right)=1.4 \mathrm{cm}$ in the ATNE model.


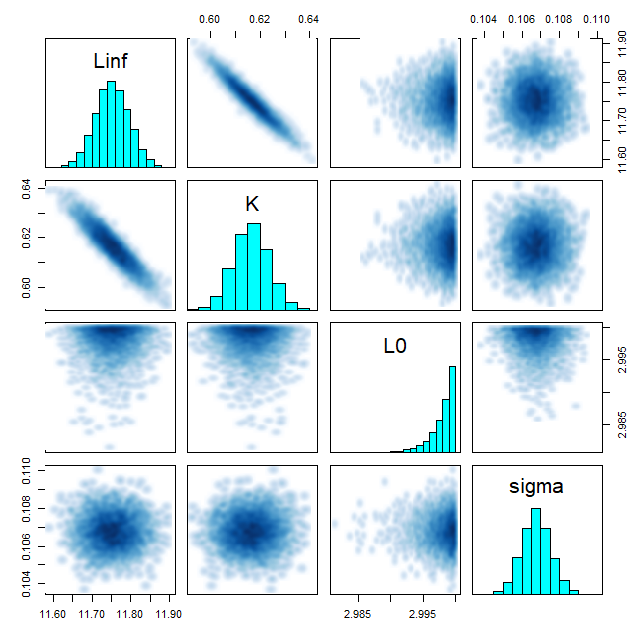


**Figure S11.** Bayesian model results for *Osmerus eperlanus* showing the histograms for each parameter, ($L^{\infty}$ = length at infinity, $k$ = Brody´s growth coefficient, $L^{0}$ = length at birth, sigma = variance) and their correlations with each other (with prior parameters: $L_{\mathrm{low}}^{\infty}$ = 10 cm, $L_{\mathrm{up}}^{\infty}$ = 30 cm, $k_{\mathrm{low}}$ = 0.05 $y^{-1}$, $k_{\mathrm{up}}$ = 0.7 $y^{-1}$, $L_{\mathrm{low}}^{0}$ = 0.3 cm, $L_{\mathrm{up}}^{0}$ = 3 cm, $\sigma_{\mathrm{low}}$ = 0.05, and $\sigma_{\mathrm{up}}$ = 0.3).


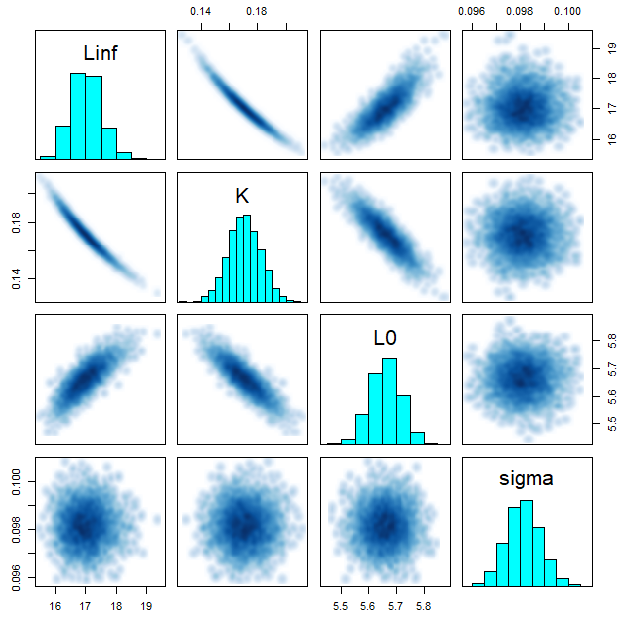


**Figure S12.** Bayesian model results for *Osmerus eperlanus* showing the histograms for each parameter, ($L^{\infty}$ = length at infinity, $k$ = Brody´s growth coefficient, $L^{0}$ = length at birth, sigma = variance) and their correlations with each other (with prior parameters: $L_{\mathrm{low}}^{\infty}$ = 10 cm, $L_{\mathrm{up}}^{\infty}$ = 30 cm, $k_{\mathrm{low}}$ = 0.05 $y^{-1}$, $k_{\mathrm{up}}$ = 0.9 $y^{-1}$, $L_{\mathrm{low}}^{0}$ = 0.3 cm, $L_{\mathrm{up}}^{0}$ = 15 cm, $\sigma_{\mathrm{low}}$ = 0.05 and $\sigma_{\mathrm{up}}$ = 0.8).


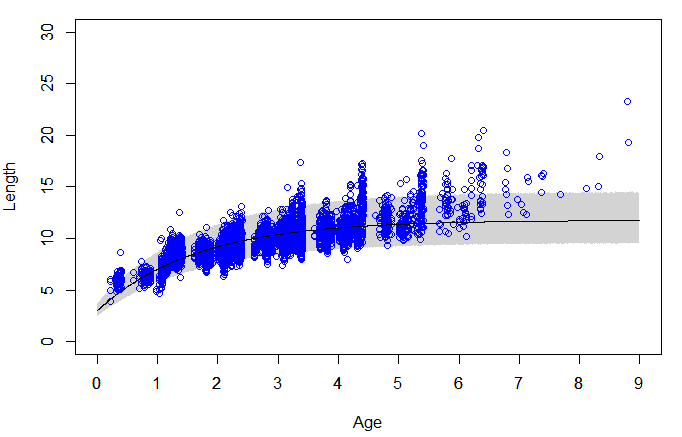


**Figure S13.** Length of *Osmerus eperlanus* in y-axis at different ages in x-axis, data points in blue, length-at-age predicted from the Bayesian von Bertalanffy prediction in black line, and credible intervals 0.025–0.975 in grey. Prior parameters were $L_{\mathrm{low}}^{\infty}$= 10 cm, $L_{\mathrm{up}}^{\infty}$ = 30 cm, $k_{\mathrm{low}}$ = 0.05 $y^{-1}$, $k_{\mathrm{up}}$ = 0.7 $y^{-1}$, $L_{\mathrm{low}}^{0}$= 0.3 cm, $L_{\mathrm{up}}^{0}$ = 3 cm, $\sigma_{\mathrm{low}}$ = 0.05, $\sigma_{\mathrm{up}}$ = 0.3.


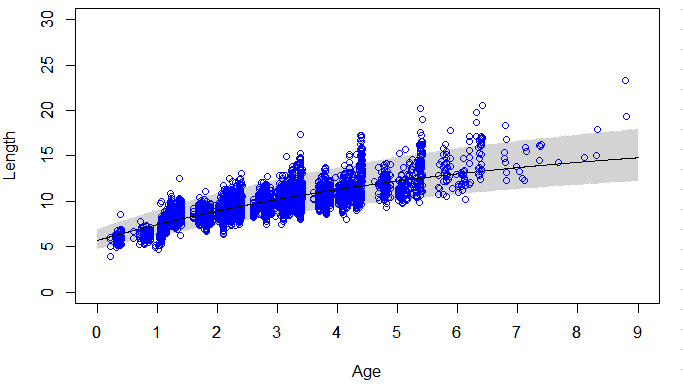


**Figure S14.** Length of *Osmerus eperlanus* in y-axis at different ages in x-axis, data points in blue, length-at-age predicted from the Bayesian von Bertalanffy prediction in black line, and credible intervals 0.025–0.975 in grey. Prior parameters were $L_{\mathrm{low}}^{\infty}$ = 10 cm, $L_{\mathrm{up}}^{\infty}$ = 30 cm, $k_{\mathrm{low}}$ = 0.05 $y^{-1}$, $k_{\mathrm{up}}$ = 0.9 $y^{-1}$, $L_{\mathrm{low}}^{0}$ = 0.3 cm, $L_{\mathrm{up}}^{0}$ = 15 cm, $\sigma_{\mathrm{low}}$ = 0.05 and $\sigma_{\mathrm{up}}$ = 0.8.

***Perca fluviatilis***

For perch *Perca fluviatilis* L. 1758, we had the data from 209 individuals caught, whose length and age were measured, from Lake Oulujärvi during years 2007 and 2012. Additionally, we had data from 141 *P. fluviatilis* larvae pond-reared in the Paltamo Research Station whose length had been measured from photographs. They were given estimated birth date of June 18^th^ 2015 as the exact birth date was not known.

Prior parameters for Bayesian von Bertalanffy model (**Figure S15**) were set as $L_{\mathrm{low}}^{\infty}$ = 20 cm, $L_{\mathrm{up}}^{\infty}$= 120 cm, $k_{\mathrm{low}}$ = 0.03 $y^{-1}$, $k_{\mathrm{up}}$ = 0.9 $y^{-1}$, $L_{\mathrm{low}}^{0}$ = 0.01 cm, $L_{\mathrm{up}}^{0}$ = 3 cm, $\sigma_{\mathrm{low}}$ = 0.05 and $\sigma_{\mathrm{up}}$ = 0.8. The model predicted growth reasonably well (**Figure S16**). $L^{0}$ was perhaps too high, maybe because the first measurements of larvae *P. fluviatilis* were not done before August.

We set $L^{0}$ to 1.7 cm, so it was similar to the $L^{0}$ of *S. lucioperca*. We used $L^{\infty}$ (71 cm) and $k$ (0.057 $y^{-1}$) values for the ATNE model.


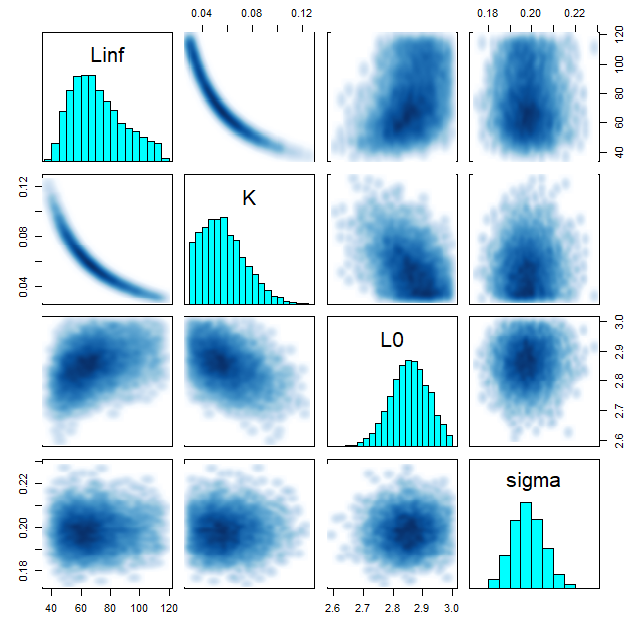


**Figure S15.** Bayesian model results for *Perca fluviatilis* showing the histograms for each parameter, ($L^{\infty}$ = length at infinity, $k$ = Brody´s growth coefficient, $L^{0}$ = length at birth, sigma = variance) and their correlations with each other (with prior parameters: $L_{\mathrm{low}}^{\infty}$ = 20 cm, $L_{\mathrm{up}}^{\infty}$ = 120 cm, $k_{\mathrm{low}}$ = 0.03 $y^{-1}$, $k_{\mathrm{up}}$ = 0.9 $y^{-1}$, $L_{\mathrm{low}}^{0}$ = 0.01 cm, $L_{\mathrm{up}}^{0}$ = 3 cm, $\sigma_{\mathrm{low}}$ = 0.05 and $\sigma_{\mathrm{up}}$ = 0.8).


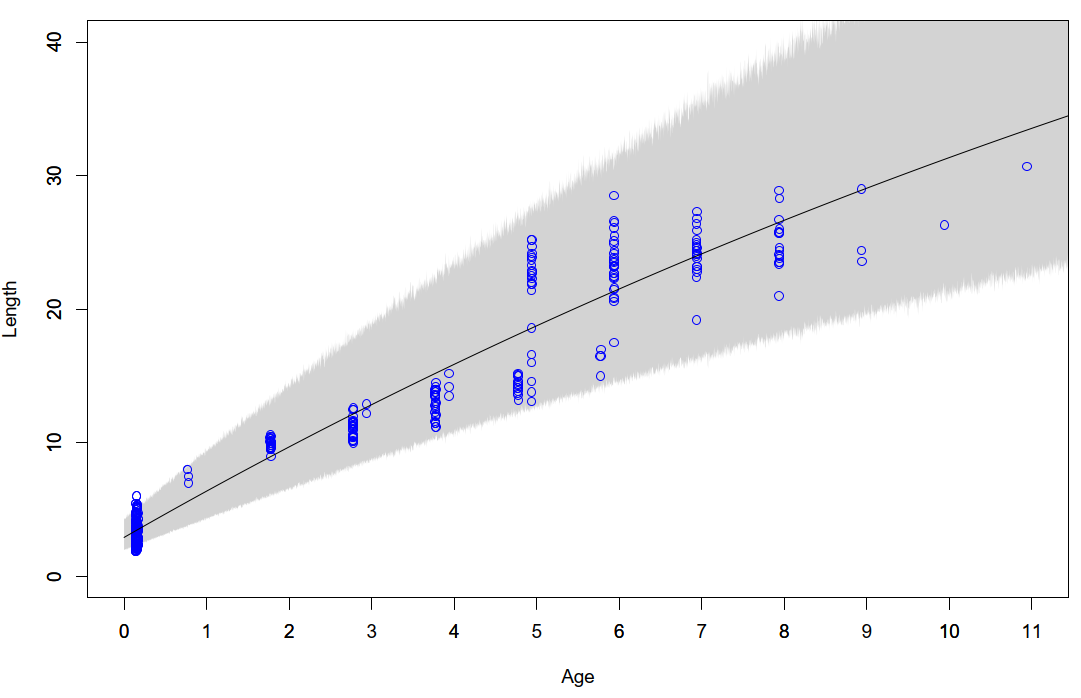


**Figure S16.** Length of *Perca fluviatilis* in y-axis at different ages in x-axis, data points in blue, length-at-age predicted from the Bayesian von Bertalanffy prediction in black line, and credible intervals 0.025–0.975 in grey. Prior parameters were $L_{\mathrm{low}}^{\infty}$= 20 cm, $L_{\mathrm{up}}^{\infty}$= 120 cm, $k_{\mathrm{low}}$ = 0.03 $y^{-1}$, $k_{\mathrm{up}}$ = 0.9 $y^{-1}$, $L_{\mathrm{low}}^{0}$ = 0.01 cm, $L_{\mathrm{up}}^{0}$ = 3 cm, $\sigma_{\mathrm{low}}$ = 0.05 and $\sigma_{\mathrm{up}}$ = 0.8.

***Salmo trutta***

There were 608 brown trout *Salmo trutta* L. 1758, individuals caught, whose length and age were measured, from Lake Oulujärvi during years 1995–1997 and 2001–2002.

As prior parameters we set $L_{\mathrm{low}}^{\infty}$= 40 cm, $L_{\mathrm{up}}^{\infty}$= 140 cm, $k_{\mathrm{low}}$ = 0.02 $y^{-1}$, $k_{\mathrm{up}}$ = 0.9 $y^{-1}$, $L_{\mathrm{low}}^{0}$ = 0.03 cm, $L_{\mathrm{up}}^{0}$ = 3 cm, $\sigma_{\mathrm{low}}$ = 0.05, $\sigma_{\mathrm{up}}$ = 0.8. $L^{0}$ histogram did not have clear peak (**Figure S17**). The model seemed to predict the growth of *S. trutta* quite well (**Figure S18**).

We used the mean values of $L^{\infty}$ (83 cm), $k$ (0.198 $y^{-1}$) and $L^{0}$ (1.7 cm) for the ATNE model.


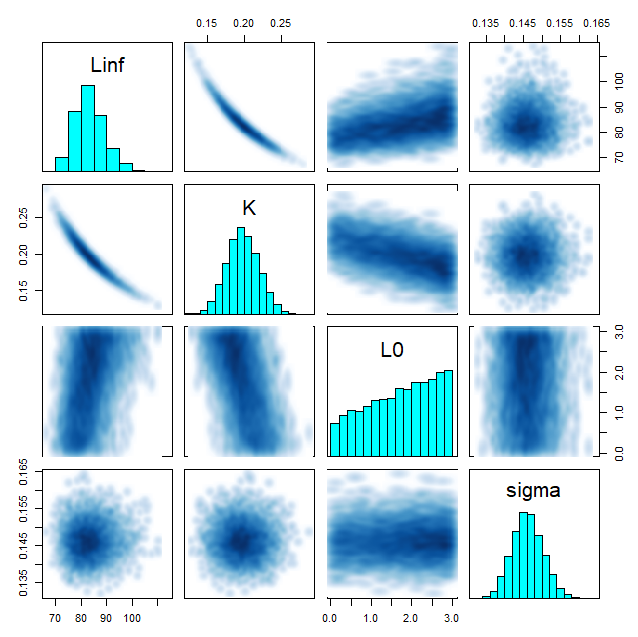


**Figure S17.** Bayesian model results for *Salmo trutta* showing the histograms for each parameter, ($L^{\infty}$ = length at infinity, $k$ = Brody´s growth coefficient, $L^{0}$ = length at birth, sigma = variance) and their correlations with each other (with prior parameters: $L_{\mathrm{low}}^{\infty}$ = 40 cm, $L_{\mathrm{up}}^{\infty}$ = 140 cm, $k_{\mathrm{low}}$ = 0.02 $y^{-1}$, $k_{\mathrm{up}}$ = 0.9 $y^{-1}$, $L_{\mathrm{low}}^{0}$ = 0.03 cm, $L_{\mathrm{up}}^{0}$ = 3 cm, $\sigma_{\mathrm{low}}$ = 0.05, $\sigma_{\mathrm{up}}$ = 0.8).


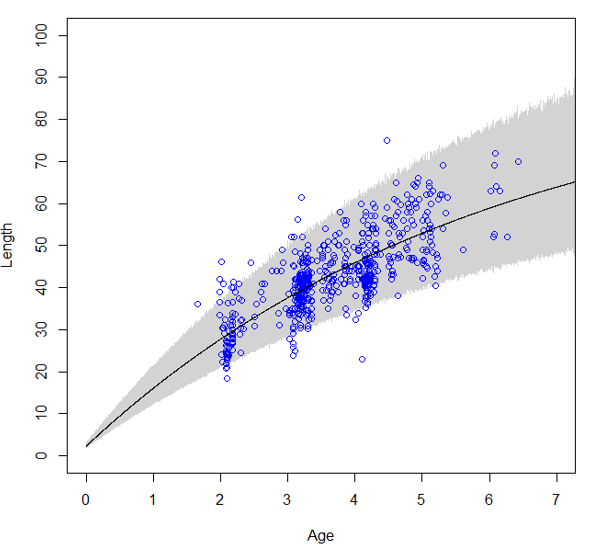


**Figure S18.** Length of *Salmo trutta* in y-axis at different ages in x-axis, data points in blue, length-at-age predicted from the Bayesian von Bertalanffy prediction in black line, and credible intervals 0.025–0.975 in grey. Prior parameters were $L_{\mathrm{low}}^{\infty}$ = 40 cm, $L_{\mathrm{up}}^{\infty}$ = 140 cm, $k_{\mathrm{low}}$ = 0.02 $y^{-1}$, $k_{\mathrm{up}}$ = 0.9 $y^{-1}$, $L_{\mathrm{low}}^{0}$ = 0.03 cm, $L_{\mathrm{up}}^{0}$ = 3 cm, $\sigma_{\mathrm{low}}$ = 0.05, $\sigma_{\mathrm{up}}$ = 0.8.

**SUPPLEMENTARY RESULTS – EVOLUTION OF GROWTH PARAMETERS**

**
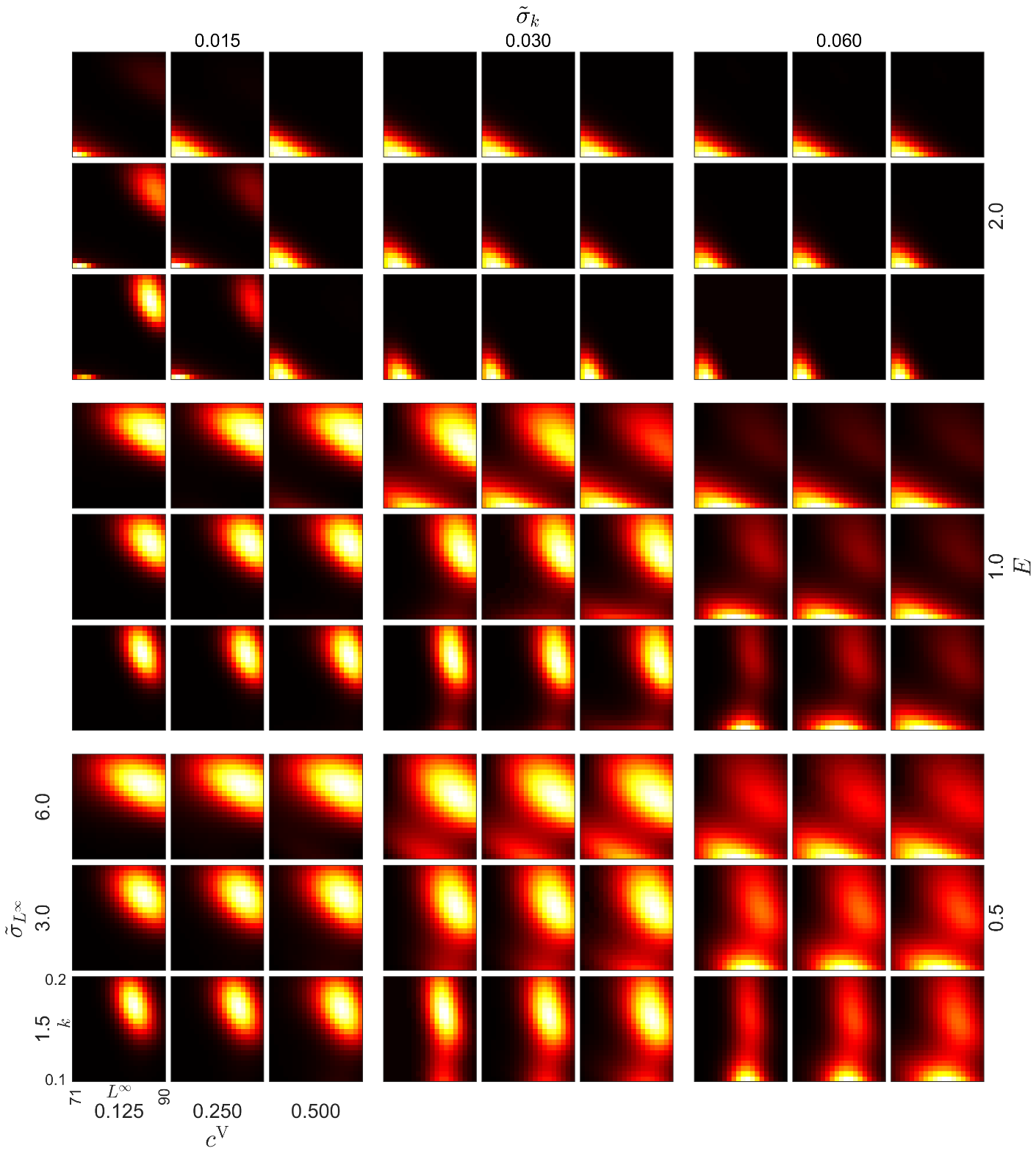
**

**Figure S19.** *Sander lucioperca* age 6+ trait distribution after 100 years of size-selective fishing in the simulation, with a low ($\rho$ = -0.35) level of correlation between $k$ (Brody’s growth coefficient) and $L^{\infty}$ (asymptotic length). The figure illustrates the influence of varying instantaneous fishing mortalities $(E)$, and genotypic $(c^{V})$ and phenotypic $(\tilde{\sigma}_{L^{\infty}}, \tilde{\sigma}_{k})$ variance parameters on the eco-evolutionary impacts of size-selective fishing. The horizontal axis ($L^{\infty}$) represents the asymptotic length (cm), and the vertical axis ($k$) represents Brody’s growth coefficient ($y^{-1}$). Brighter colours indicate higher trait density. Mean of the starting distribution is in the middle of the grid.

**
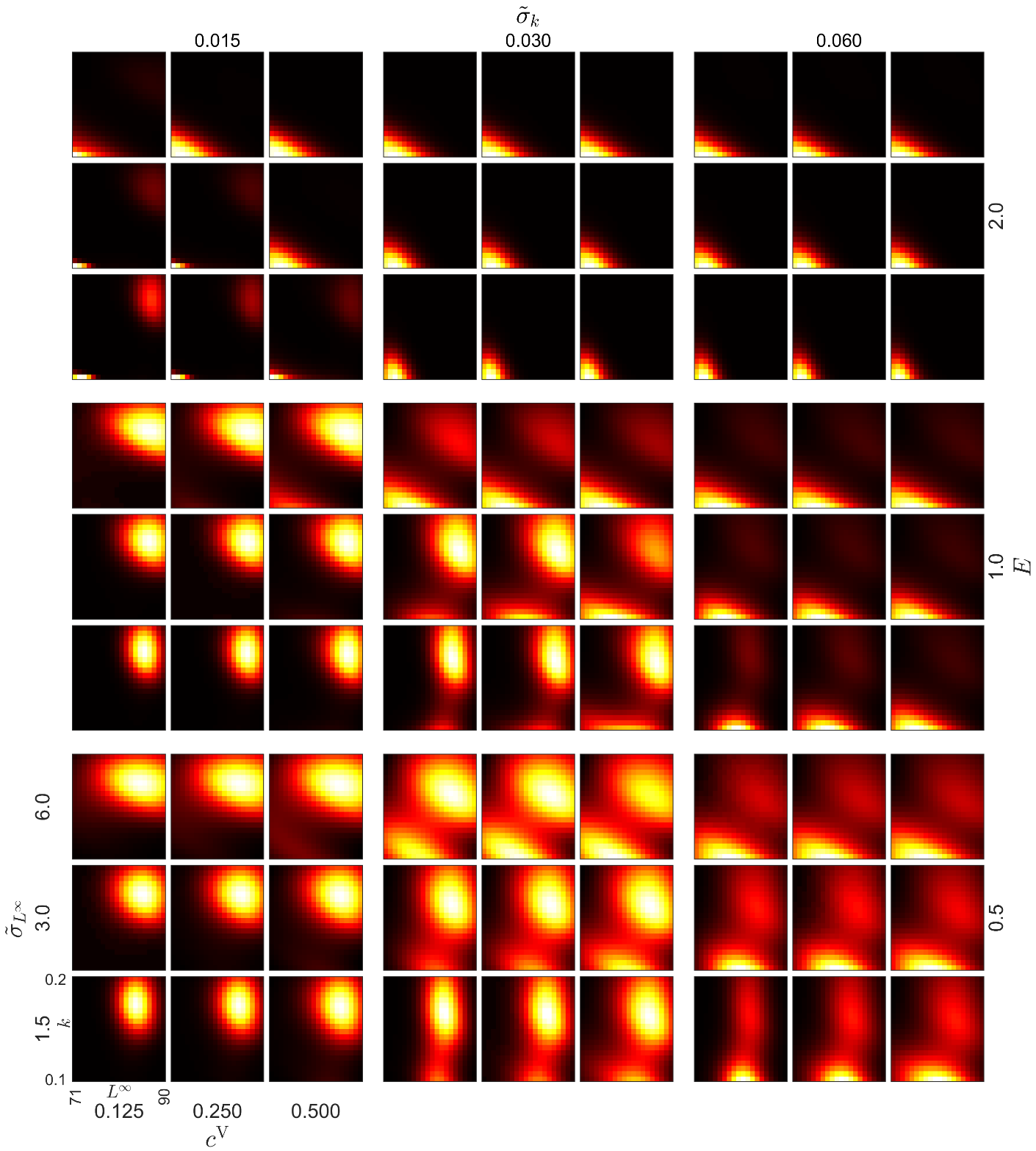
**

**Figure S20.** *Sander lucioperca* age 6+ trait distribution after 100 years of size-selective fishing in the simulation, with no correlation ($\rho$ = 0) between $k$ (Brody’s growth coefficient) and $L^{\infty}$ (asymptotic length). The figure illustrates the influence of varying instantaneous fishing mortalities $(E)$, and genotypic $(c^{V})$ and phenotypic $(\tilde{\sigma}_{L^{\infty}}, \tilde{\sigma}_{k})$ variance parameters on the eco-evolutionary impacts of size-selective fishing. The horizontal axis ($L^{\infty}$) represents the asymptotic length (cm), and the vertical axis ($k$) represents Brody’s growth coefficient ($y^{-1}$). Brighter colours indicate higher trait density. Mean of the starting distribution is in the middle of the grid.

**
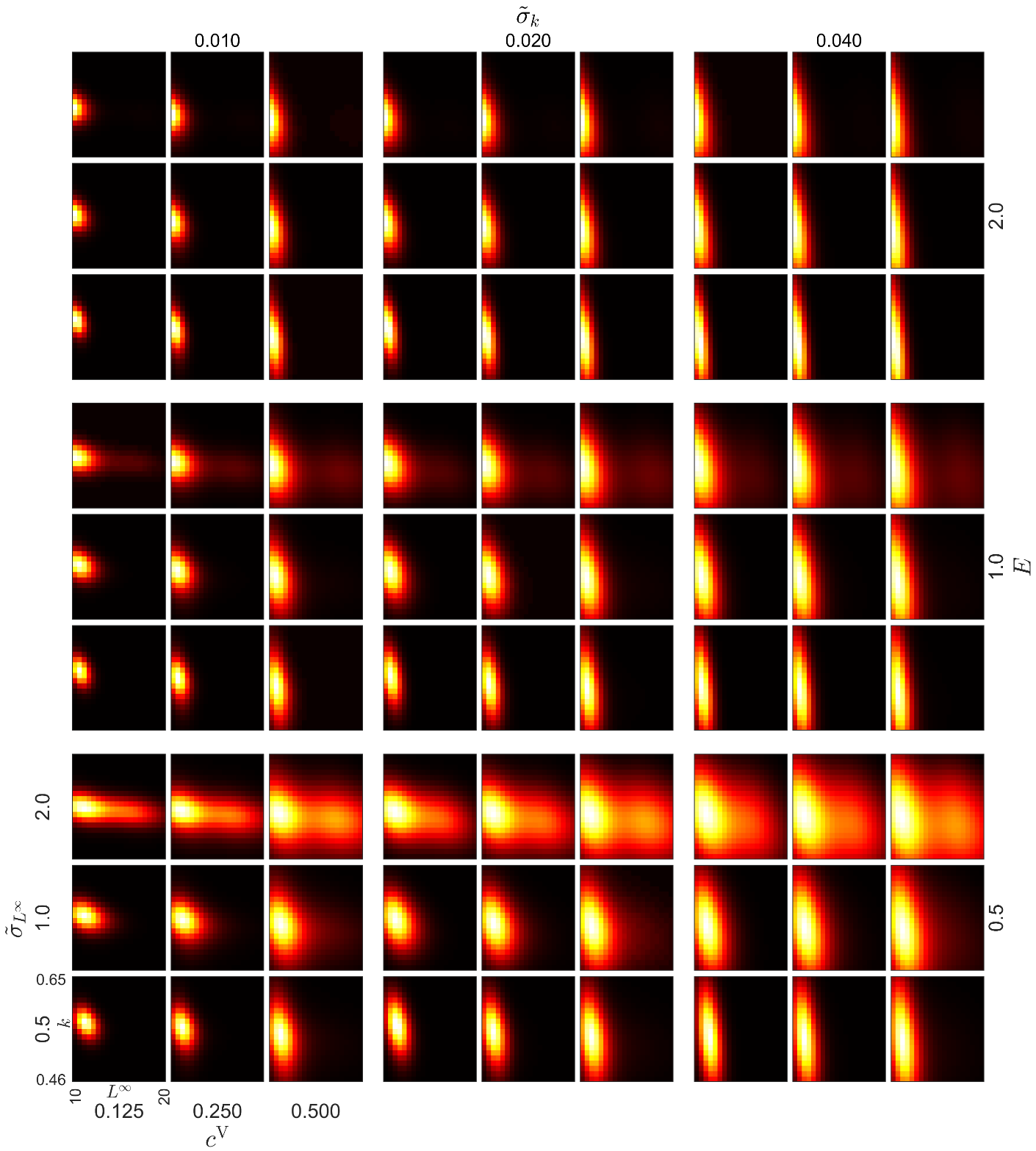
**

**Figure S21.** *Coregonus albula* age 4+ trait distribution after 100 years of size-selective fishing in the simulation, with a low ($\rho$ = -0.35) level of correlation between $k$ (Brody’s growth coefficient) and $L^{\infty}$ (asymptotic length). The figure illustrates the influence of varying instantaneous fishing mortalities $(E)$, and genotypic $(c^{V})$ and phenotypic $(\tilde{\sigma}_{L^{\infty}}, \tilde{\sigma}_{k})$ variance parameters on the eco-evolutionary impacts of size-selective fishing. The horizontal axis ($L^{\infty}$) represents the asymptotic length (cm), and the vertical axis ($k$) represents Brody’s growth coefficient ($y^{-1}$). Brighter colours indicate higher trait density. Mean of the starting distribution is in the middle of the grid.

**
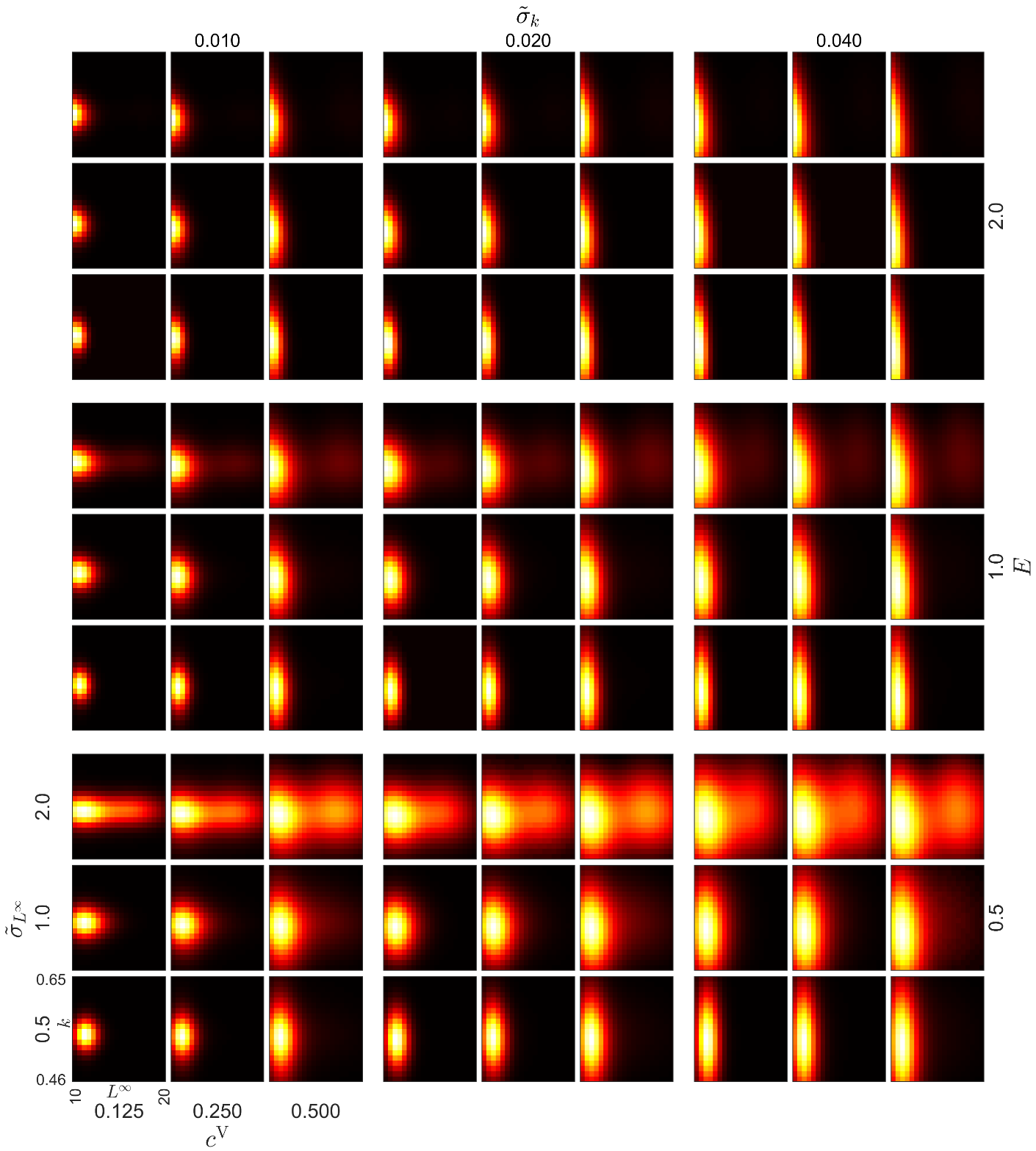
**

**Figure S22.** *Coregonus albula* age 4+ trait distribution after 100 years of size-selective fishing in the simulation, with no correlation ($\rho$ = 0) between $k$ (Brody’s growth coefficient) and $L^{\infty}$ (asymptotic length). The figure illustrates the influence of varying instantaneous fishing mortalities $(E)$, and genotypic $(c^{V})$ and phenotypic $(\tilde{\sigma}_{L^{\infty}}, \tilde{\sigma}_{k})$ variance parameters on the eco-evolutionary impacts of size-selective fishing. The horizontal axis ($L^{\infty}$) represents the asymptotic length (cm), and the vertical axis ($k$) represents Brody’s growth coefficient ($y^{-1}$). Brighter colours indicate higher trait density. Mean of the starting distribution is in the middle of the grid.

**SUPPLEMENTARY RESULTS – HERITABILITIES**

The heritability of $L^{\infty}$ in *S. lucioperca* ranged from 0.3125 to 0.7225 with a low level of correlation between $k$ and $L^{\infty}$($\rho$ = -0.35) and from 0.5112 to 1.0241 with no correlation between $k$ and $L^{\infty}$ ($\rho$ = 0). The heritability of $k$ ranged from 0.3094 to 0.6915 with a low level of correlation between $k$ and $L^{\infty}$ ($\rho$ = -0.35) and from 0.4593 to 1.0252 with no correlation between $k$ and $L^{\infty}$($\rho$ = 0).

The heritability of $L^{\infty}$ in *C. albula* ranged from 0.2982 to 0.7281 with a low level of correlation between $k$ and $L^{\infty}$ ($\rho$ = -0.35) and from 0.4582 to 0.9972 with no correlation between $k$ and $L^{\infty}$ ($\rho$ = 0). The heritability of $k$ ranged from 0.5011 to 0.7263 with a low level of correlation between $k$ and $L^{\infty}$ ($\rho$ = -0.35) and from 0.6991 to 0.9964 with no correlation between $k$ and $L^{\infty}$($\rho$ = 0).

**
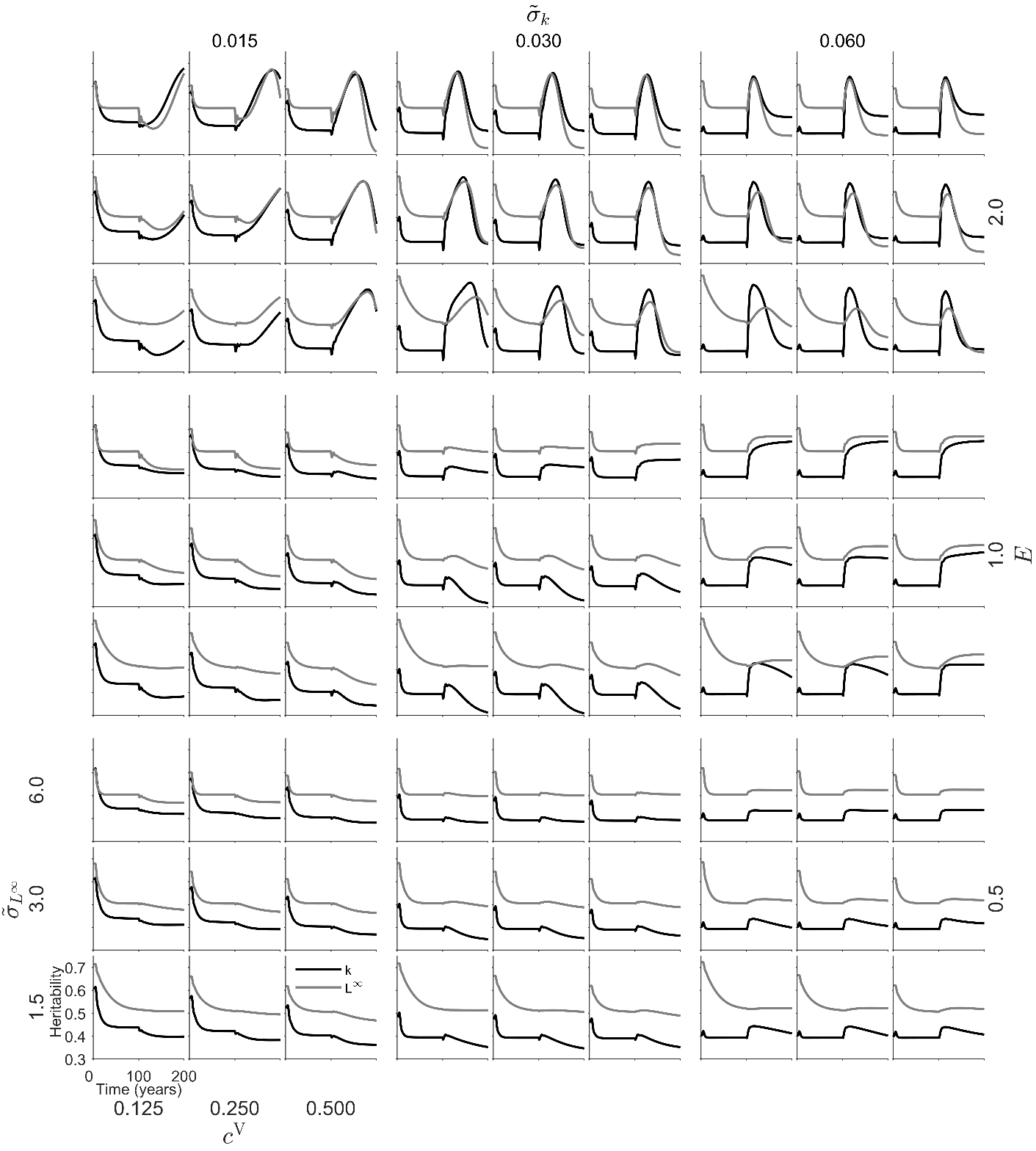
**

**Figure S23.** Heritability of *Sander lucioperca* traits; asymptotic length ($L^{\infty}$; grey) and Brody’s growth coefficient ($k$; black) over a 200-year simulation, with a low ($\rho$ = -0.35) level of correlation between $k$ and $L^{\infty}$. The figure illustrates the influence of varying instantaneous fishing mortalities $(E)$, and genotypic $(c^{V})$ and phenotypic $(\tilde{\sigma}_{L^{\infty}}, \tilde{\sigma}_{k})$ variance parameters on the heritability. Fishing activities commence at year 101. The horizontal axis represents time, while the vertical axis depicts heritability values. The trends in heritability reflect the dynamic evolution of the depicted traits during the simulation.

**
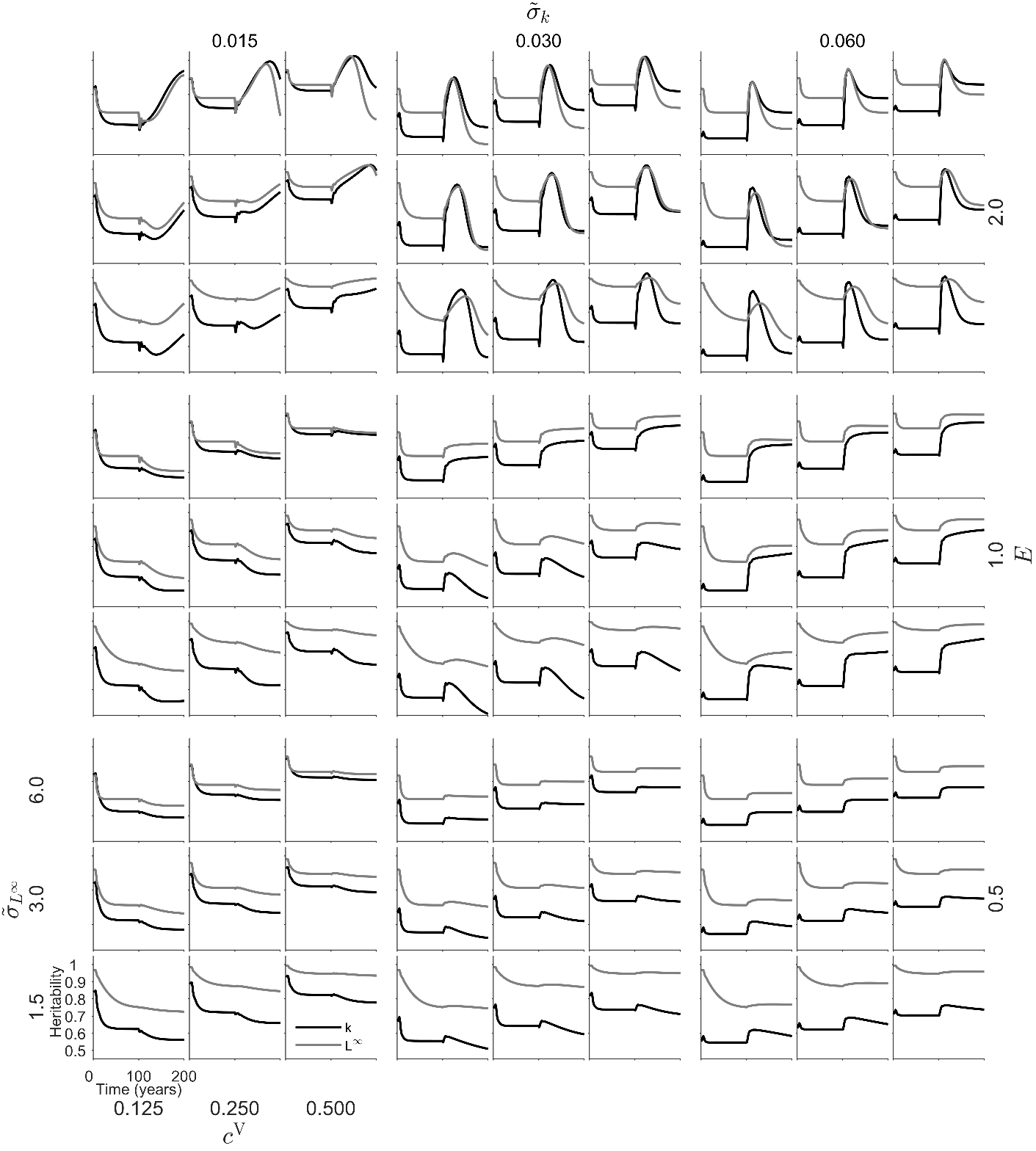
**

**Figure S24.** Heritability of *Sander lucioperca* traits; asymptotic length ($L^{\infty}$; grey) and Brody’s growth coefficient ($k$; black) over a 200-year simulation, with no correlation ($\rho$ = 0) between $k$ and $L^{\infty}$. The figure illustrates the influence of varying instantaneous fishing mortalities $(E)$, and genotypic $(c^{V})$ and phenotypic $(\tilde{\sigma}_{L^{\infty}}, \tilde{\sigma}_{k})$ variance parameters on the heritability. Fishing activities commence at year 101. The horizontal axis represents time, while the vertical axis depicts heritability values. The trends in heritability reflect the dynamic evolution of the depicted traits during the simulation.


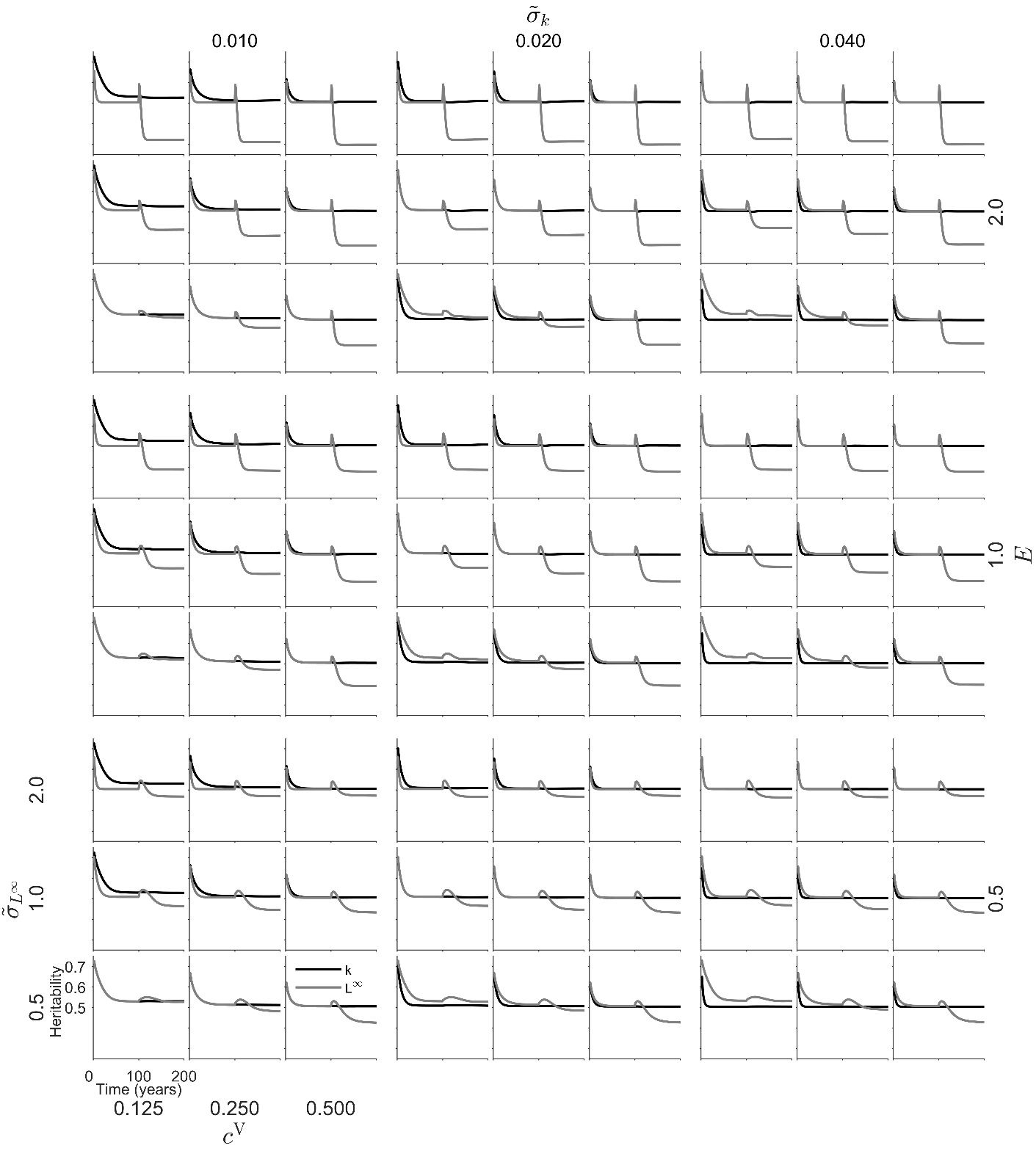


**Figure S25.** Heritability of *Coregonus albula* traits; asymptotic length ($L^{\infty}$; grey) and Brody’s growth coefficient ($k$; black) over a 200-year simulation, with a low ($\rho$ = -0.35) level of correlation between $k$ and $L^{\infty}$ . The figure illustrates the influence of varying instantaneous fishing mortalities $(E)$, and genotypic $(c^{V})$ and phenotypic $(\tilde{\sigma}_{L^{\infty}}, \tilde{\sigma}_{k})$ variance parameters on the heritability. Fishing activities commence at year 101. The horizontal axis represents time, while the vertical axis depicts heritability values. The trends in heritability reflect the dynamic evolution of the depicted traits during the simulation.


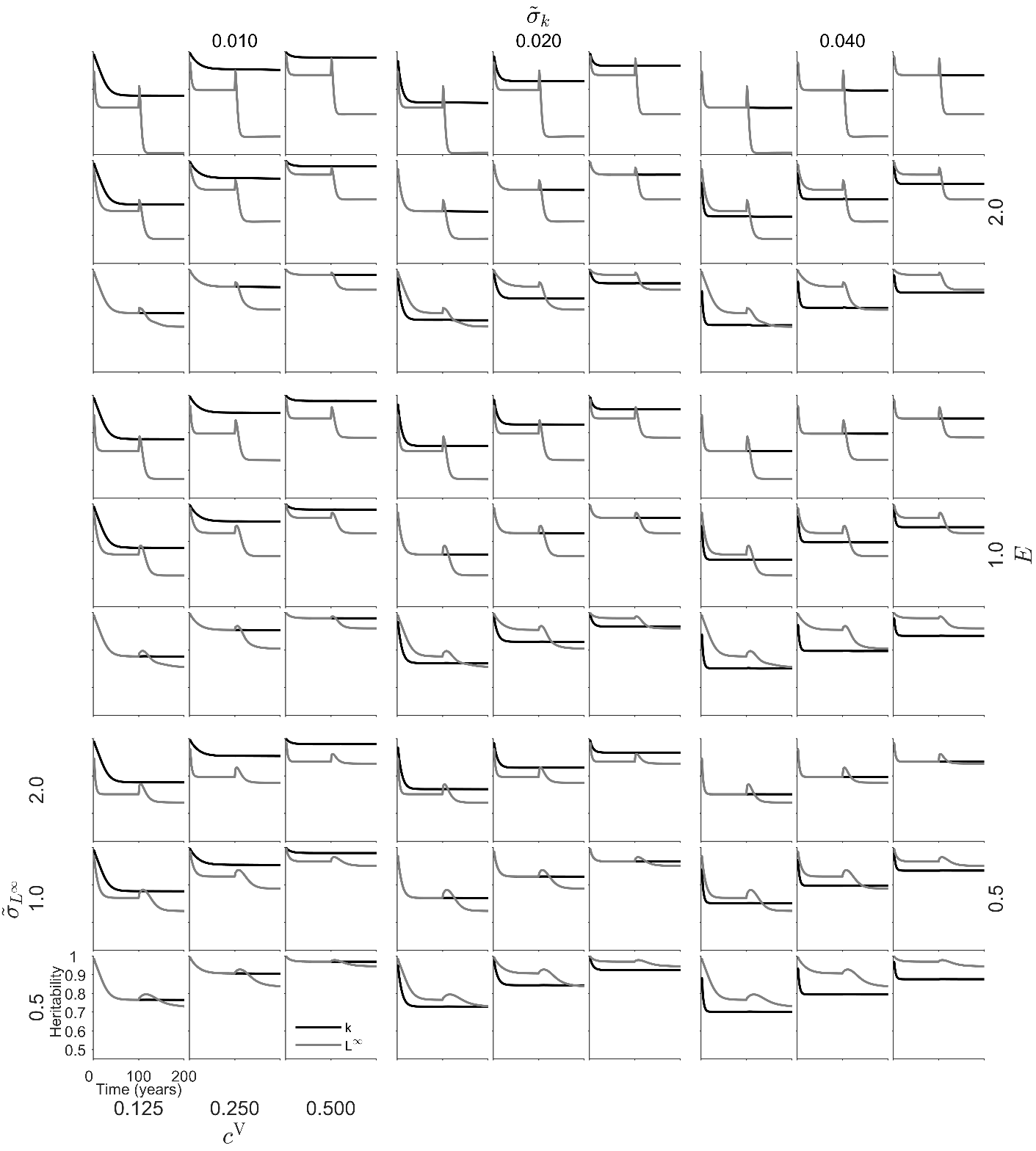


**Figure S26.** Heritability of *Coregonus albula* traits; asymptotic length ($L^{\infty}$; grey) and Brody’s growth coefficient ($k$; black) over a 200-year simulation, with no correlation ($\rho$ = 0) between $k$ and $L^{\infty}$ . The figure illustrates the influence of varying instantaneous fishing mortalities $(E)$, and genotypic $(c^{V})$ and phenotypic $(\tilde{\sigma}_{L^{\infty}}, \tilde{\sigma}_{k})$ variance parameters on the heritability. Fishing activities commence at year 101. The horizontal axis represents time, while the vertical axis depicts heritability values. The trends in heritability reflect the dynamic evolution of the depicted traits during the simulation.

**SUPPLEMENTARY RESULTS – VARIANCES**

**
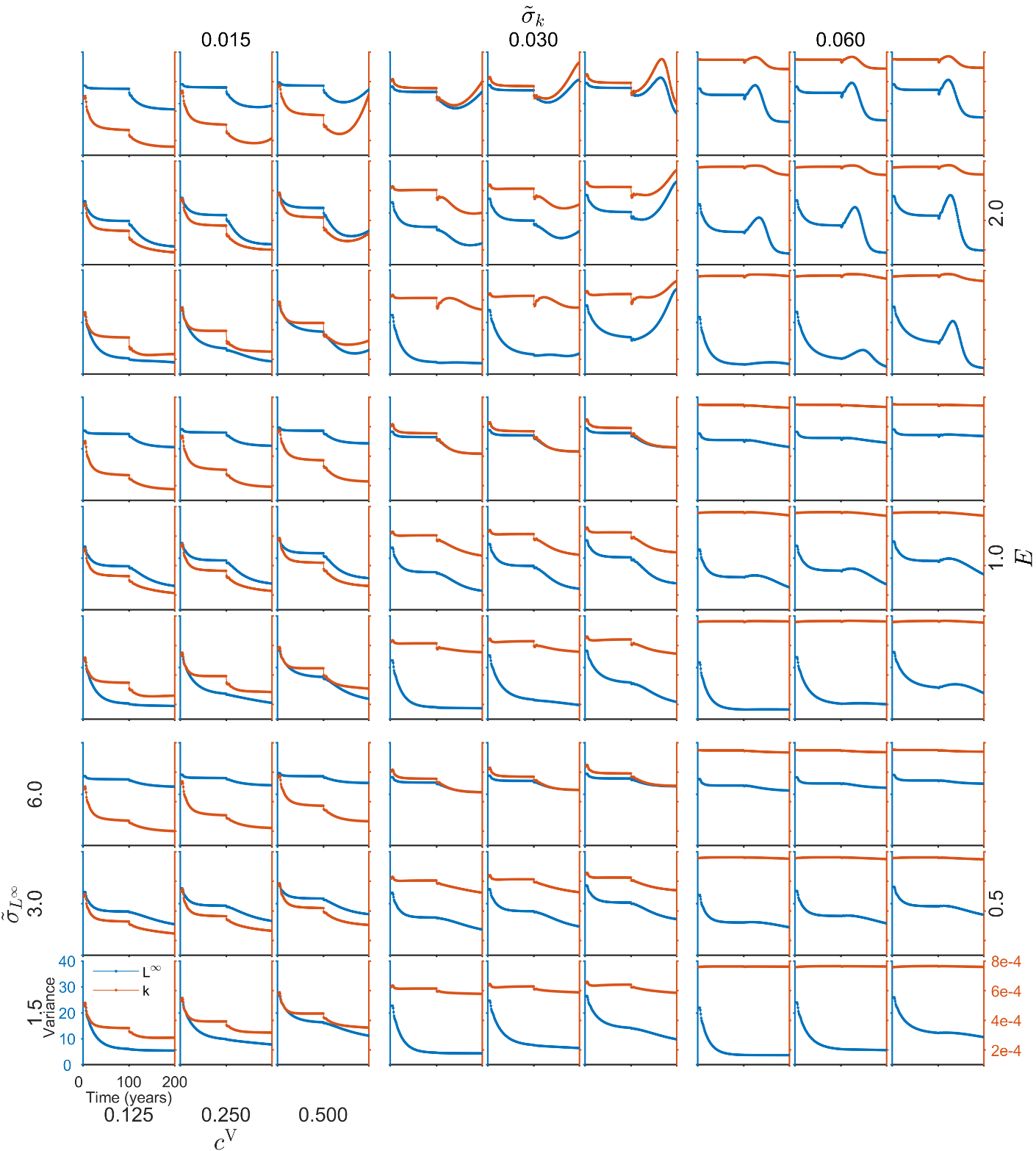
**

**Figure S27.** Variance in *Sander lucioperca* asymptotic length $L^{\infty}$ (blue) and Brody´s growth coefficient $k$(red) with different instantaneous fishing mortalities (E): $0.5y^{-1}$, $1.0 y^{-1}$and $2.0y^{-1}$, phenotypic variance parameters $\tilde{\sigma}_{L^{\infty}}$ values: 1.5 cm, 3.0 cm and 6.0 cm, $\tilde{\sigma}_{k}$ values: 0.015 $y^{-1}$, 0.030 $y^{-1}$ and 0.060 $y^{-1}$ and genotypic variance parameter $c^{V}$ values: 0.125, 0.250 and 0.500, with a high ($\rho$ = -0.7) level of correlation between $k$ and $L^{\infty}$.


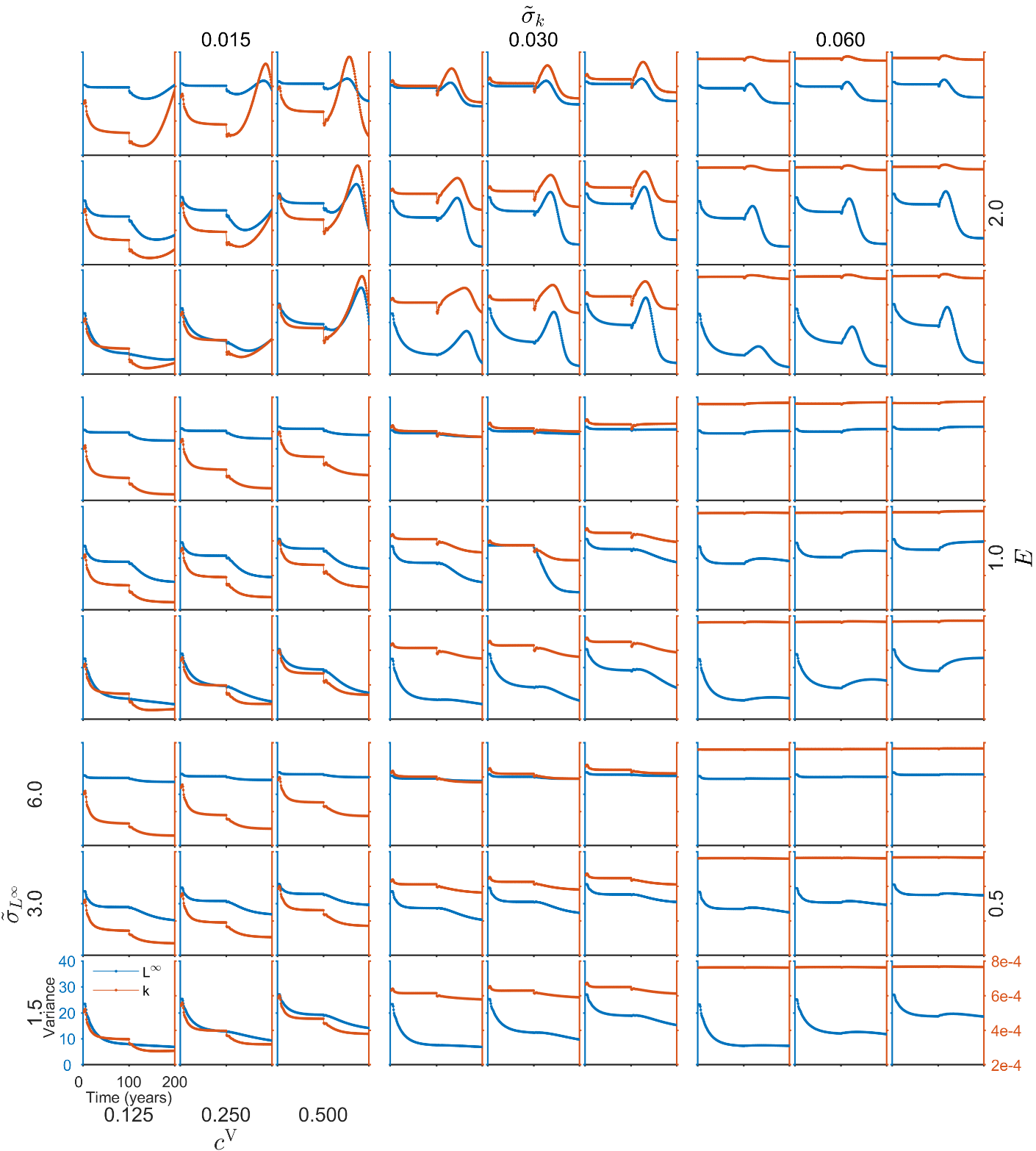


**Figure S28.** Variance in *Sander lucioperca* asymptotic length $L^{\infty}$ (blue) and Brody´s growth coefficient $k$(red) with different instantaneous fishing mortalities (E): $0.5y^{-1}$, $1.0 y^{-1}$and $2.0y^{-1}$, phenotypic variance parameters $\tilde{\sigma}_{L^{\infty}}$ values: 1.5 cm, 3.0 cm and 6.0 cm, $\tilde{\sigma}_{k}$ values: 0.015 $y^{-1}$, 0.030 $y^{-1}$ and 0.060 $y^{-1}$ and genotypic variance parameter $c^{V}$ values: 0.125, 0.250 and 0.500, with a low ($\rho$ = -0.35) level of correlation between $k$ and $L^{\infty}$.

**
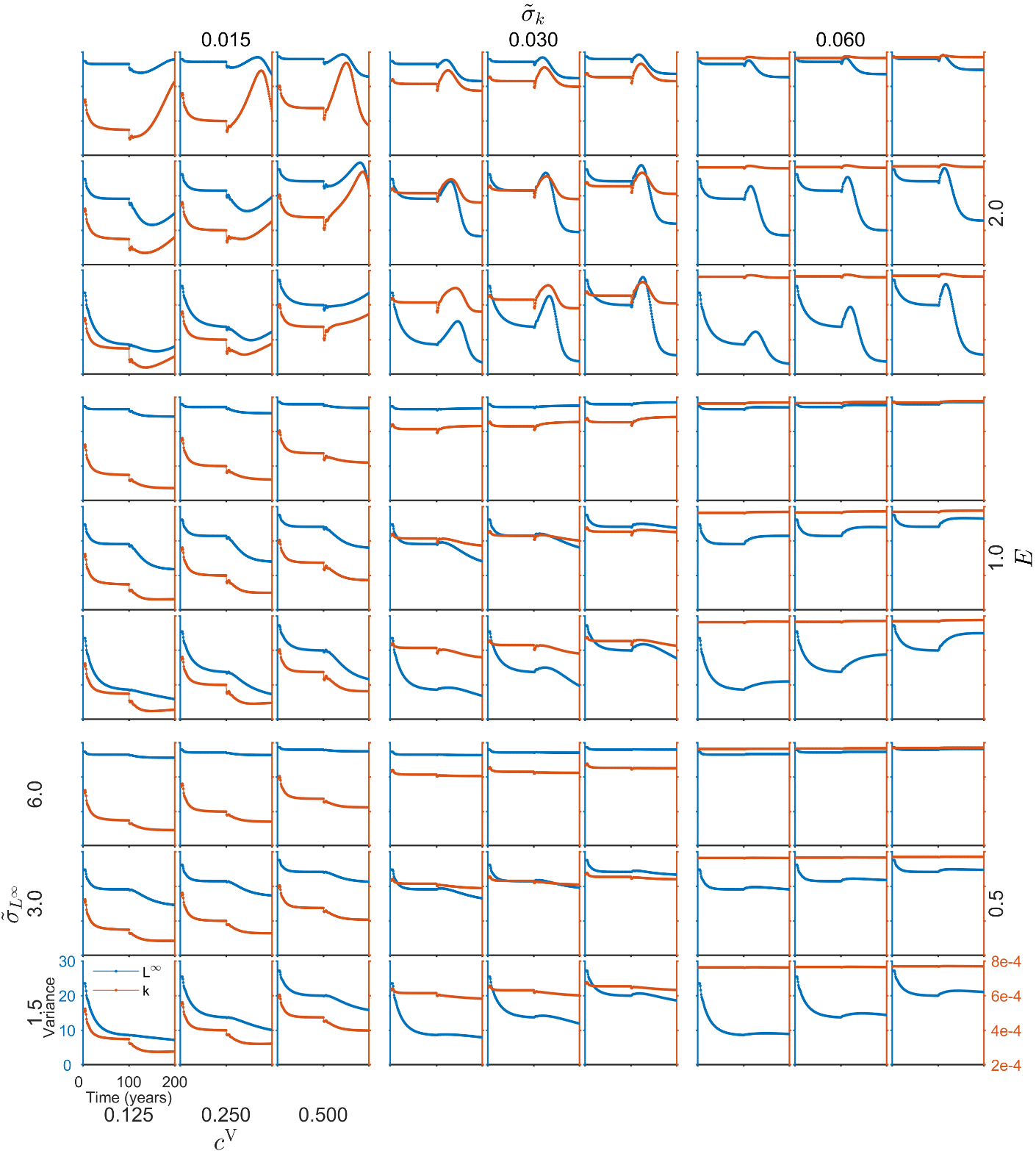
**

**Figure S29.** Variance in *Sander lucioperca* asymptotic length $L^{\infty}$ (blue) and Brody´s growth coefficient $k$(red) with different instantaneous fishing mortalities (E): $0.5y^{-1}$, $1.0 y^{-1}$and $2.0y^{-1}$, phenotypic variance parameters $\tilde{\sigma}_{L^{\infty}}$ values: 1.5 cm, 3.0 cm and 6.0 cm, $\tilde{\sigma}_{k}$ values: 0.015 $y^{-1}$, 0.030 $y^{-1}$ and 0.060 $y^{-1}$ and genotypic variance parameter $c^{V}$ values: 0.125, 0.250 and 0.500, with no correlation ($\rho$ = 0) between $k$ and $L^{\infty}$.


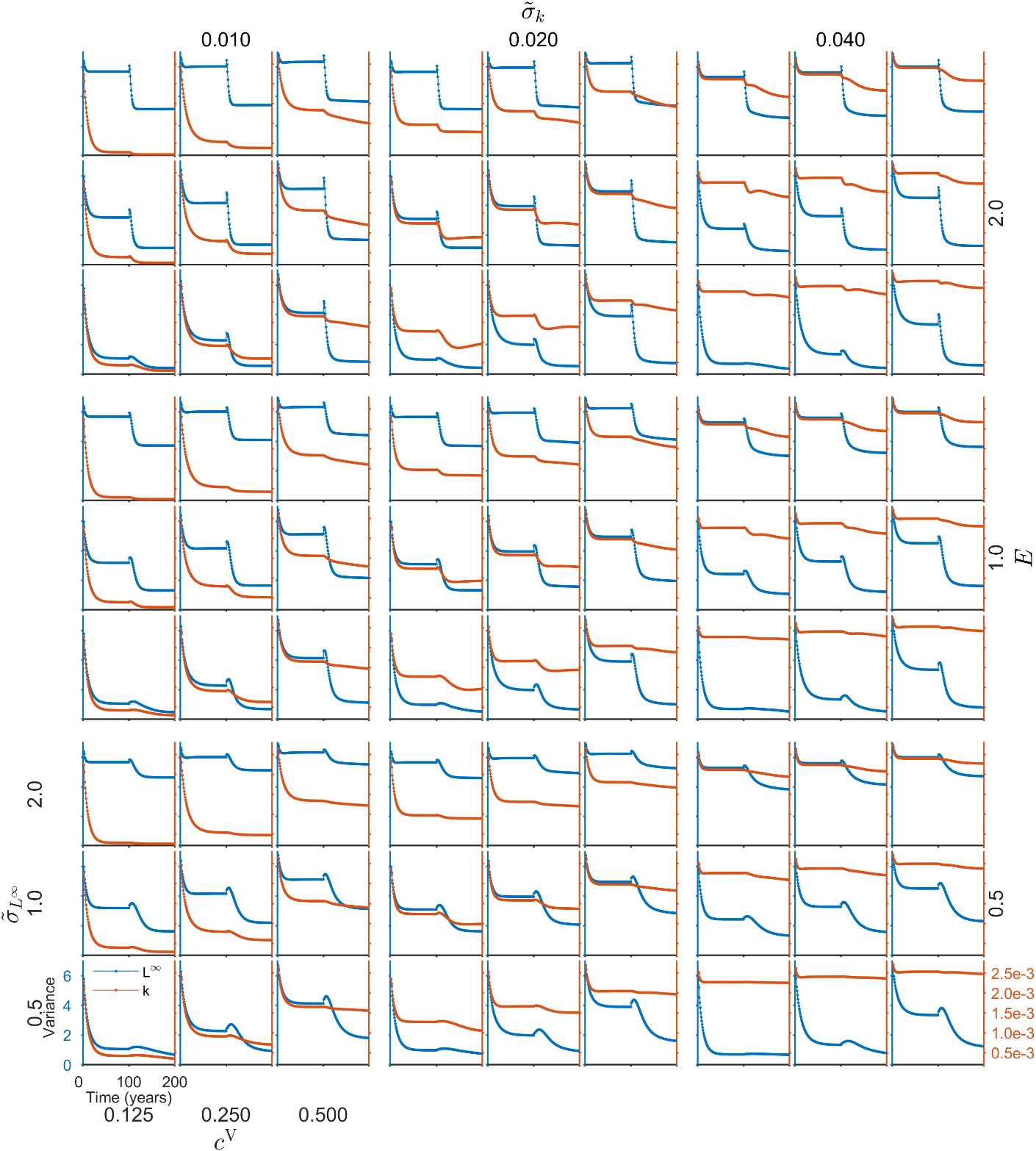


**Figure S30.** Variance in *Coregonus albula* asymptotic length $L^{\infty}$ (blue) and Brody´s growth coefficient $k$ (red) with different instantaneous fishing mortalities (E): $0.5y^{-1}$, $1.0y^{-1}$and $2.0y^{-1}$, phenotypic variance parameters $\tilde{\sigma}_{L^{\infty}}$ values: 0.5 cm, 1.0 cm and 2.0 cm, $\tilde{\sigma}_{k}$ values: 0.010 $y^{-1}$, 0.020 $y^{-1}$ and 0.040 $y^{-1}$ and genotypic variance parameter $c^{V}$ values: 0.125, 0.250 and 0.500, with a high ($\rho$ = -0.7) level of correlation between $k$ and $L^{\infty}$.


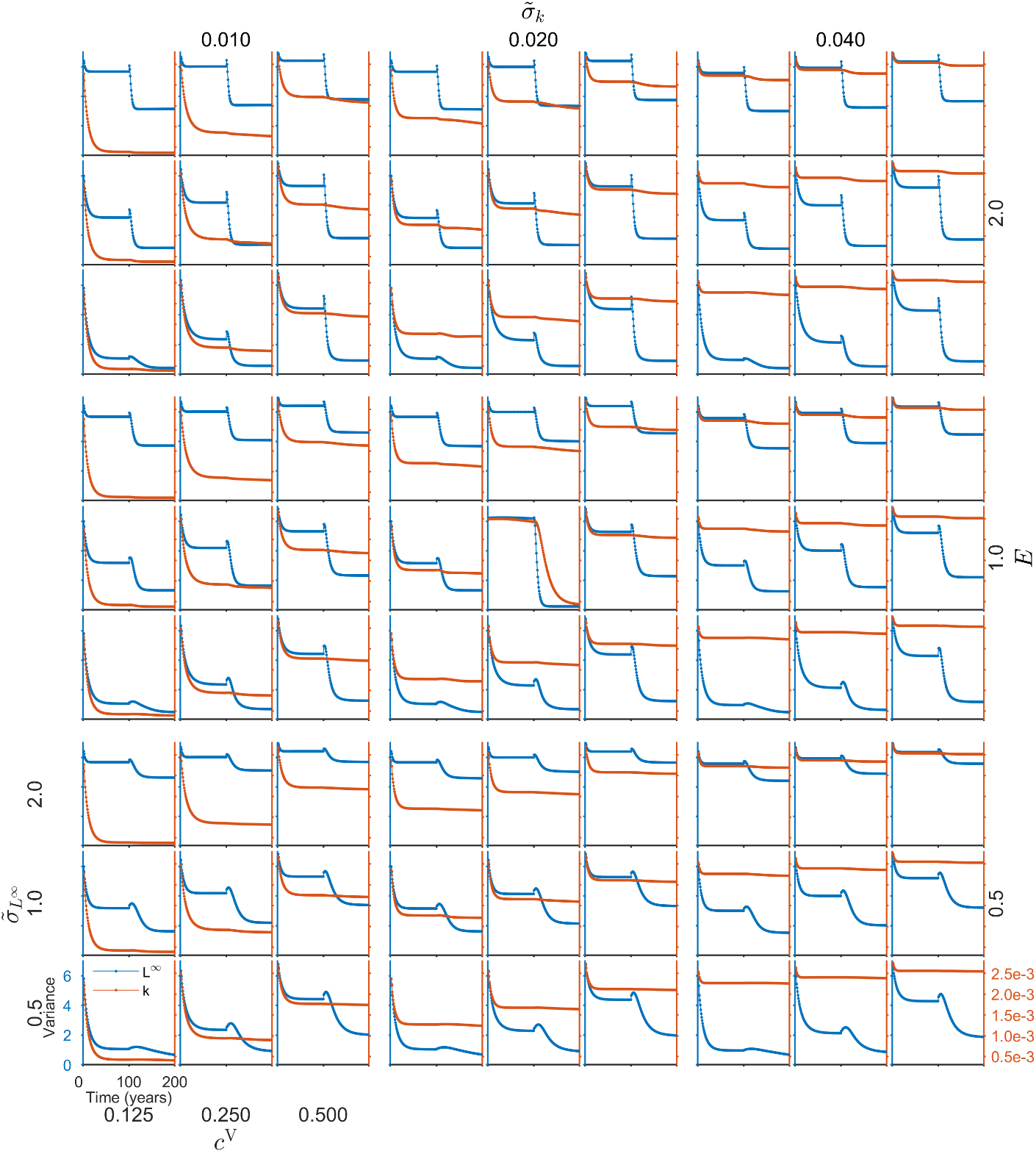


**Figure S31.** Variance in *Coregonus albula* asymptotic length $L^{\infty}$ (blue) and Brody´s growth coefficient $k$ (red) with different instantaneous fishing mortalities (E): $0.5y^{-1}$, $1.0y^{-1}$and $2.0y^{-1}$, phenotypic variance parameters $\tilde{\sigma}_{L^{\infty}}$ values: 0.5 cm, 1.0 cm and 2.0 cm, $\tilde{\sigma}_{k}$ values: 0.010 $y^{-1}$, 0.020 $y^{-1}$ and 0.040 $y^{-1}$ and genotypic variance parameter $c^{V}$ values: 0.125, 0.250 and 0.500, with a low ($\rho$ = -0.35) level of correlation between $k$ and $L^{\infty}$.


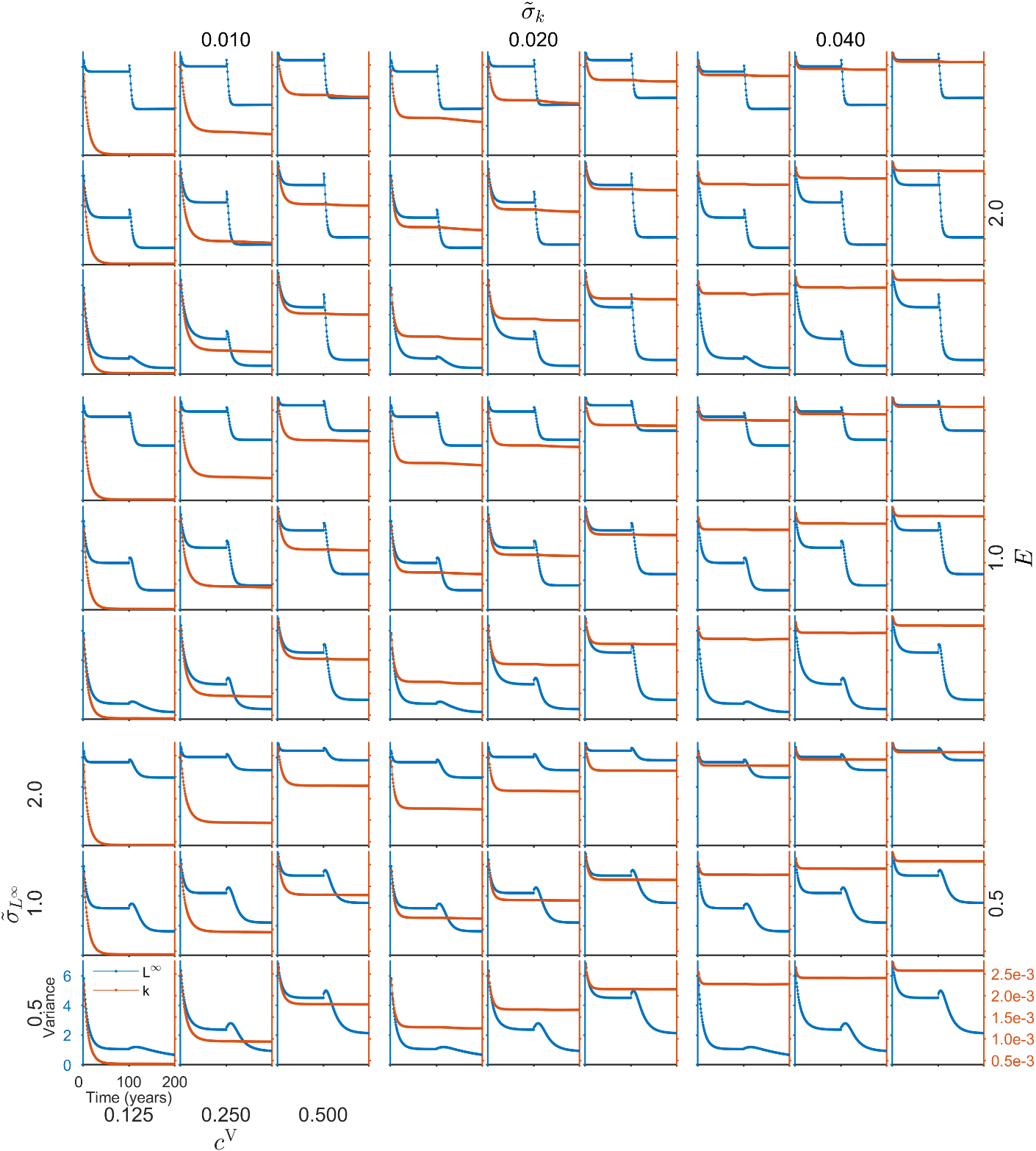


**Figure S32.** Variance in *Coregonus albula* asymptotic length $L^{\infty}$ (blue) and Brody´s growth coefficient $k$ (red) with different instantaneous fishing mortalities (E): $0.5y^{-1}$, $1.0y^{-1}$and $2.0y^{-1}$, phenotypic variance parameters $\tilde{\sigma}_{L^{\infty}}$ values: 0.5 cm, 1.0 cm and 2.0 cm, $\tilde{\sigma}_{k}$ values: 0.010 $y^{-1}$, 0.020 $y^{-1}$ and 0.040 $y^{-1}$ and genotypic variance parameter $c^{V}$ values: 0.125, 0.250 and 0.500, with no correlation ($\rho$ = 0) between $k$ and $L^{\infty}$.

**SUPPLEMENTARY RESULTS – BIOMASSES**

**
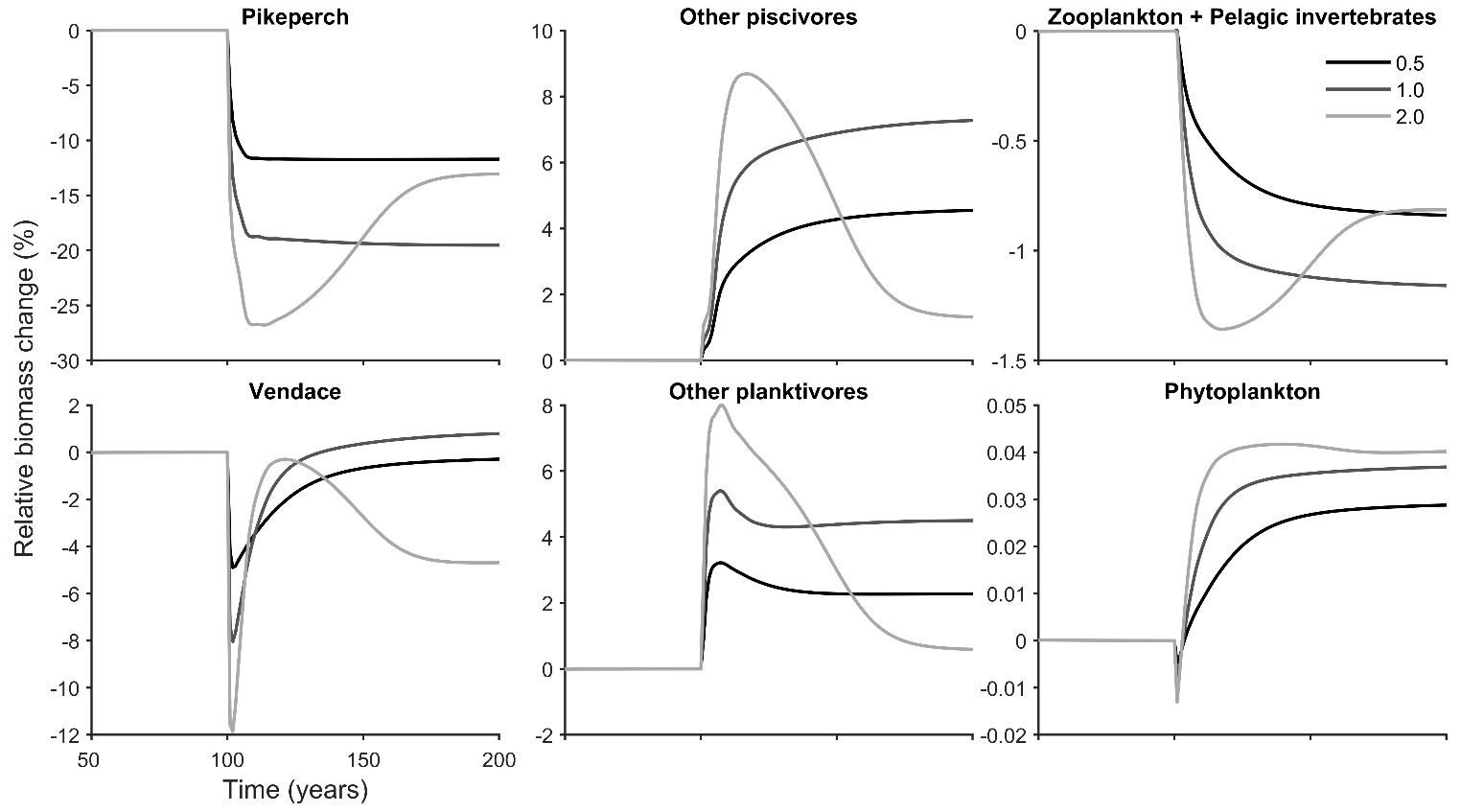
****Figure S33.** Relative biomass change (%) in various ecological components over a range of instantaneous fishing mortalities ($E = 0.5y^{-1}, 1.0y^{-1}, \mathrm{and} 2.0 y^{-1}$), with a low ($\rho$ = -0.35) level of correlation between $k$ (Brody’s growth coefficient) and $L^{\infty}$ (asymptotic length) for *Sander lucioperca* and *Coregonus albula*. The components include *Sander lucioperca*, other piscivores (*Salmo trutta* and *Perca fluviatilis* age ≥ 4), *Coregonus albula*, other planktivores (*Perca fluviatilis* age <4, *Coregonus lavaretus*, and *Osmerus eperlanus*), zooplankton and pelagic invertebrates, and phytoplankton. The comparison is made between the unfished equilibrium situation and the situation with fishing.

**
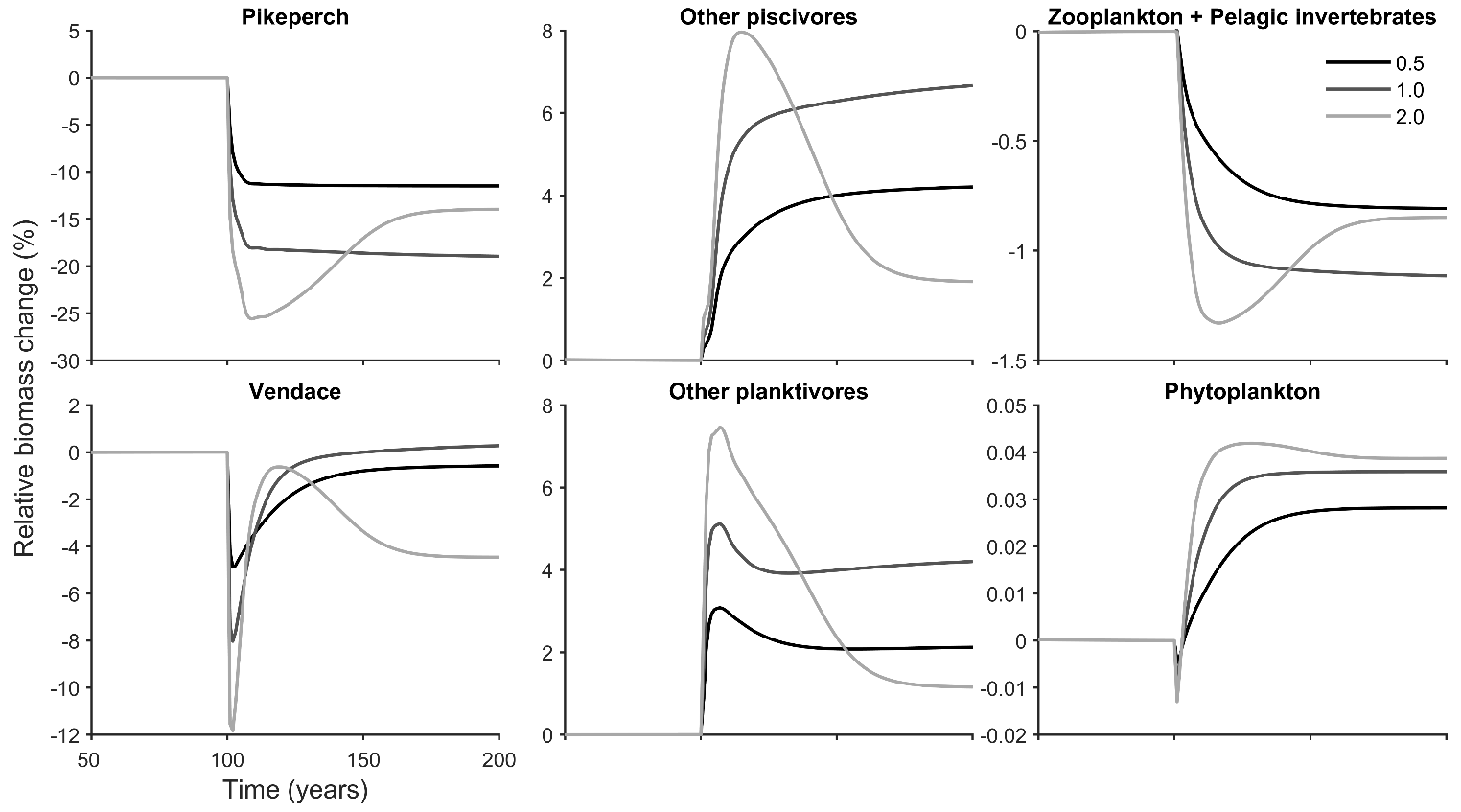
**

**Figure S34.** Relative biomass change (%) in various ecological components over a range of instantaneous fishing mortalities ($E = 0.5y^{-1}, 1.0y^{-1}, \mathrm{and} 2.0 y^{-1}$), with no correlation ($\rho$ = 0) between $k$ (Brody’s growth coefficient) and $L^{\infty}$ (asymptotic length) for *Sander lucioperca* and *Coregonus albula*. The components include *Sander lucioperca*, other piscivores (*Salmo trutta* and *Perca fluviatilis* age ≥ 4), *Coregonus albula*, other planktivores (*Perca fluviatilis* age <4, *Coregonus lavaretus*, and *Osmerus eperlanus*), zooplankton and pelagic invertebrates, and phytoplankton. The comparison is made between the unfished equilibrium situation and the situation with fishing.

**SUPPLEMENTARY RESULTS - BIOMASSES OF THE GUILDS**

**
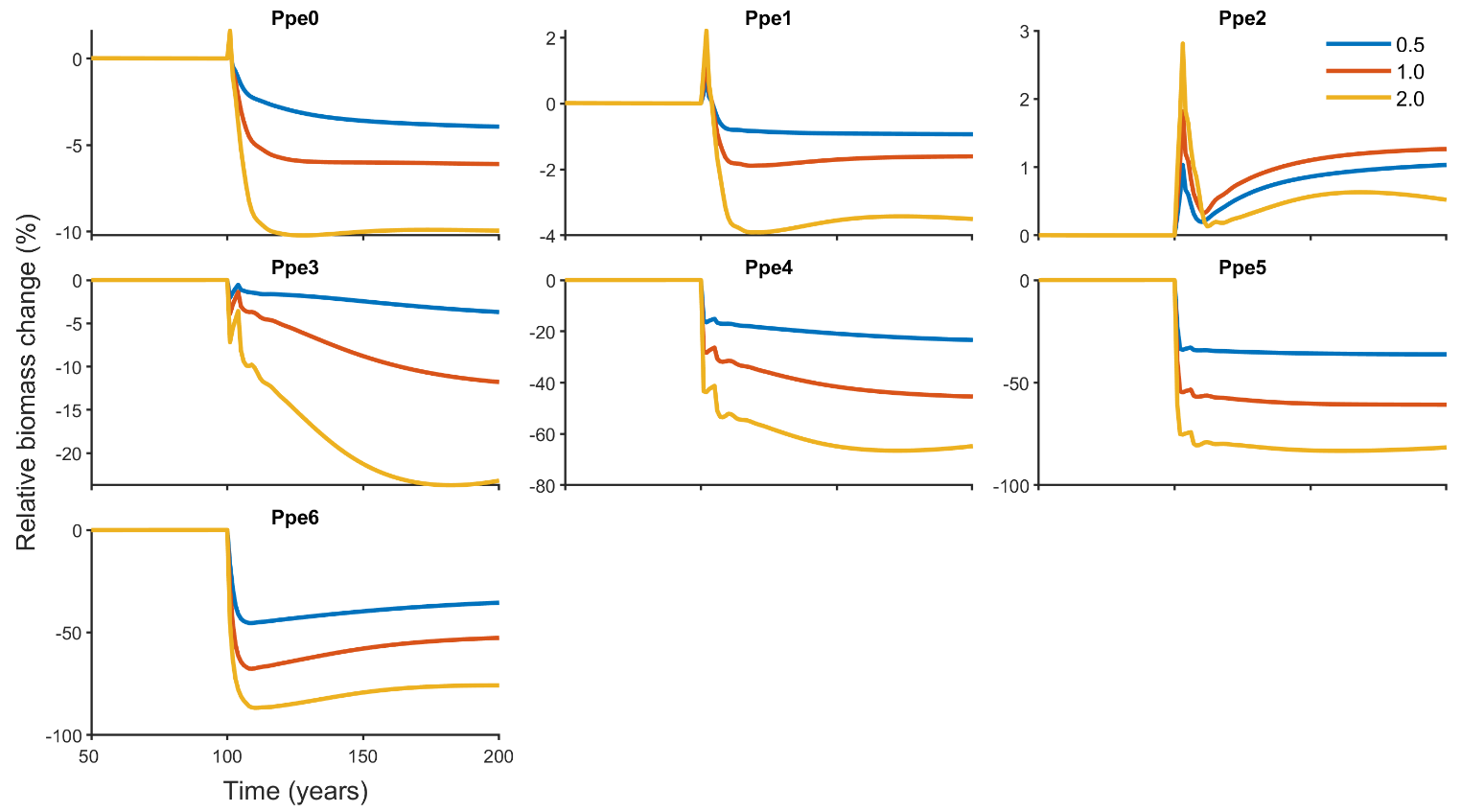
Figure S35.** Relative biomass change (%) in *Sander lucioperca* (Ppe) different age guilds, when comparing unfished equilibrium situation into situation with fishing with instantaneous fishing mortalities $0.5y^{-1}$, $1.0y^{-1}$or $2.0y^{-1}$, with a high ($\rho$ = -0.7) level of correlation between $k$ (Brody’s growth coefficient) and $L^{\infty}$ (asymptotic length) for *Sander lucioperca* and *Coregonus albula*. Ppe6 includes ages ≥6.


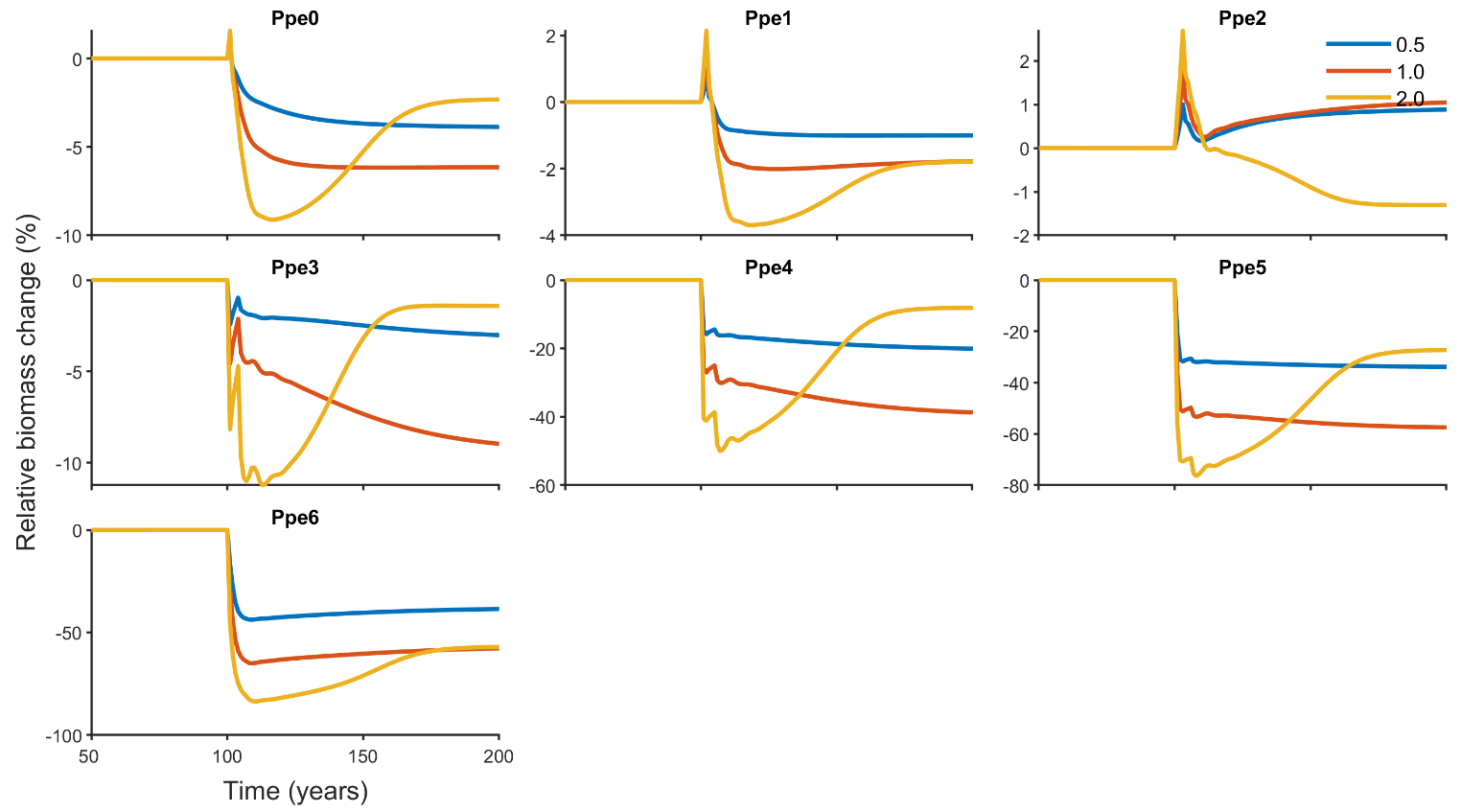
**Figure S36.** Relative biomass change (%) in *Sander lucioperca* (Ppe) different age guilds, when comparing unfished equilibrium situation into situation with fishing with instantaneous fishing mortalities $0.5y^{-1}$, $1.0y^{-1}$or $2.0y^{-1}$, with a low ($\rho$ = -0.35) level of correlation between $k$ (Brody’s growth coefficient) and $L^{\infty}$ (asymptotic length) for *Sander lucioperca* and *Coregonus albula*. Ppe6 includes ages ≥6.


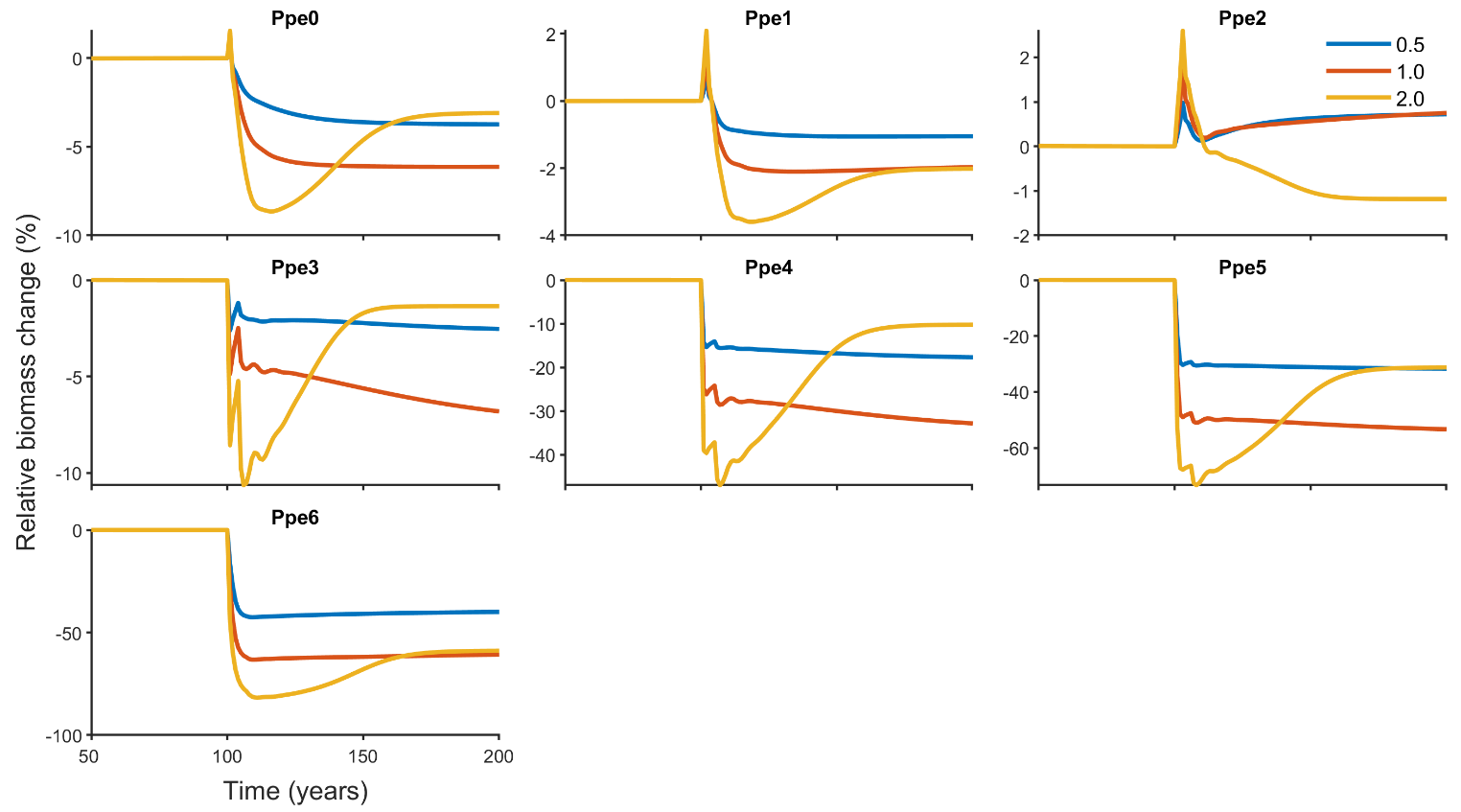


**Figure S37.** Relative biomass change (%) in *Sander lucioperca* (Ppe) different age guilds, when comparing unfished equilibrium situation into situation with fishing with instantaneous fishing mortalities $0.5y^{-1}$, $1.0y^{-1}$or $2.0y^{-1}$, with no correlation ($\rho$ = 0) between $k$ (Brody’s growth coefficient) and $L^{\infty}$ (asymptotic length) for *Sander lucioperca* and *Coregonus albula*. Ppe6 includes ages ≥6.


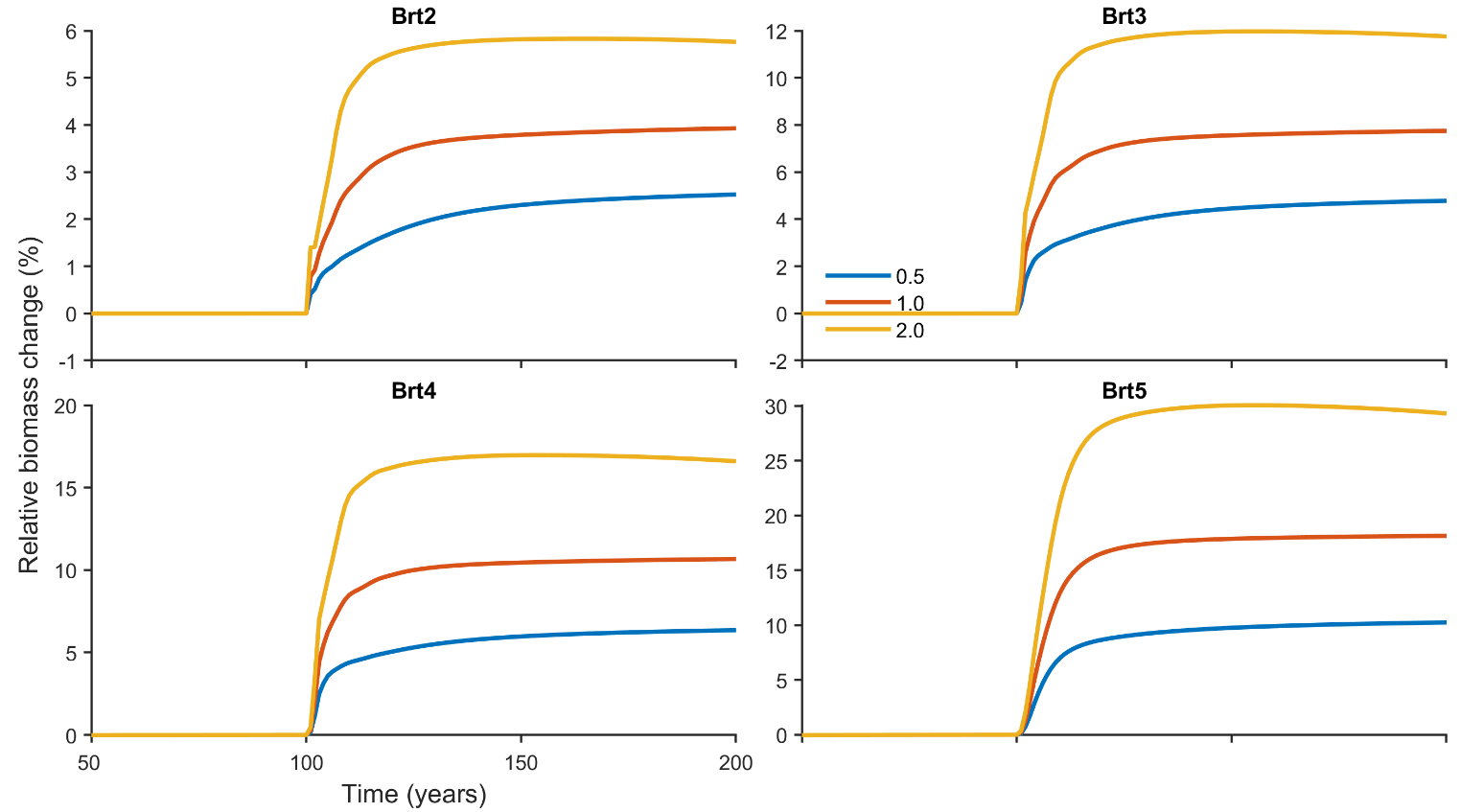
**Figure S38.** Relative biomass change (%) in *Salmo trutta* (Brt) different age guilds, when comparing unfished equilibrium situation into situation with fishing with instantaneous fishing mortalities $0.5y^{-1}$, $1.0y^{-1}$or $2.0y^{-1}$, with a high ($\rho$ = -0.7) level of correlation between $k$ (Brody’s growth coefficient) and $L^{\infty}$ (asymptotic length) for *Sander lucioperca* and *Coregonus albula*. Brt 5 includes ages ≥5.


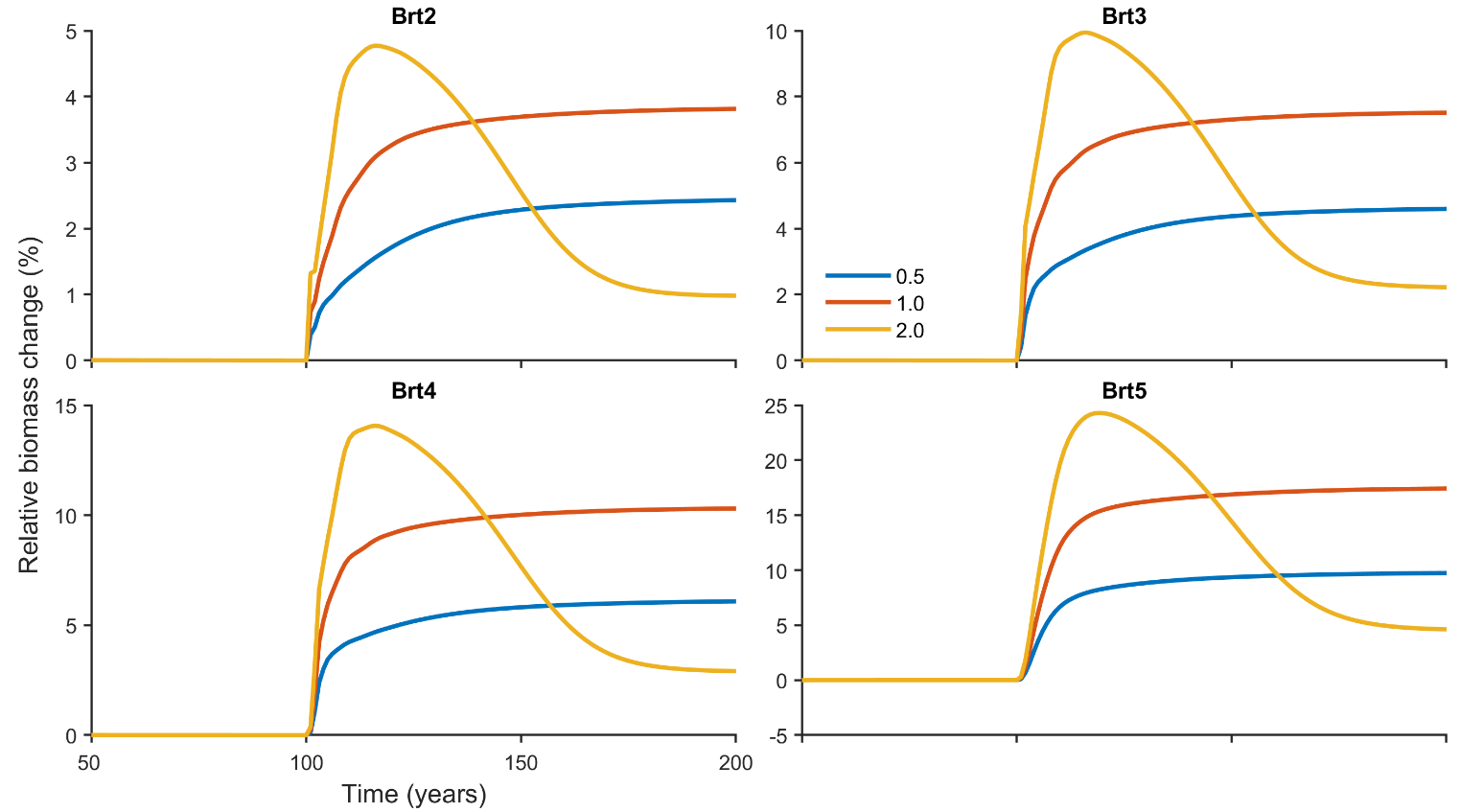
**Figure S39.** Relative biomass change (%) in *Salmo trutta* (Brt) different age guilds, when comparing unfished equilibrium situation into situation with fishing with instantaneous fishing mortalities $0.5y^{-1}$, $1.0y^{-1}$or $2.0y^{-1}$, with a low ($\rho$ = -0.35) level of correlation between $k$ (Brody’s growth coefficient) and $L^{\infty}$ (asymptotic length) for *Sander lucioperca* and *Coregonus albula*. Brt 5 includes ages ≥5.


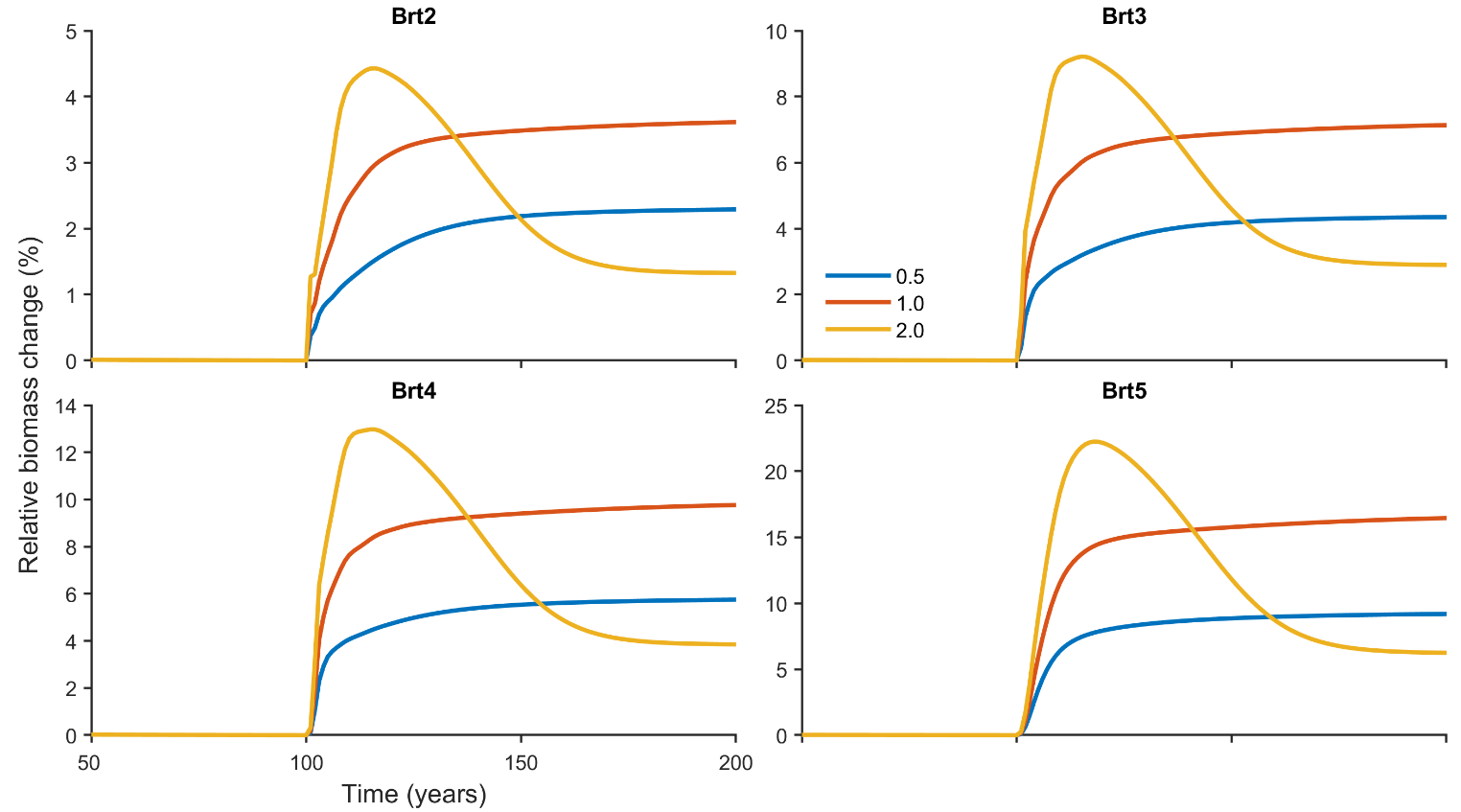


**Figure S40.** Relative biomass change (%) in *Salmo trutta* (Brt) different age guilds, when comparing unfished equilibrium situation into situation with fishing with instantaneous fishing mortalities $0.5y^{-1}$, $1.0y^{-1}$or $2.0y^{-1}$, with no correlation ($\rho$ = 0) between $k$ (Brody’s growth coefficient) and $L^{\infty}$ (asymptotic length) for *Sander lucioperca* and *Coregonus albula*. Brt 5 includes ages ≥5.


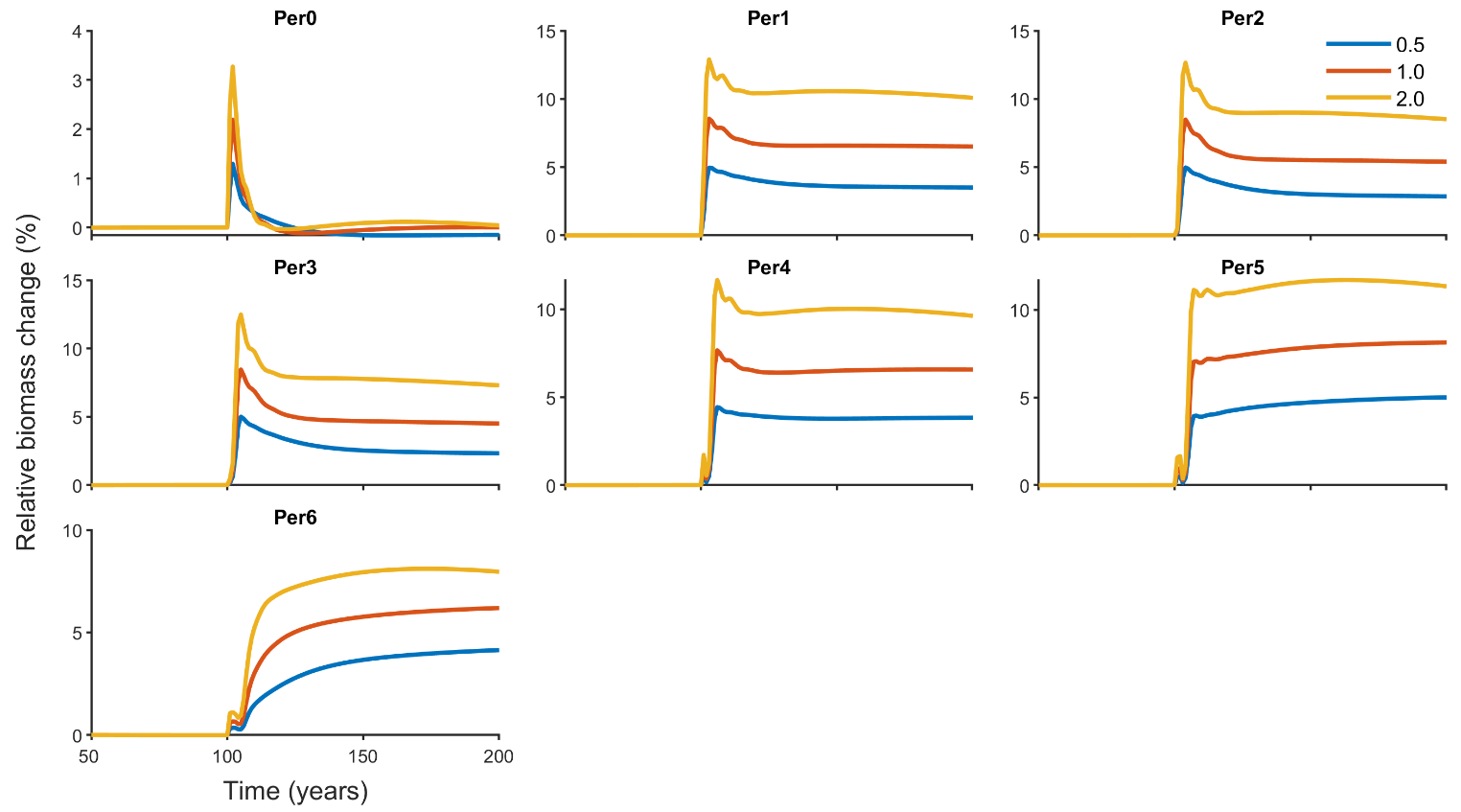
**Figure S41.** Relative biomass change (%) in *Perca fluviatilis* (Per) different age guilds, when comparing unfished equilibrium situation into situation with fishing with instantaneous fishing mortalities $0.5y^{-1}$, $1.0y^{-1}$or $2.0y^{-1}$, with a high ($\rho$ = -0.7) level of correlation between $k$ (Brody’s growth coefficient) and $L^{\infty}$ (asymptotic length) for *Sander lucioperca* and *Coregonus albula*. Per6 includes ages ≥6.


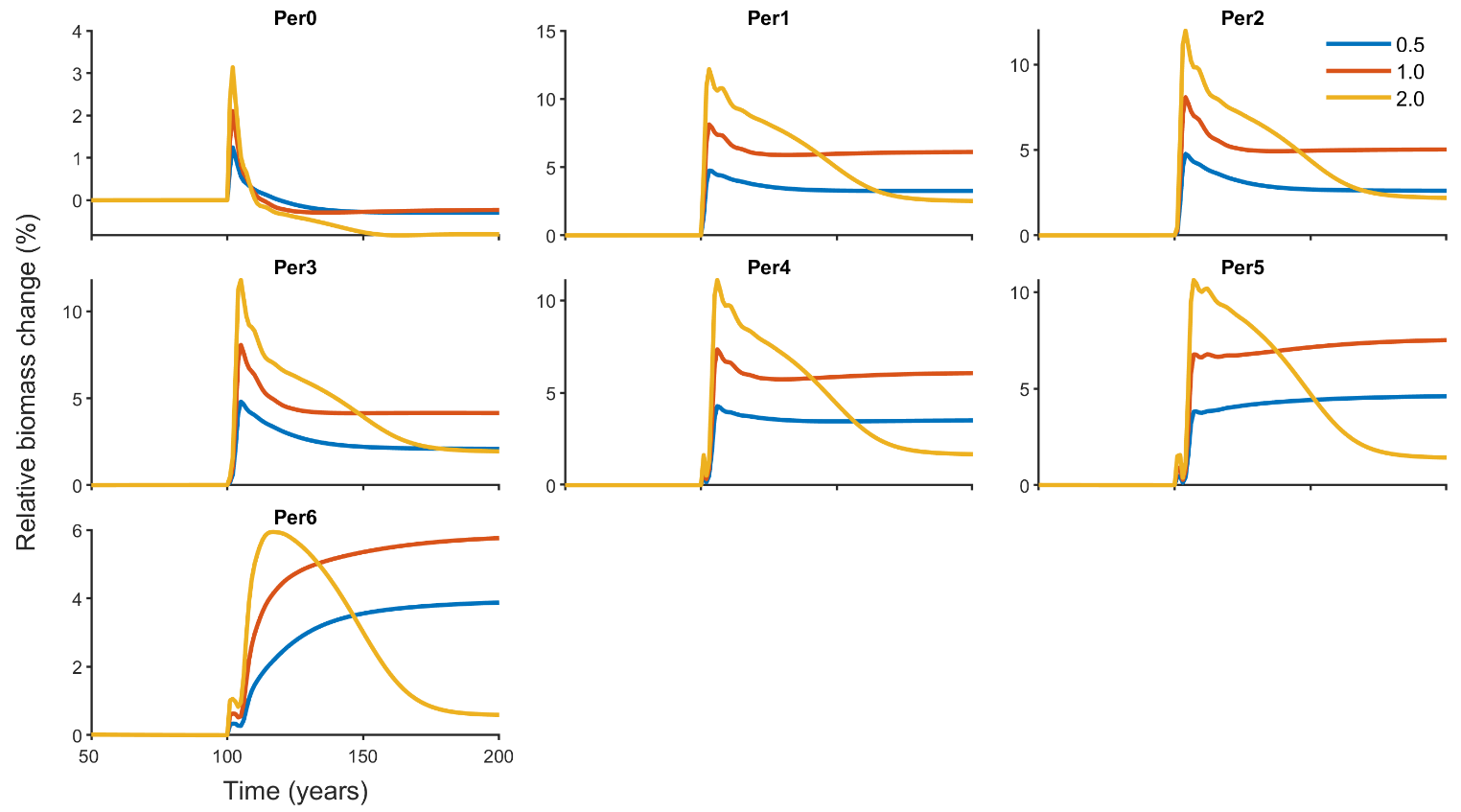
**Figure S42.** Relative biomass change (%) in *Perca fluviatilis* (Per) different age guilds, when comparing unfished equilibrium situation into situation with fishing with instantaneous fishing mortalities $0.5y^{-1}$, $1.0y^{-1}$or $2.0y^{-1}$, with a low ($\rho$ = -0.35) level of correlation between $k$ (Brody’s growth coefficient) and $L^{\infty}$ (asymptotic length) for *Sander lucioperca* and *Coregonus albula*. Per6 includes ages ≥6.


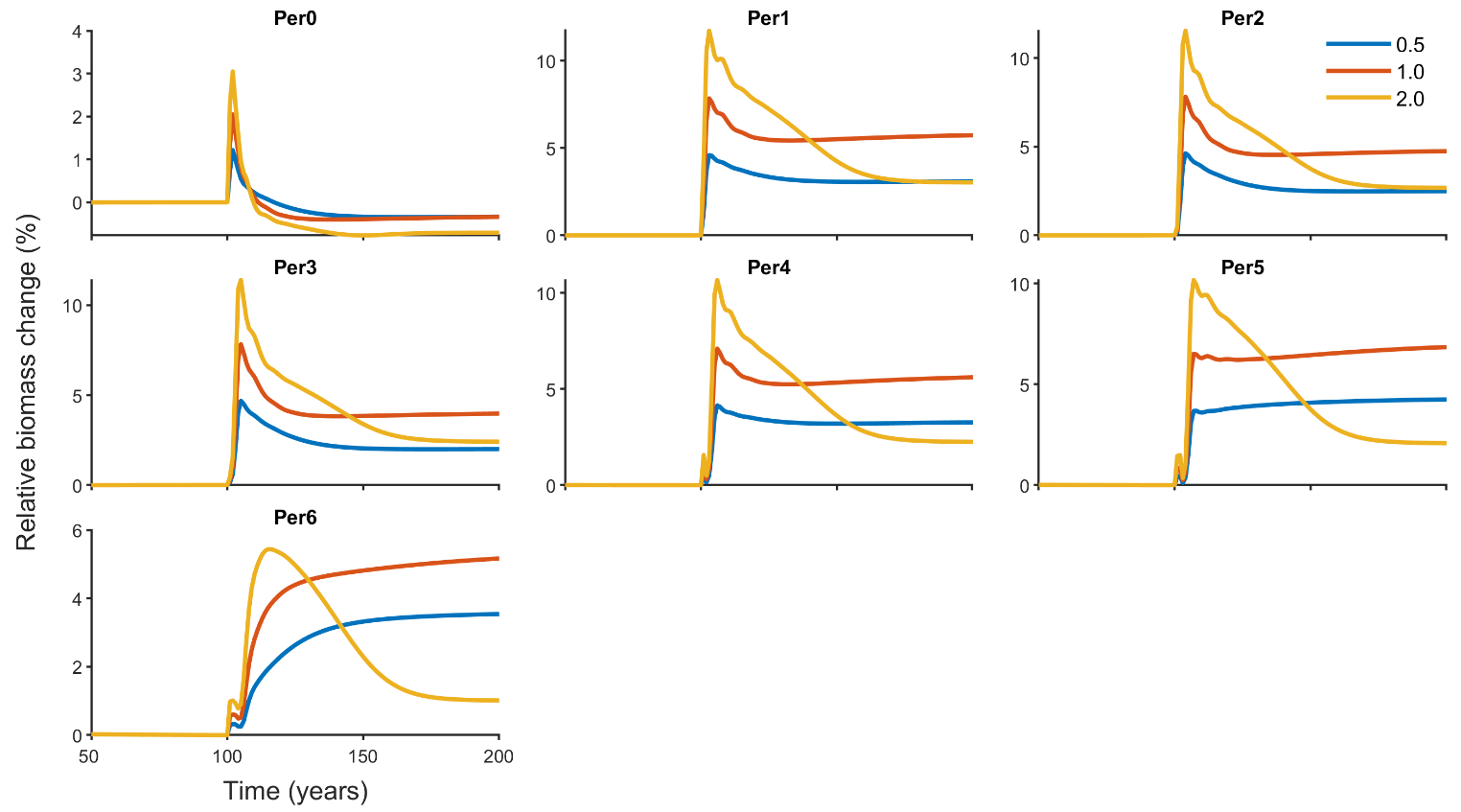


**Figure S43.** Relative biomass change (%) in *Perca fluviatilis* (Per) different age guilds, when comparing unfished equilibrium situation into situation with fishing with instantaneous fishing mortalities $0.5y^{-1}$, $1.0y^{-1}$or $2.0y^{-1}$, with no correlation ($\rho$ = 0) between $k$ (Brody’s growth coefficient) and $L^{\infty}$ (asymptotic length) for *Sander lucioperca* and *Coregonus albula*. Per6 includes ages ≥6.


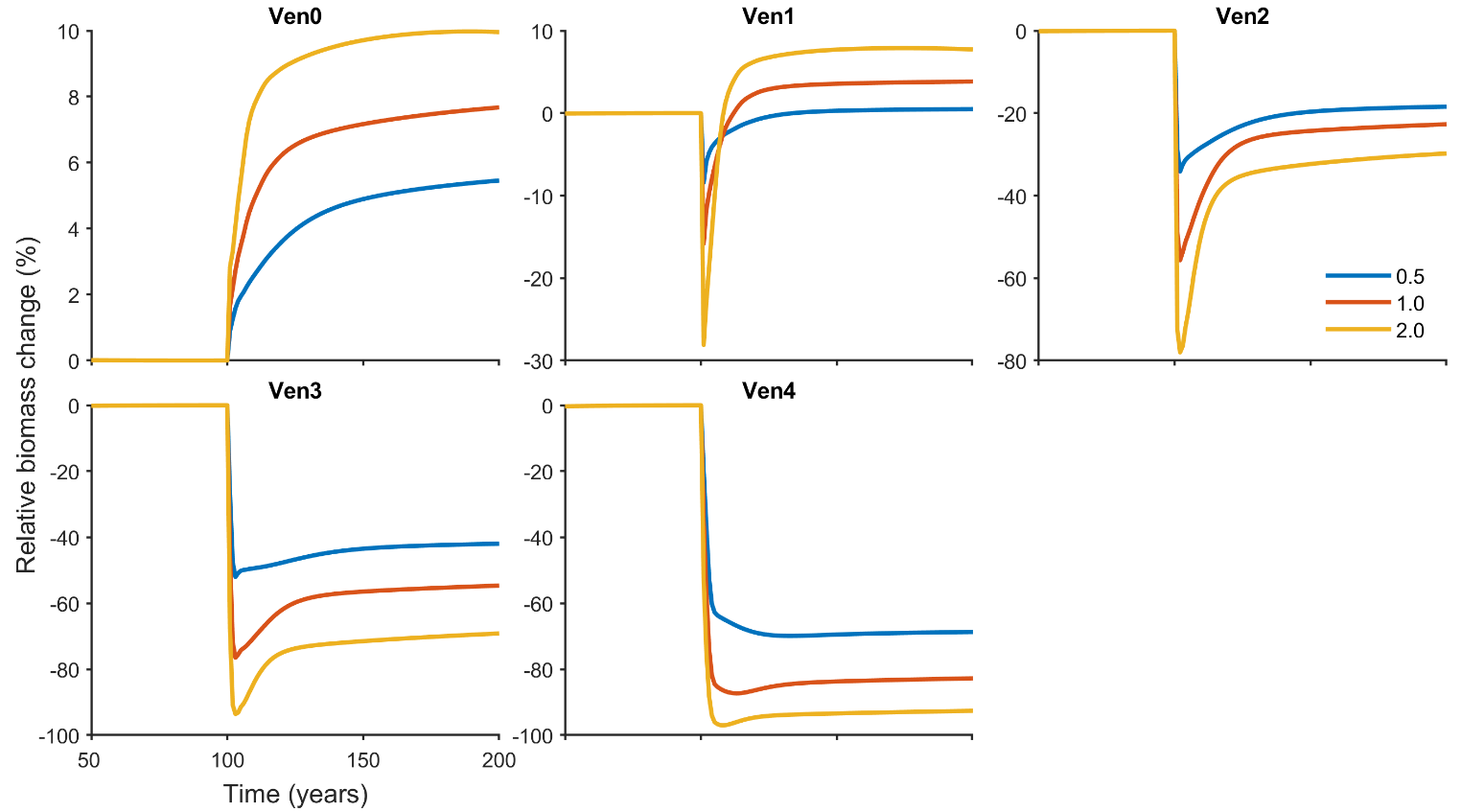
**Figure S44.** Relative biomass change (%) in *Coregonus albula* (Ven) different age guilds, when comparing unfished equilibrium situation into situation with fishing with instantaneous fishing mortalities $0.5y^{-1}$, $1.0y^{-1}$or $2.0y^{-1}$, with a high ($\rho$ = -0.7) level of correlation between $k$ (Brody’s growth coefficient) and $L^{\infty}$ (asymptotic length) for *Sander lucioperca* and *Coregonus albula*. Ven4 includes ages ≥4.


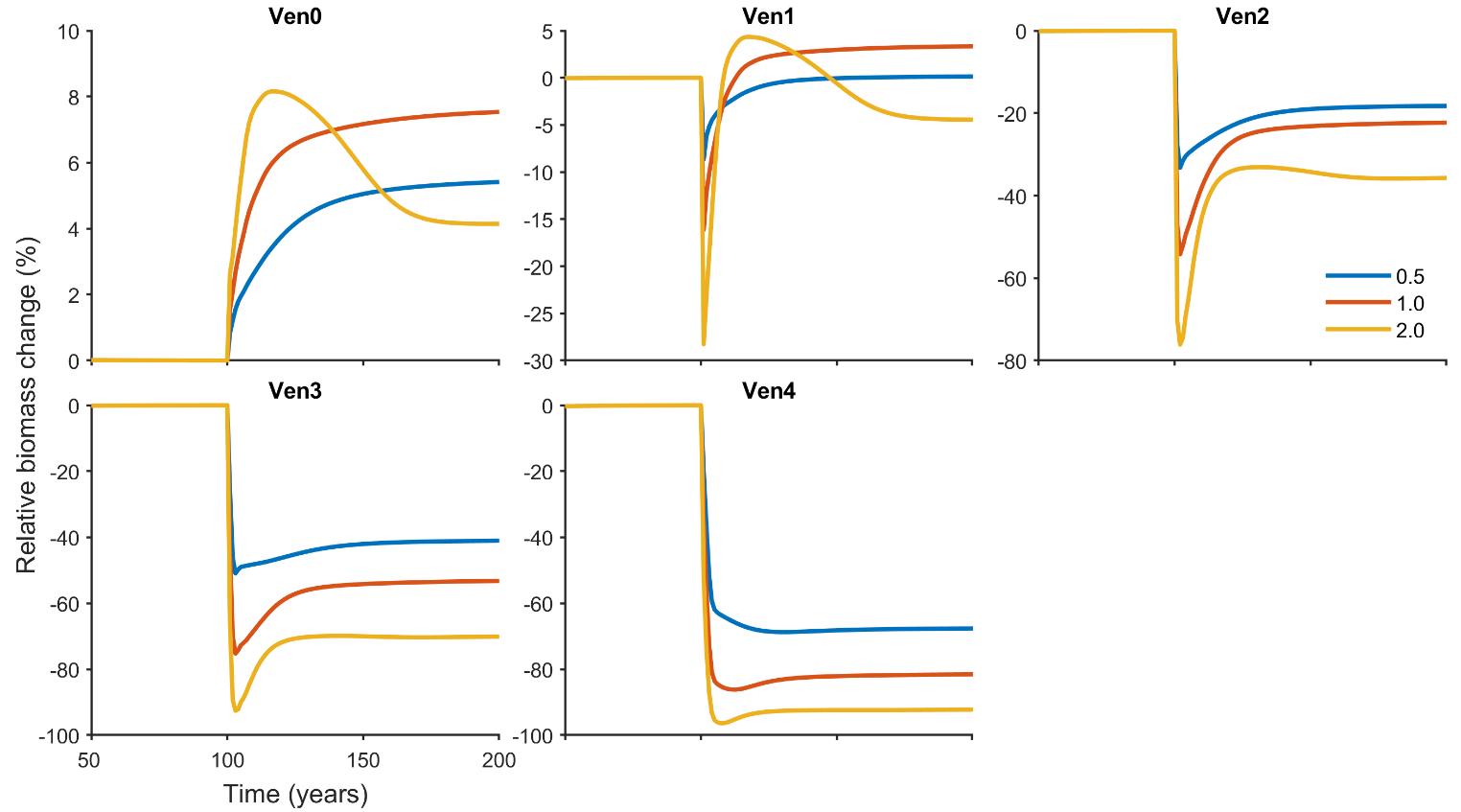
**Figure S45.** Relative biomass change (%) in *Coregonus albula* (Ven) different age guilds, when comparing unfished equilibrium situation into situation with fishing with instantaneous fishing mortalities $0.5y^{-1}$, $1.0y^{-1}$or $2.0y^{-1}$, with a low ($\rho$ = -0.35) level of correlation between $k$ (Brody’s growth coefficient) and $L^{\infty}$ (asymptotic length) for *Sander lucioperca* and *Coregonus albula*. Ven4 includes ages ≥4.


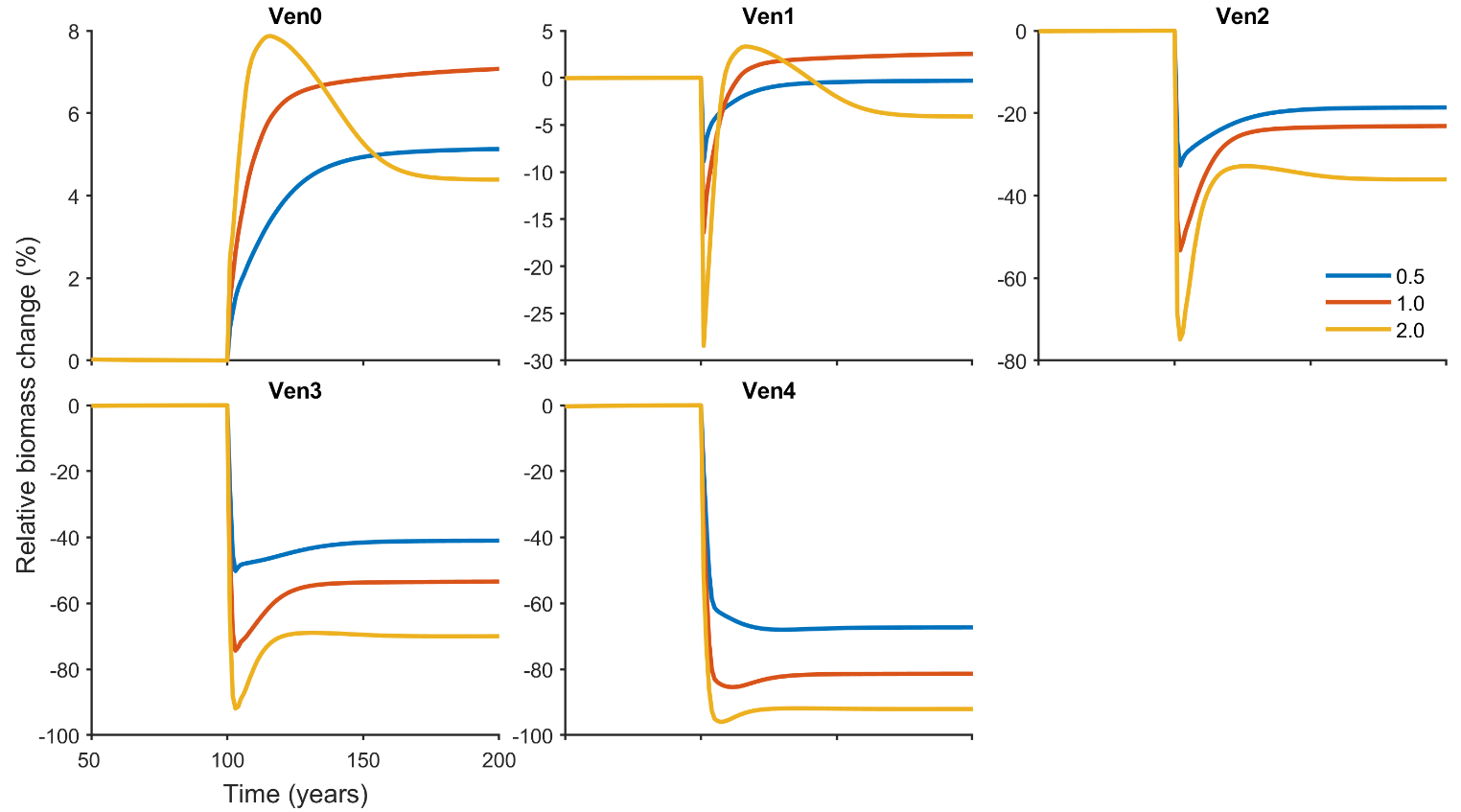


**Figure S46.** Relative biomass change (%) in *Coregonus albula* (Ven) different age guilds, when comparing unfished equilibrium situation into situation with fishing with instantaneous fishing mortalities $0.5y^{-1}$, $1.0y^{-1}$or $2.0y^{-1}$, with no correlation ($\rho$ = 0) between $k$ (Brody’s growth coefficient) and $L^{\infty}$ (asymptotic length) for *Sander lucioperca* and *Coregonus albula*. Ven4 includes ages ≥4.


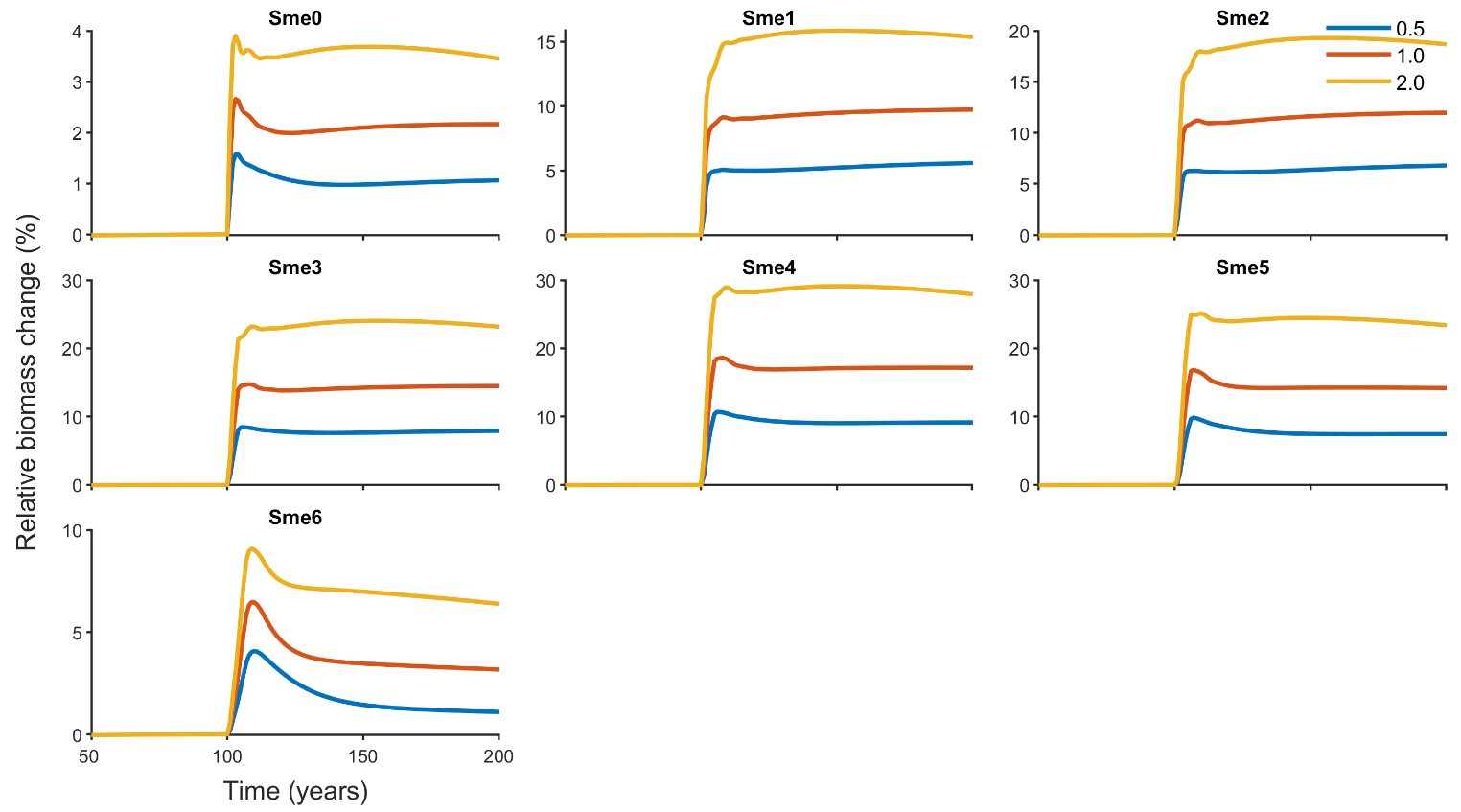
**Figure S47.** Relative biomass change (%) in *Osmerus eperlanus* (Sme) different age guilds, when comparing unfished equilibrium situation into situation with fishing with instantaneous fishing mortalities $0.5y^{-1}$, $1.0y^{-1}$or $2.0y^{-1}$, with a high ($\rho$ = -0.7) level of correlation between $k$ (Brody’s growth coefficient) and $L^{\infty}$ (asymptotic length) for *Sander lucioperca* and *Coregonus albula*. Sme6 includes ages ≥6.


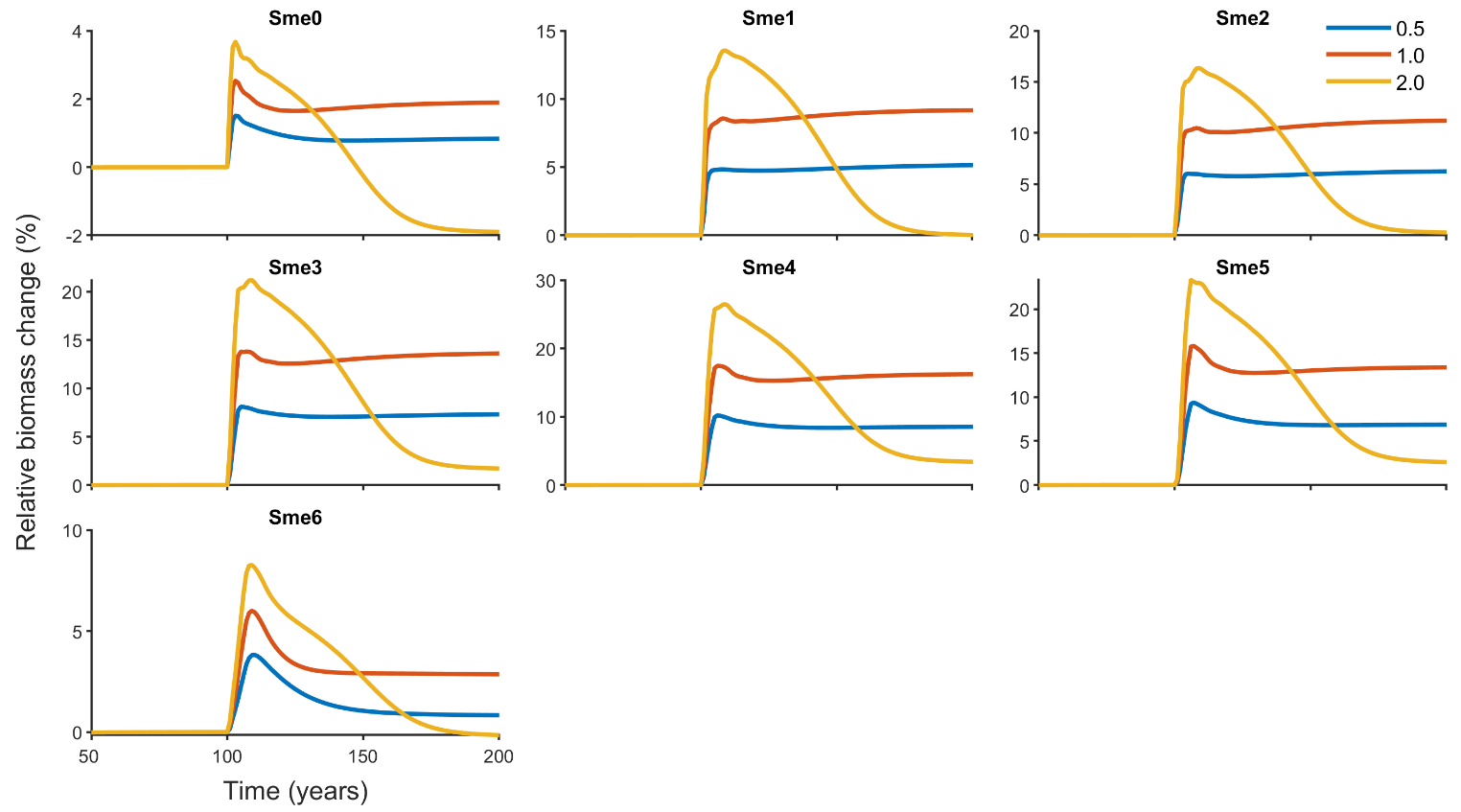
**Figure S48.** Relative biomass change (%) in *Osmerus eperlanus* (Sme) different age guilds, when comparing unfished equilibrium situation into situation with fishing with instantaneous fishing mortalities $0.5y^{-1}$, $1.0y^{-1}$or $2.0y^{-1}$, with a low ($\rho$ = -0.35) level of correlation between $k$ (Brody’s growth coefficient) and $L^{\infty}$ (asymptotic length) for *Sander lucioperca* and *Coregonus albula*. Sme6 includes ages ≥6.


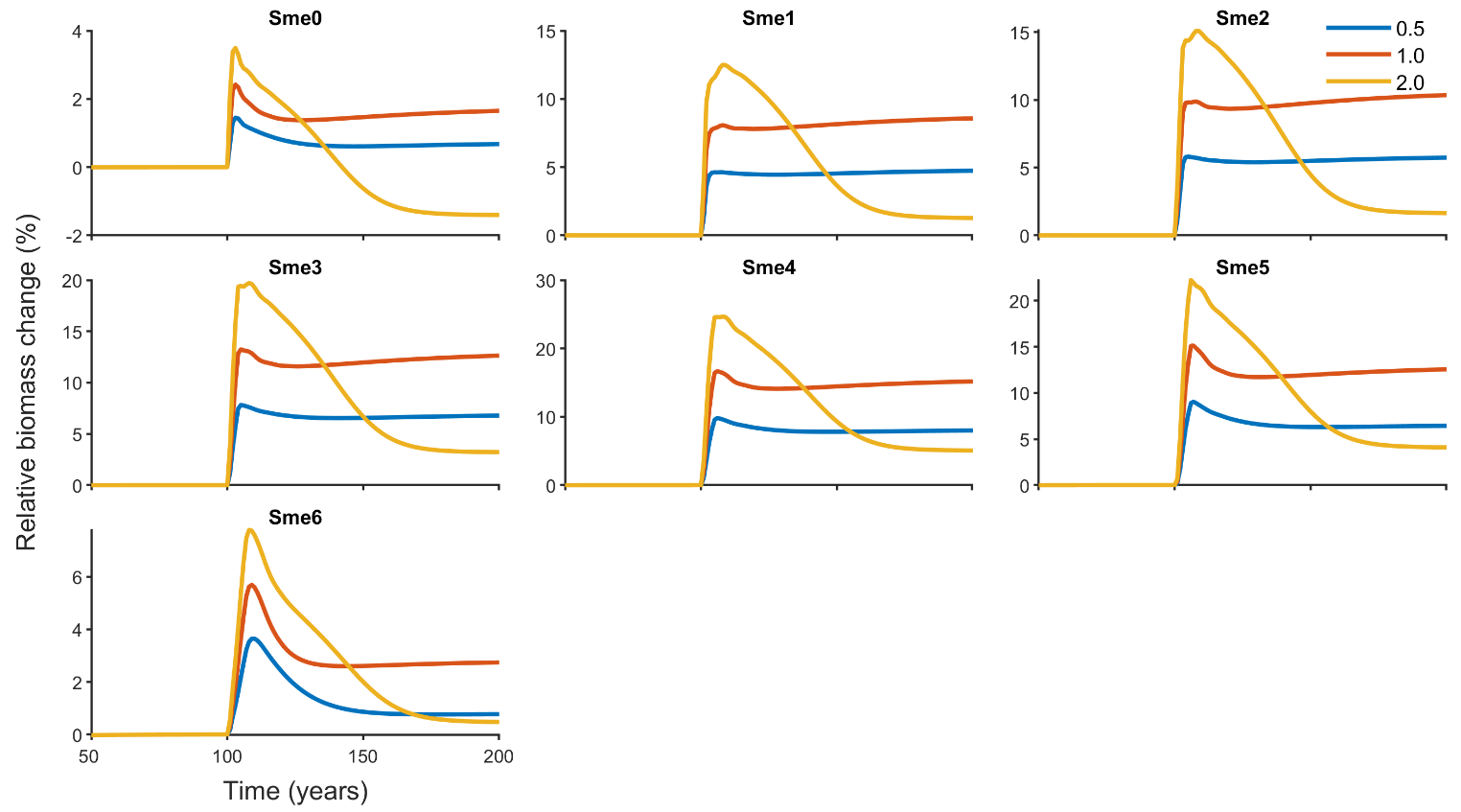


**Figure S49.** Relative biomass change (%) in *Osmerus eperlanus* (Sme) different age guilds, when comparing unfished equilibrium situation into situation with fishing with instantaneous fishing mortalities $0.5y^{-1}$, $1.0y^{-1}$or $2.0y^{-1}$, with no correlation ($\rho$ = 0) between $k$ (Brody’s growth coefficient) and $L^{\infty}$ (asymptotic length) for *Sander lucioperca* and *Coregonus albula*. Sme6 includes ages ≥6.


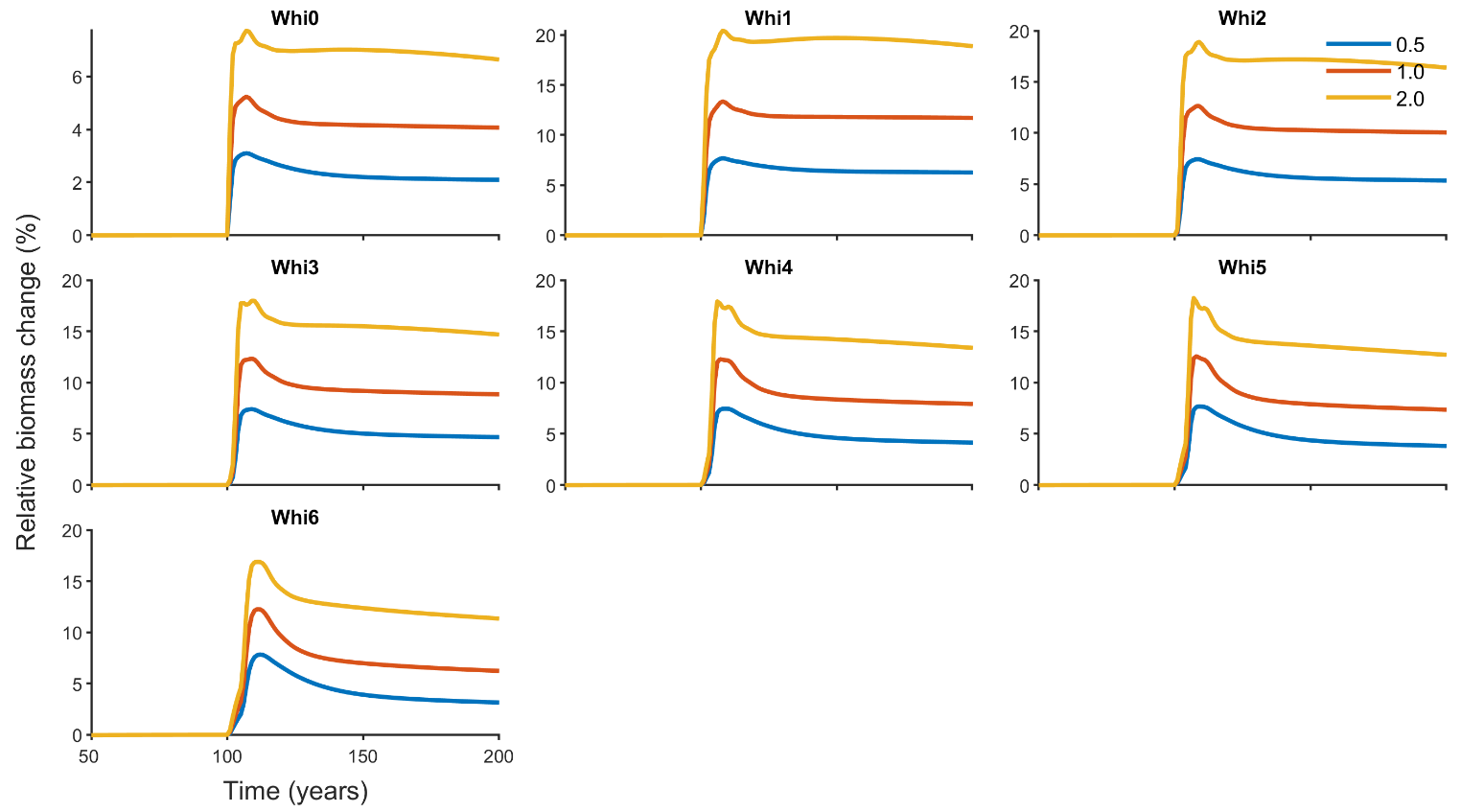
**Figure S50.** Relative biomass change (%) in *Coregonus lavaretus* (Whi) different age guilds, when comparing unfished equilibrium situation into situation with fishing with instantaneous fishing mortalities $0.5y^{-1}$, $1.0y^{-1}$or $2.0y^{-1}$, with a high ($\rho$ = -0.7) level of correlation between $k$ (Brody’s growth coefficient) and $L^{\infty}$ (asymptotic length) for *Sander lucioperca* and *Coregonus albula*. Whi6 includes ages ≥6.


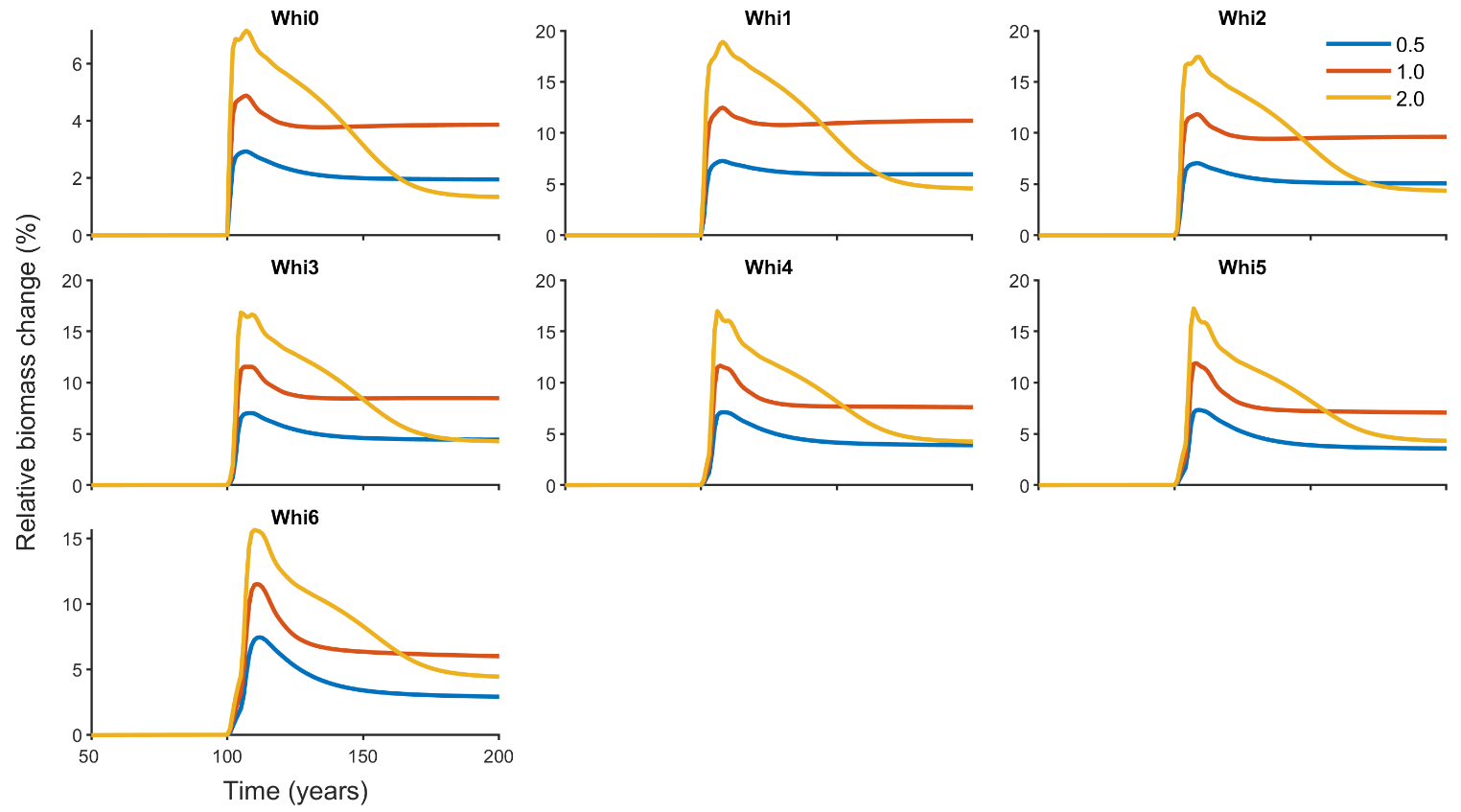
**Figure S51.** Relative biomass change (%) in *Coregonus lavaretus* (Whi) different age guilds, when comparing unfished equilibrium situation into situation with fishing with instantaneous fishing mortalities $0.5y^{-1}$, $1.0y^{-1}$or $2.0y^{-1}$, with a low ($\rho$ = -0.35) level of correlation between $k$ (Brody’s growth coefficient) and $L^{\infty}$ (asymptotic length) for *Sander lucioperca* and *Coregonus albula*. Whi6 includes ages ≥6.


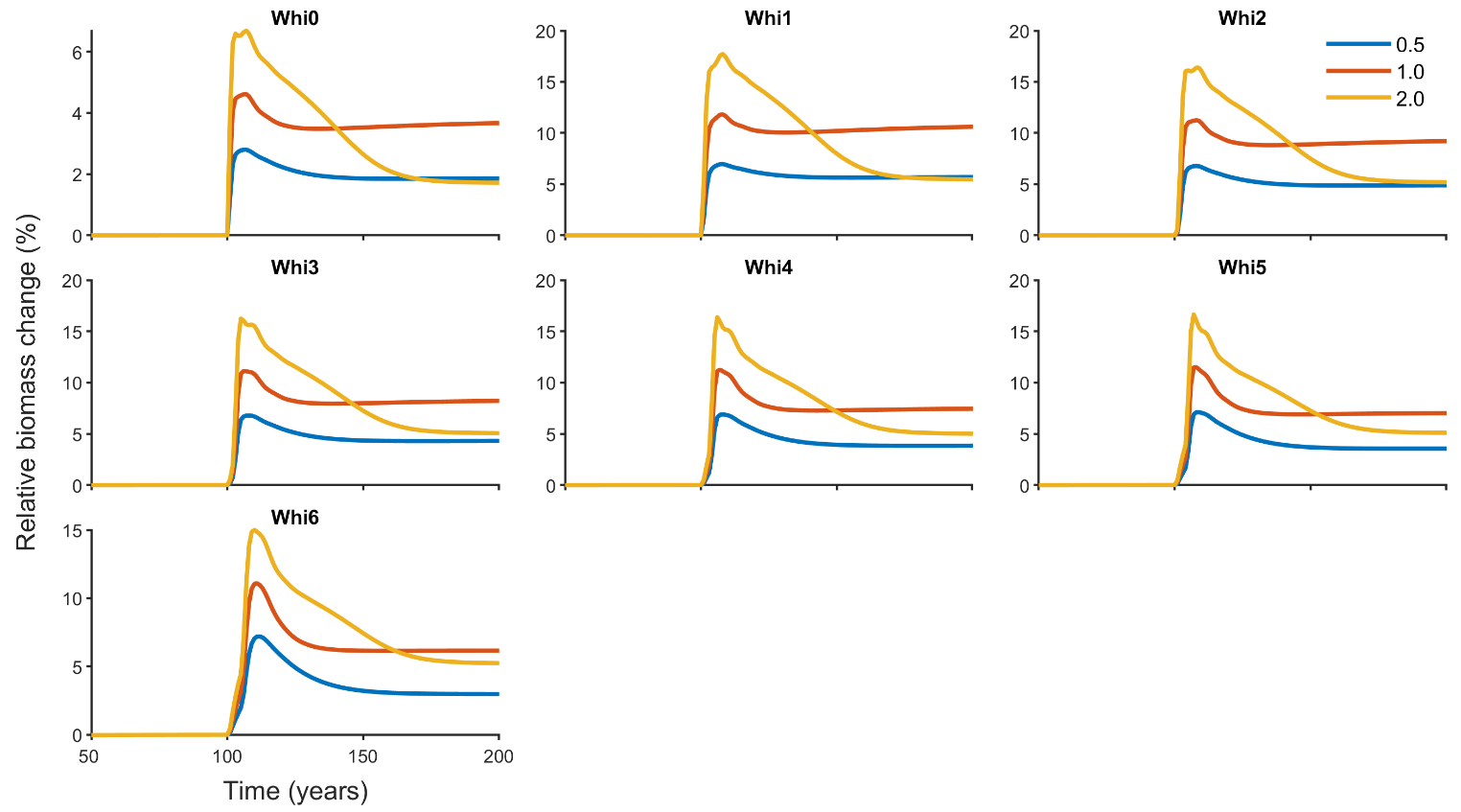


**Figure S52.** Relative biomass change (%) in *Coregonus lavaretus* (Whi) different age guilds, when comparing unfished equilibrium situation into situation with fishing with instantaneous fishing mortalities $0.5y^{-1}$, $1.0y^{-1}$or $2.0y^{-1}$, with no correlation ($\rho$ = 0) between $k$ (Brody’s growth coefficient) and $L^{\infty}$ (asymptotic length) for *Sander lucioperca* and *Coregonus albula*. Whi6 includes ages ≥6.


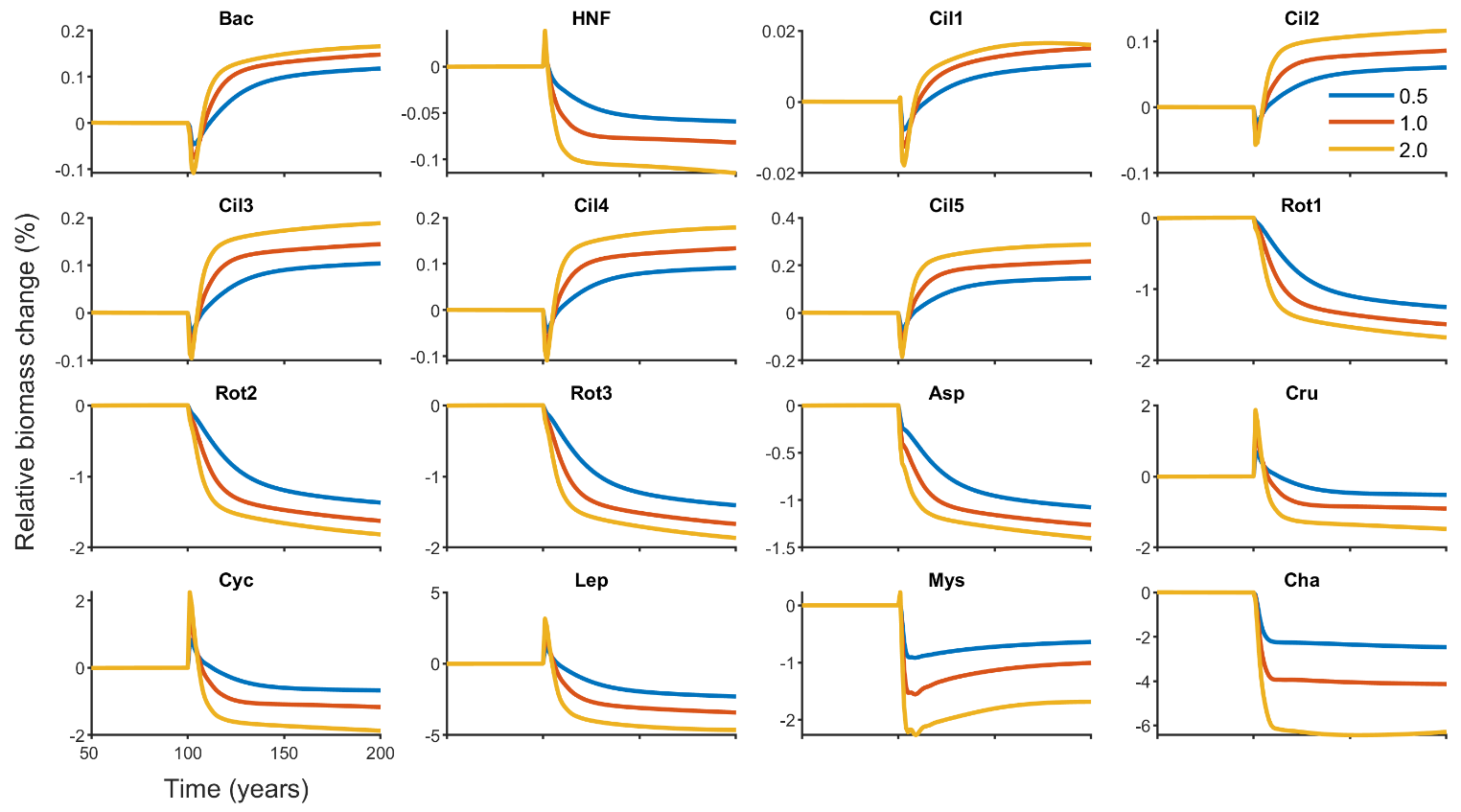
**Figure S53.** Relative biomass change (%) in bacteria (Bac), heterotrophic nanoflagellates (HNF), zooplankton guilds; ciliate group 1 (Cil1), ciliate group 2 (Cil2), ciliate group 3 (Cil3), ciliate group 4 (Cil4), ciliate group 5 (Cil5), rotifer group 1 (Rot1), rotifer group 2 (Rot2), rotifer group 3 (Rot3), *Asplancha priodonta* (Asp), crustacean zooplankton (Cru), cyclopoid zooplankton (Cyc), *Leptodora* and other predatorous zooplankton (Lep) and pelagic invertebrate guilds; *Mysis relicta* (Mys) and *Chaoborus flavicans* (Cha), when comparing unfished equilibrium situation into situation with fishing with instantaneous fishing mortalities $0.5y^{-1}$, $1.0y^{-1}$or $2.0y^{-1}$ , with a high ($\rho$ = -0.7) level of correlation between $k$ (Brody’s growth coefficient) and $L^{\infty}$ (asymptotic length) for *Sander lucioperca* and *Coregonus albula*.


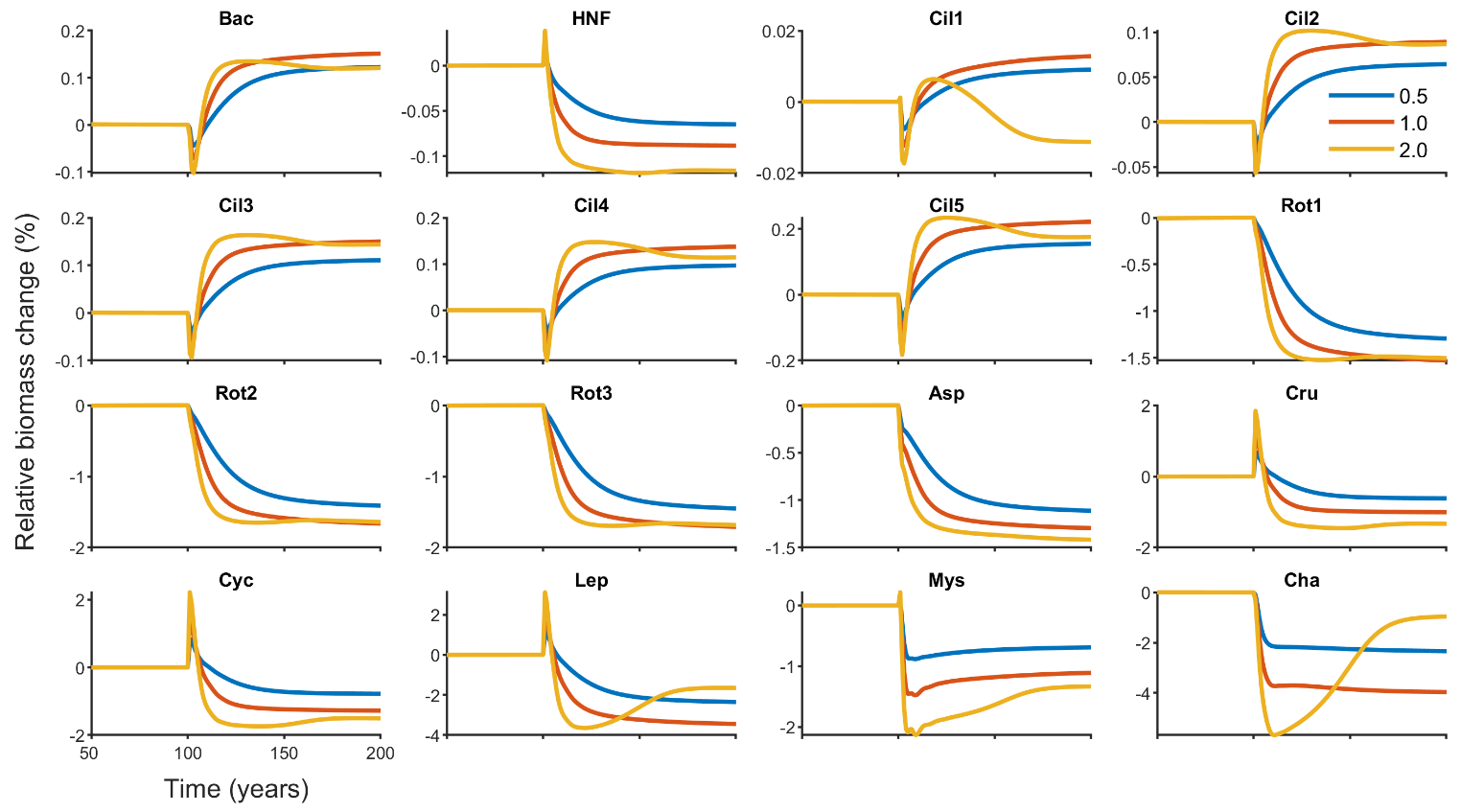
**Figure S54.** Relative biomass change (%) in bacteria (Bac), heterotrophic nanoflagellates (HNF), zooplankton guilds; ciliate group 1 (Cil1), ciliate group 2 (Cil2), ciliate group 3 (Cil3), ciliate group 4 (Cil4), ciliate group 5 (Cil5), rotifer group 1 (Rot1), rotifer group 2 (Rot2), rotifer group 3 (Rot3), *Asplancha priodonta* (Asp), crustacean zooplankton (Cru), cyclopoid zooplankton (Cyc), *Leptodora* and other predatorous zooplankton (Lep) and pelagic invertebrate guilds; *Mysis relicta* (Mys) and *Chaoborus flavicans* (Cha), when comparing unfished equilibrium situation into situation with fishing with instantaneous fishing mortalities $0.5y^{-1}$, $1.0y^{-1}$or $2.0y^{-1}$ , with a low ($\rho$ = -0.35) level of correlation between $k$ (Brody’s growth coefficient) and $L^{\infty}$ (asymptotic length) for *Sander lucioperca* and *Coregonus albula*.


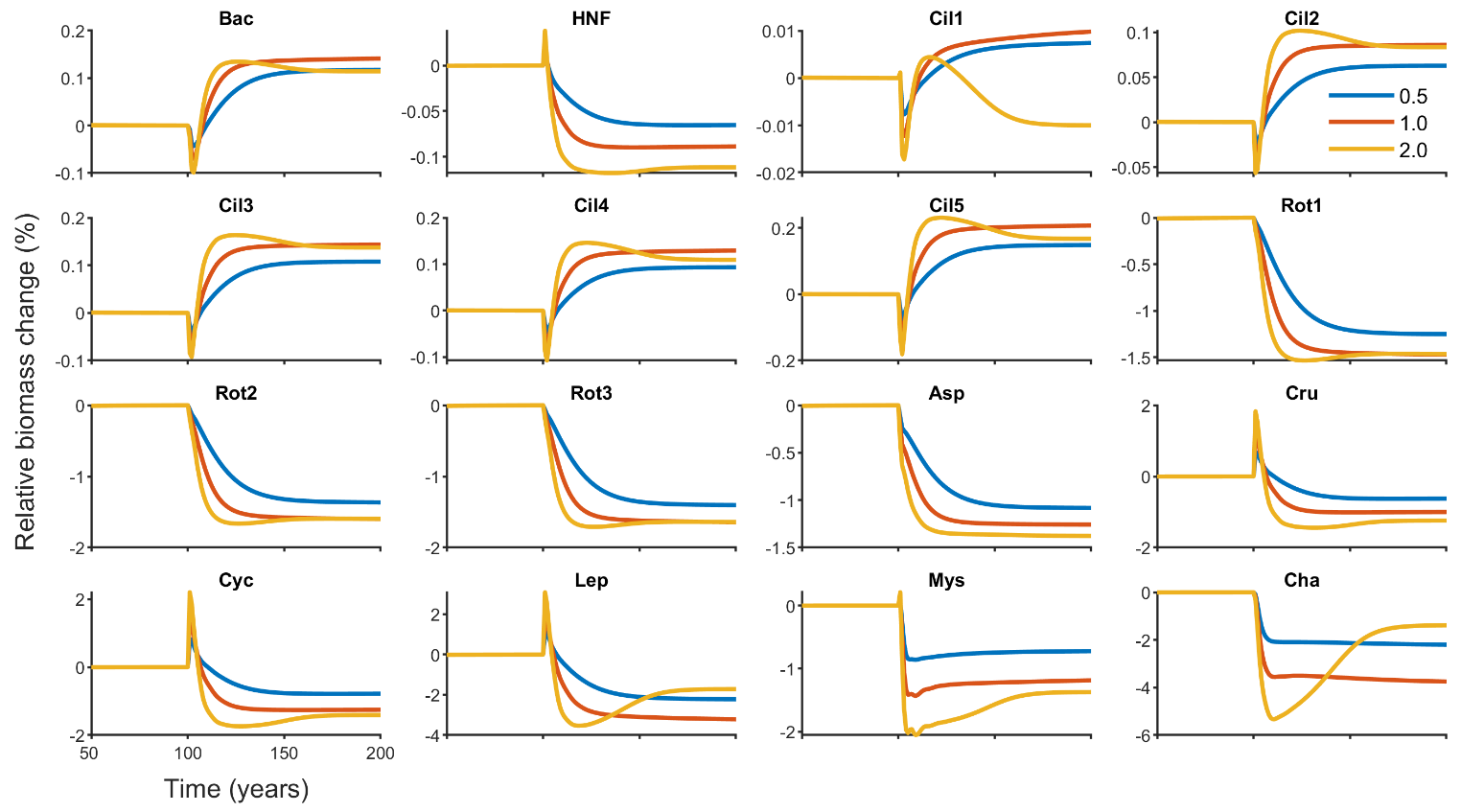


**Figure S55.** Relative biomass change (%) in bacteria (Bac), heterotrophic nanoflagellates (HNF), zooplankton guilds; ciliate group 1 (Cil1), ciliate group 2 (Cil2), ciliate group 3 (Cil3), ciliate group 4 (Cil4), ciliate group 5 (Cil5), rotifer group 1 (Rot1), rotifer group 2 (Rot2), rotifer group 3 (Rot3), *Asplancha priodonta* (Asp), crustacean zooplankton (Cru), cyclopoid zooplankton (Cyc), *Leptodora* and other predatorous zooplankton (Lep) and pelagic invertebrate guilds; *Mysis relicta* (Mys) and *Chaoborus flavicans* (Cha), when comparing unfished equilibrium situation into situation with fishing with instantaneous fishing mortalities $0.5y^{-1}$, $1.0y^{-1}$or $2.0y^{-1}$ , with no correlation ($\rho$ = 0) between $k$ (Brody’s growth coefficient) and $L^{\infty}$ (asymptotic length) for *Sander lucioperca* and *Coregonus albula*.


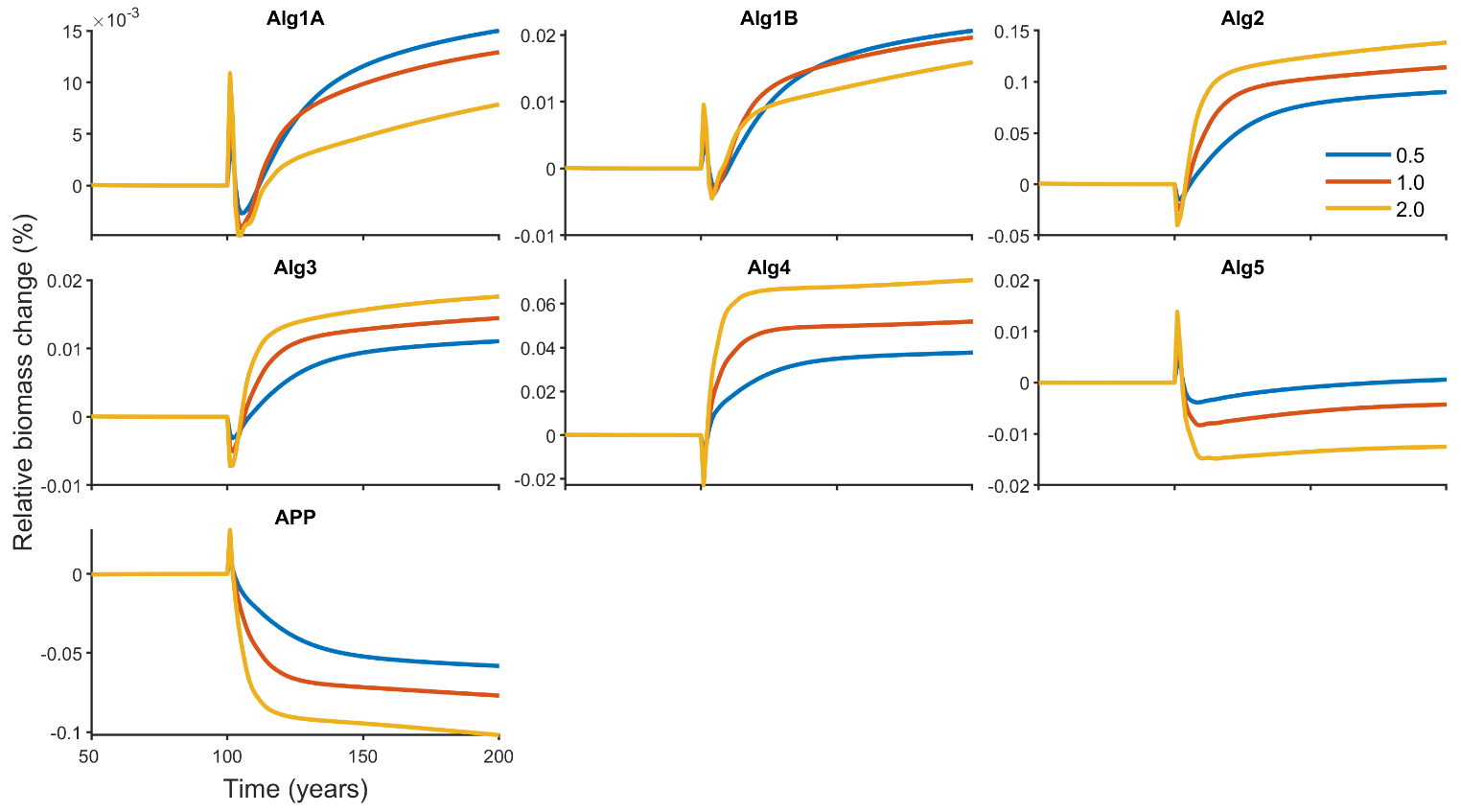
**Figure S56.** Relative biomass change (%) in phytoplankton guilds; especially good quality (high quality fatty acid content) small unicellular phytoplankton ≤ 30 μm (Alg1A), small flagellated unicellular phytoplankton ≤ 30 μm (Alg1B), large unicellular phytoplankton > 30 μm, small poor quality (low quality fatty acid content) unicellular phytoplankton and small colonies (Alg2), bluegreen algae and filamentous green algae (Alg3), diatoms, colonies, filamentous or needle-shaped, long cells, often silicon rich (Alg4), small phytoplankton without flagellates ≤ 30 μm (Alg5) and autotrophic picoplankton (APP), when comparing unfished equilibrium situation into situation with fishing with instantaneous fishing mortalities $0.5y^{-1}$, $1.0y^{-1}$or $2.0y^{-1}$, with a high ($\rho$ = -0.7) level of correlation between $k$ (Brody’s growth coefficient) and $L^{\infty}$ (asymptotic length) for *Sander lucioperca* and *Coregonus albula*.


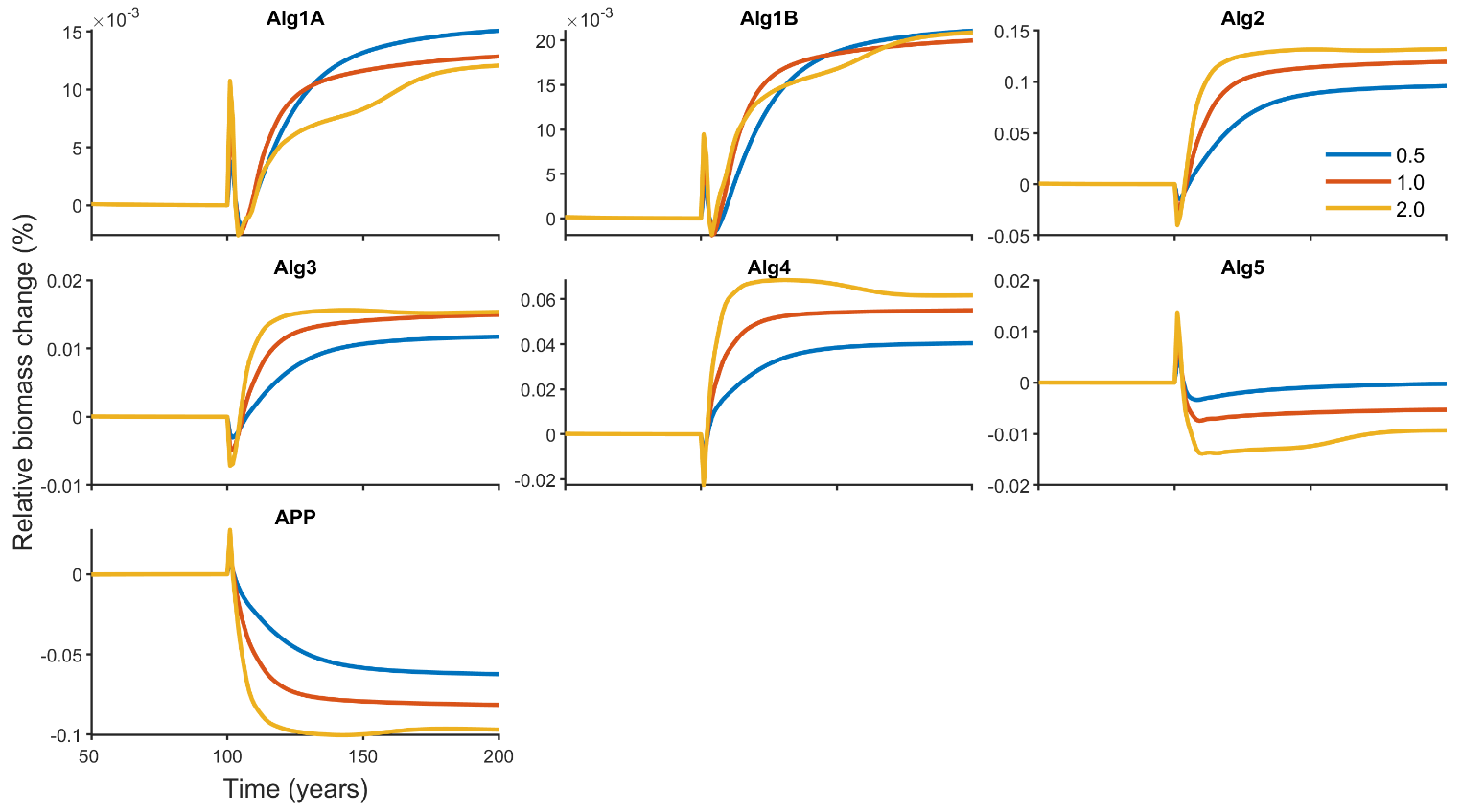
**Figure S57.** Relative biomass change (%) in phytoplankton guilds; especially good quality (high quality fatty acid content) small unicellular phytoplankton ≤ 30 μm (Alg1A), small flagellated unicellular phytoplankton ≤ 30 μm (Alg1B), large unicellular phytoplankton > 30 μm, small poor quality (low quality fatty acid content) unicellular phytoplankton and small colonies (Alg2), bluegreen algae and filamentous green algae (Alg3), diatoms, colonies, filamentous or needle-shaped, long cells, often silicon rich (Alg4), small phytoplankton without flagellates ≤ 30 μm (Alg5) and autotrophic picoplankton (APP), when comparing unfished equilibrium situation into situation with fishing with instantaneous fishing mortalities $0.5y^{-1}$, $1.0y^{-1}$or $2.0y^{-1}$, with a low ($\rho$ = -0.35) level of correlation between $k$ (Brody’s growth coefficient) and $L^{\infty}$ (asymptotic length) for *Sander lucioperca* and *Coregonus albula*.


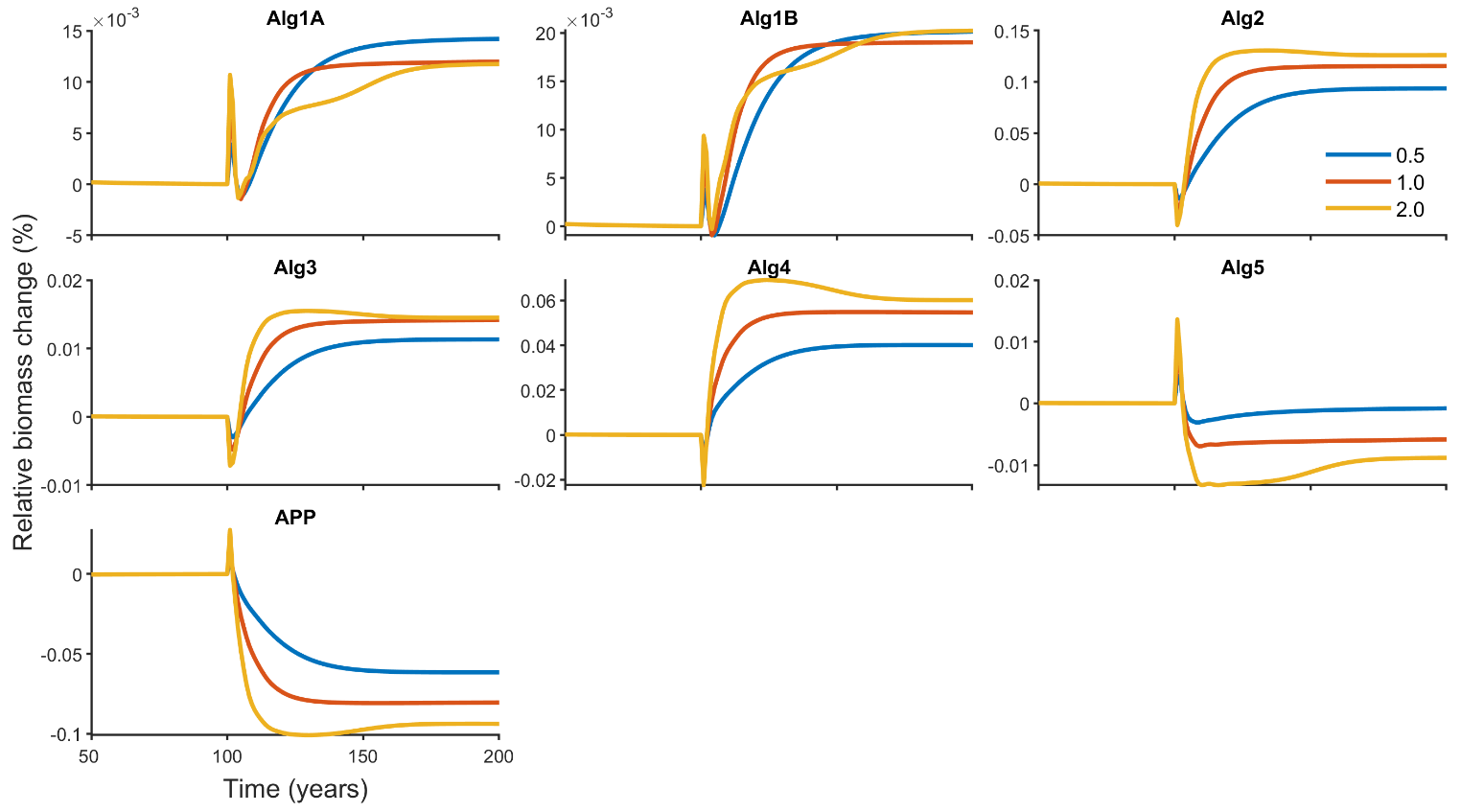


**Figure S58.** Relative biomass change (%) in phytoplankton guilds; especially good quality (high quality fatty acid content) small unicellular phytoplankton ≤ 30 μm (Alg1A), small flagellated unicellular phytoplankton ≤ 30 μm (Alg1B), large unicellular phytoplankton > 30 μm, small poor quality (low quality fatty acid content) unicellular phytoplankton and small colonies (Alg2), bluegreen algae and filamentous green algae (Alg3), diatoms, colonies, filamentous or needle-shaped, long cells, often silicon rich (Alg4), small phytoplankton without flagellates ≤ 30 μm (Alg5) and autotrophic picoplankton (APP), when comparing unfished equilibrium situation into situation with fishing with instantaneous fishing mortalities $0.5y^{-1}$, $1.0y^{-1}$or $2.0y^{-1}$, with no correlation ($\rho$ = 0) between $k$ (Brody’s growth coefficient) and $L^{\infty}$ (asymptotic length) for *Sander lucioperca* and *Coregonus albula*.
